# Quorum Sensing Inhibits Type III‐A CRISPR‐Cas System Activity Through Repressing Positive Regulators SarA and ArcR in *Staphylococcus Aureus*

**DOI:** 10.1002/advs.202506049

**Published:** 2025-07-11

**Authors:** Yang Li, Yuanyue Tang, Xiaofei Li, Nina Molin Høyland‐Kroghsbo, Hanne Ingmer, Xinan Jiao, Qiuchun Li

**Affiliations:** ^1^ Jiangsu Key Lab of Zoonosis/Jiangsu Co‐Innovation Center for Prevention and Control of Important Animal Infectious Diseases and Zoonoses Yangzhou University Jiangsu 225100 China; ^2^ Key Laboratory of Prevention and Control of Biological Hazard Factors (Animal Origin) for Agri‐food Safety and Quality Ministry of Agriculture of China Yangzhou University Jiangsu 225100 China; ^3^ Department of Plant and Environmental Sciences University of Copenhagen Copenhagen 1870 Denmark; ^4^ Department of Veterinary and Animal Sciences University of Copenhagen Copenhagen 1870 Denmark; ^5^ Joint International Research Laboratory of Agriculture and Agri‐Product Safety Yangzhou University Jiangsu 225100 China

**Keywords:** AcrR, quorum sensing (QS), SarA, *Staphylococcus aureus*, type III‐A CRISPR‐Cas system

## Abstract

CRISPR‐Cas is an adaptive immune system that protects prokaryotes from the invasion of foreign genetic elements. The components and immunity mechanisms of CRISPR‐Cas have been extensively studied, but the regulation of this system in *Staphylococci* remains unclear. Here, it is shown that the cell‐cell communication, known as quorum sensing (QS), inhibits the expression and activity of the type III‐A CRISPR‐Cas system in *S. aureus*. The QS regulator, AgrA, directly binds to the promoters of two transcriptional regulators encoding the genes *sarA* and *arcR* to inhibit their expression. However, both SarA and ArcR act as positive regulators that promote the transcription of *cas* genes by directly binding to a novel promoter P*cas*. Furthermore, the P*cas* of 300 bp located in *cas1* displays as a critical regulatory node to initiate the transcription of *cas10* and *csm3*. Our data reveal a new regulatory mechanism for QS‐mediated repression of the Type III‐A CRISPR‐Cas system, which may allow *S. aureus* to acquire foreign genetic elements encoding antibiotic resistance or virulence factors specifically at high cell density.

## Graphical abstract

This study proposes a novel model illustrating how quorum sensing (QS) regulates the type III‐A CRISPR‐Cas system in *Staphylococcus aureus*. The QS regulator AgrA suppresses CRISPR‐Cas activity by downregulating the transcriptional regulators SarA and ArcR. This QS‐mediated repression may promote the acquisition of foreign genetic elements, including antibiotic resistance and virulence genes.

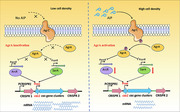

## 1 Introduction

Bacterial viruses, the bacteriophages (or phages), are the most abundant organisms on earth and play a crucial role in the population dynamics of microbes in the majority of ecosystems.^[^ ^1^ ^]^ Depending on their lifestyle, phages can be divided into lytic and temperate phages. The lytic phages are commonly used in phage therapy due to their ability to specifically infect and kill bacteria. In response to phage infection, bacteria have evolved multiple lines of innate and adaptive immune systems, including restriction‐modification, abortive infection, and CRISPR‐Cas systems,^[^ ^2^ ^]^ which are major limitations of phage therapy. The CRISPR‐Cas system is an adaptive immune system in archaea and bacteria that defends against the invasion of foreign nucleic acids, such as phages (viruses) and plasmids.^[^ ^3^, ^4^ ^]^ CRISPR‐Cas systems have been classified into six types (I‐VI) with 52 subtypes.^[^ ^5^ ^]^ Types I, III, and IV are composed of multi‐subunit effector complexes, whereas types II, V, and VI harbor a single effector protein that cleaves target nucleic acids.^[^ ^5^ ^]^ The defense processes of CRISPR‐Cas systems consist of three distinct stages: adaptation, CRISPR RNA (crRNA) biogenesis, and interference.^[^ ^6^ ^]^ During adaptation, the invading nucleic acids derived from plasmids or phages are recognized and incorporated into CRISPR arrays.^[^ ^7^, ^8^ ^]^ The CRISPR array is transcribed into a long precursor CRISPR RNA (pre‐crRNA), which is subsequently processed into mature crRNAs.^[^ ^9^, ^10^ ^]^ Finally, the Cas proteins in combination with mature crRNAs form an effector complex to degrade the invading genetic elements.^[^ ^11^ ^]^ Unlike other CRISPR‐Cas types, the type III CRISPR‐Cas system requires transcription of target sequences recognized by crRNAs for effective immunity.^[^ ^12^, ^13^, ^14^ ^]^ The binding of target RNA to the effector complex triggers activation of the Cas10 HD and palm domains. The activated HD domain can non‐specifically cleave ssDNA,^[^ ^15^, ^16^, ^17^ ^]^ and the palm domain is responsible for the synthesis of cyclic oligoadenylates, which act as a secondary messenger to activate Csm6 or Csx1 RNase for non‐specific RNA degradation.^[^ ^18^, ^19^ ^]^ The type III‐A CRISPR‐Cas system has been discovered at a frequency of 6.8% in *Staphylococci* species, including *S. aureus* and *S. epidermidis*.^[^ ^4^, ^20^, ^21^, ^22^ ^]^ Furthermore, this system has been demonstrated to be functional in protecting against plasmid invasion and phage infection in both *S. aureus* and *S. epidermidis*,^[^ ^4^, ^13^, ^21^ ^]^ which poses a challenge for phage therapy. In the past decade, regulation of CRISPR‐Cas systems has been frequently reported in Gram‐negative bacteria,^[^ ^23^, ^24^ ^]^ but few studies have focused on how the activity of these systems is regulated in Gram‐positive bacteria.

**Figure 1:**
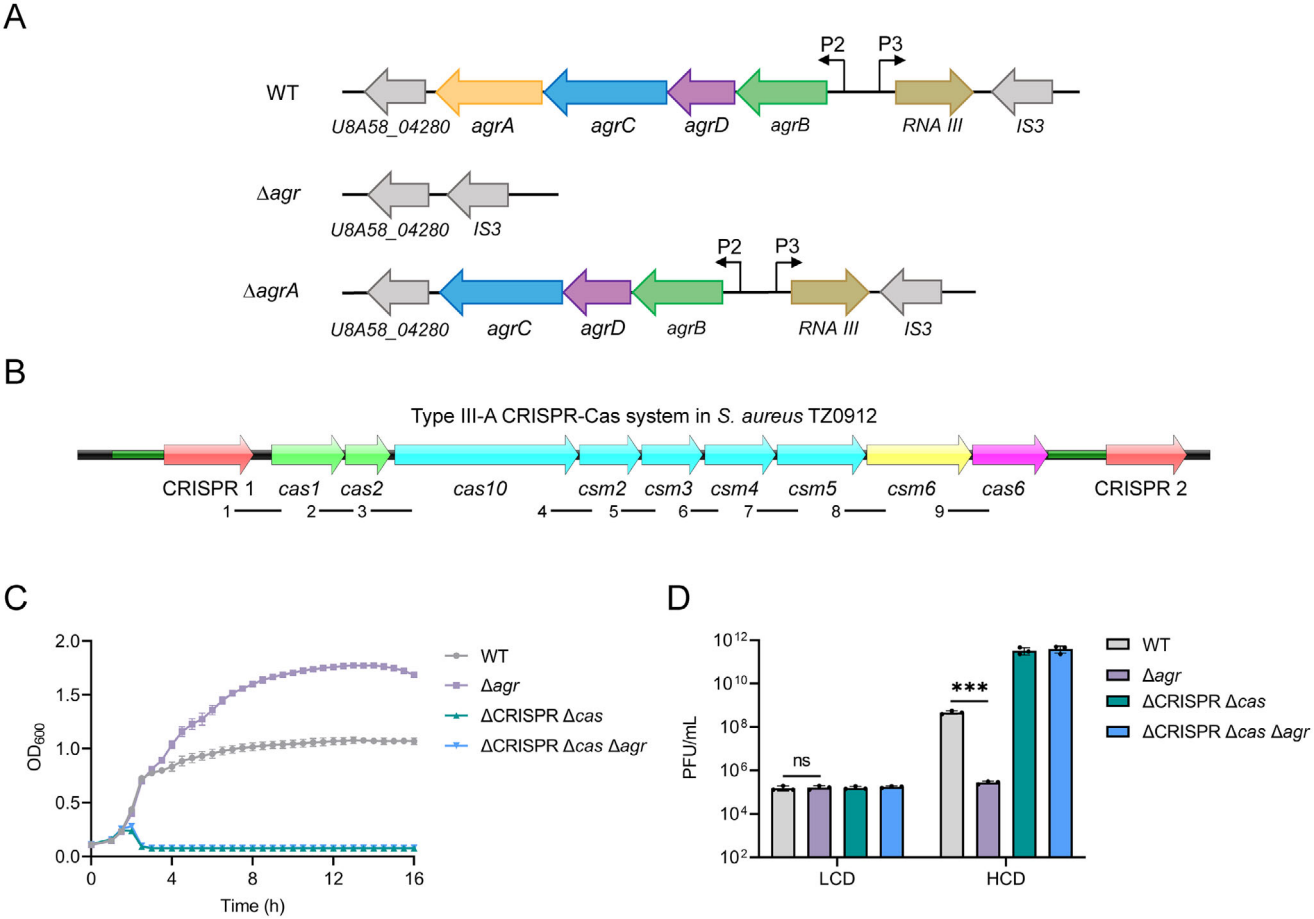
*S. aureus* QS *agr* system is involved in phage susceptibility. A) Schematic diagram of QS *agr* system in *S. aureus* TZ0912 strain. The QS *agr* system consists of the *agrBDCA* operon and *RNA III* gene, which are indicated by distinct colored arrows. The ∆*agr* and ∆*agrA* mutants are also represented. B) Schematic diagram of type III‐A CRISPR‐Cas system in *S. aureus* TZ0912 strain. The nine fragments (number 1 to 9) corresponding to intergenic regions and amplified in the RT‐PCR experiments are indicated below. The leader of CRISPR 1 and CRISPR 2 array were indicated as dark green lines. C) Growth curve of the WT, ∆*agr*, ∆CRISPR ∆*cas*, and ∆CRISPR ∆*cas* ∆*agr* strains infected with phage phiIPLA‐RODI at an MOI of 0.01. D) Phage titers were measured at LCD and HCD after infection with phage phiIPLA‐RODI at an MOI of 0.01 on the WT, ∆*agr*, ∆CRISPR ∆*cas*, and ∆CRISPR ∆*cas* ∆*agr* strains. All the data shown above (C and D) are means ± standard deviation of three independent experiments. A two‐tailed unpaired Student's *t*‐test was used to calculate *P* values; ns, not significant, ^***^ *p* < 0.001.

In nature, the risk of phage infection increases with the bacterial cell density. Accordingly, bacteria employ a cell‐cell communication process known as QS to regulate antiphage defense strategies. Previous studies have demonstrated that CRISPR‐Cas is positively regulated by QS in some Gram‐negative bacteria.^[^ ^25^, ^26^, ^27^, ^28^, ^29^, ^30^ ^]^ Specifically, LuxI/R‐type QS systems activate *cas* genes expression in the Gram‐negative bacterium *Burkholderia glumae* ^[^ ^27^ ^]^ while CRISPR‐Cas interference and CRISPR adaptation is activated in response to LuxR/I QS in both *Serratia* and *Pseudomonas aeruginosa*.^[^ ^25^, ^26^ ^]^ QS has also been shown to block phage infection in Gram‐negative bacteria via downregulating phage receptors.^[^ ^31^, ^32^ ^]^ In contrast, the QS *agr* system in Gram‐positive *S. aureus* enhances phage adsorption by reducing *tarM*‐encoded α‐GlcNAc glycosylation modification of wall teichoic acid, thereby promoting phage infection.^[^ ^33^ ^]^ However, it is currently unknown whether QS regulates CRISPR‐Cas systems in response to phage infection in Gram‐positive bacteria. In *S. aureus*, the QS *agr* system consists of the *agrBDCA* operon and *RNA III* gene, which are under the control of the P2 and P3 promoters, respectively (**Figure** **1**A). The *agrD* gene encodes the precursor of QS signal molecule, called autoinducing peptides (AIPs).^[^ ^34^ ^]^ AgrB is a transmembrane endopeptidase responsible for processing AgrD into AIPs and exporting them outside the cell.^[^ ^35^ ^]^ These AIPs are sensed by a two‐component signal transduction system consisting of a sensory histidine kinase AgrC and a transcriptional response regulator AgrA.^[^ ^36^ ^]^ At high cell density, AgrA is activated by AgrC‐dependent phosphorylation, after which it directly binds to P2 and P3 promoter, leading to elevated AIPs production and a positive feedback loop.^[^ ^34^ ^]^ *RNA III* not only encodes a virulence factor δ‐toxin (Hld) but also functions as a small regulatory RNA, ^[^ ^37^ ^]^ which upregulates genes encoding secreted proteins, such as toxins and exoenzymes, and downregulates several genes encoding surface‐associated adhesins.^[^ ^38^, ^39^ ^]^

In this study, we observed that increasing cell density led to decreased expression of *cas* genes in *S. aureus*, reducing CRISPR‐Cas‐mediated antiphage immunity, and thus rendering the bacteria more susceptible to phage infection. We demonstrated that QS response regulator, AgrA, indirectly represses the expression of *cas* genes. Specifically, AgrA negatively regulates the expression of two transcriptional regulators encoded by *sarA* and *arcR*. In *S. aureus*, SarA is a major global transcriptional regulator that coordinates the expression of many virulence genes^[^ ^40^ ^]^ while ArcR is a member of the cAMP receptor protein (CRP) family of transcriptional regulators and is involved in the arginine catabolic pathway.^[^ ^41^ ^]^ We further showed that SarA and ArcR directly bind to and stimulate expression from the CRISPR1 and *cas* promoter regions. Thus, at high cell density, AgrA‐mediated repression of *sarA* and *arcR* results in decreased *cas* genes expression and CRISPR‐Cas‐mediated interference activity in *S. aureus*. Our study implies that QS inhibition could reduce the efficacy of phage therapy against CRISPR‐Cas positive *S. aureus*.

## 2 Results

### 2.1 Quorum Sensing Promotes Phage Infection in *S. aureus*

To investigate whether *S. aureus* QS *agr* system affects bacterial susceptibility to phage infection, we used a clinical Methicillin‐resistant *Staphylococcus aureus* (MRSA) strain TZ0912, which carries a complete type III‐A CRISPR‐Cas system with two CRISPR arrays, as the model (Figure 1B).^[^ ^21^ ^]^ We constructed an ∆*agr* mutant by inactivating the *agrBDCA* operon and the *RNA III* gene (Figure 1A), and exposed the WT and ∆*agr* strains to phage philPLA‐RODI, which is targeted by spacer 6 (*sp6*) at the CRISPR1 locus of WT (Figure S1A, Supporting Information). The bacterial growth was subsequently monitored over time in liquid culture. We found that the ∆*agr* mutant showed a stronger survival ability compared to WT during phage infection, indicating the involvement of QS in phage susceptibility (Figure 1C). To characterize the growth phase of the TZ0912 strain at low cell density (LCD) and high cell density (HCD), we monitored CFU counts and *agrA* expression levels for QS signal every 2 h. At 2 h, the cell density was 3.63×10^8^ CFU mL^−1^ with a low *agrA* expression level. The expression of *agrA* reached a peak at 10 h with a cell density of 5.20×10^9^ CFU mL^−1^, while *agrA* expression decreased after 10 h (Figure S1B, Supporting Information). Therefore, we used the cultures for 2 and 10 h to represent the LCD and HCD, respectively. Subsequently, we examined the phage titers in WT and ∆*agr* strains at LCD and HCD after infection. No differences in either number or size of the phage plaques were observed when the WT and ∆*agr* strains were at LCD (Figure 1D). However, the ∆*agr* mutant exhibited a reduction in phage titer of 10^3^‐fold compared to WT at HCD (Figure 1D), highlighting the critical role of QS *agr* system in phage susceptibility. Taken together, these results indicate that the QS *agr* system promotes phage infection in *S. aureus*, consistent with previous results.^[^ ^33^ ^]^

### 2.2 Quorum Sensing Represses Expression of *Cas* Genes in the Type III‐A CRISPR‐Cas System

Since phage adsorption is the first step for phages to recognize and infect bacteria, it is possible that *agr* influences this process and consequently promotes phage infection. However, we observed that the WT and ∆*agr* strains showed similar phage adsorption rates at both LCD and HCD (Figure S1C, Supporting Information), suggesting that *agr*‐mediated susceptibility to phage infection is not accomplished by altering the adsorption rate, which contrasts with previous observations in other bacterial species.^[^ ^31^, ^32^, ^33^, ^42^ ^]^ Given that the TZ0912 strain used in this study harbors a complete type III‐A CRISPR‐Cas system with *sp6* of CRISPR1 matching the phage philPLA‐RODI, we hypothesized that the effect of *agr* on susceptibility to phage infection may be dependent on the CRISPR‐Cas activity. To test this, we constructed ∆CRISPR ∆*cas* and ∆CRISPR ∆*cas* ∆*agr* mutants, and monitored bacterial growth during phage philPLA‐RODI infection. As predicted, both mutants succumbed to phage infection and lysed rapidly (Figure 1C). Corroborating this result, the supernatants of ∆CRISPR ∆*cas* and ∆CRISPR ∆*cas* ∆*agr* cultures formed the same number of plaques at LCD and HCD when we measured the phage titers in both strains after infection (Figure 1D). These results indicate that *S. aureus* QS *agr* system promotes phage infection by modulating CRISPR‐Cas activity.

**Figure 2:**
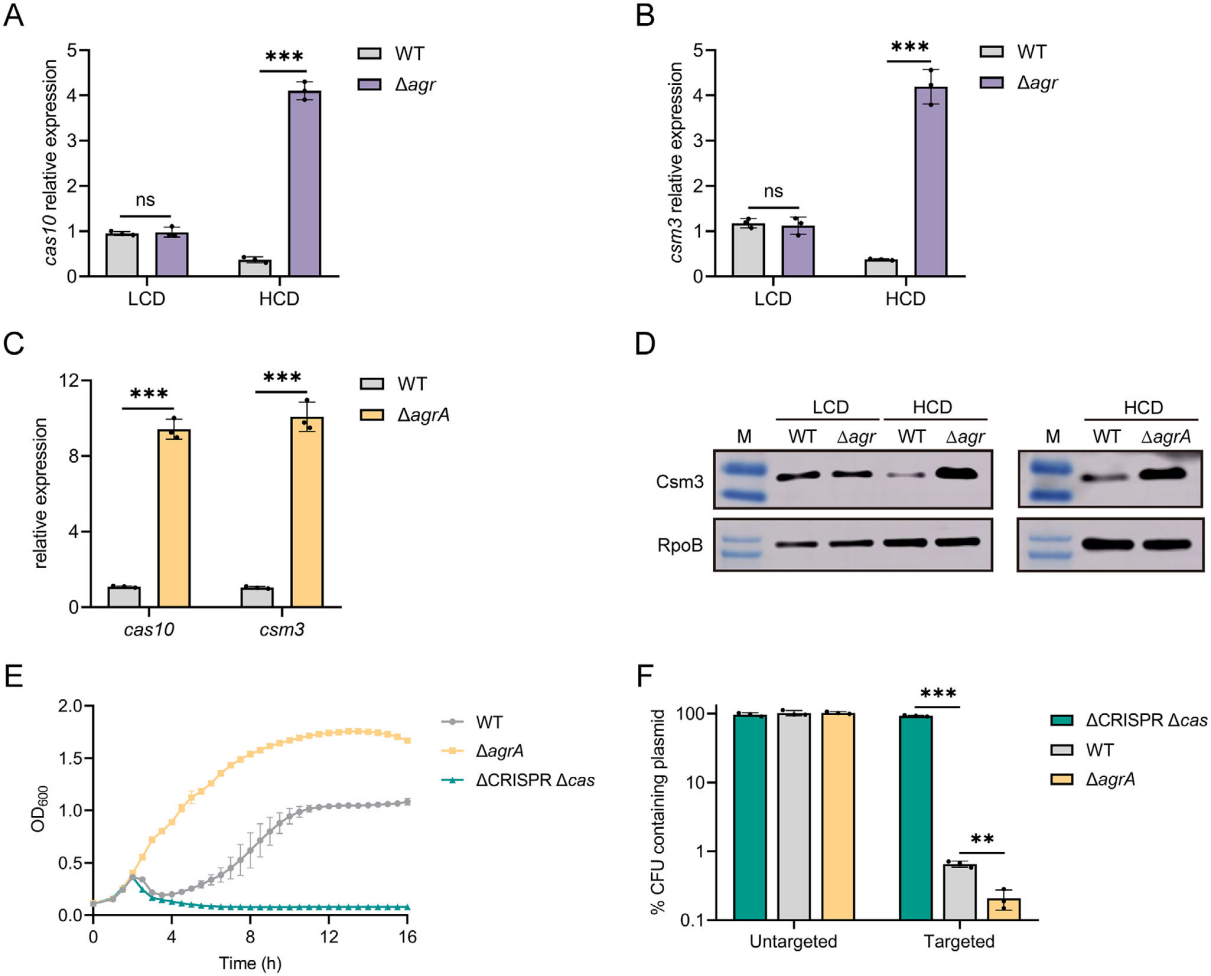
QS represses the expression and interference of type III‐A CRISPR‐Cas system in *S. aureus*. A and B) qRT‐PCR analysis of *cas10* and *csm3* expression in the WT and ∆*agr* mutant at LCD and HCD after infection with phage phiIPLA‐RODI at an MOI of 0.01. C) qRT‐PCR analysis of *cas10* and *csm3* expression in the WT and ∆*agrA* mutant at HCD after infection with phage phiIPLA‐RODI at an MOI of 0.01. D) Western blot analysis of Csm3 protein level in the WT, ∆*agr*, and ∆*agrA* mutant strains at LCD or HCD after infection with phage phiIPLA‐RODI at an MOI = 0.01. The expression level of Csm3 was determined using that of RpoB as the internal control. Western blot assays were replicated three times, and representative images are shown. E) Growth curve of the WT, ∆*agrA*, and ∆CRISPR ∆*cas* strains infected with phage phiIPLA‐RODI at an MOI of 0.01. F) Retention of the untargeted plasmid pRMC2 or targeted plasmid pCR1SP1 in the WT, ∆CRISPR ∆*cas* and ∆*agrA* mutant. The strains containing the plasmid were grown in TSB with ATc for 10 h followed by plating on TSA with or without chloramphenicol. % CFUs containing plasmid were scored as the number of Cm^R^ CFU/the total number of CFU. All the data shown above (A‐C, E and F) are means ± standard deviation of three independent experiments. A two‐tailed unpaired Student's *t*‐test was used to calculate *P* values; ns, not significant, ^**^ *p* < 0.01, ^***^ *p* < 0.001.

Next, to explore whether QS is involved in the regulation of CRISPR‐Cas system in *S. aureus*, we quantified *cas* genes expression (*cas10* and *csm3*) in the WT and ∆*agr* strains infected by phage philPLA‐RODI at LCD and HCD using qRT‐PCR. Both *cas10* and *csm3* genes were highly expressed in WT at LCD, while their expression was decreased at HCD (**Figure** **2**A,B), suggesting that the expression of these genes may be repressed in response to QS signaling at HCD. Interestingly, the expression of both *cas* genes was similar in WT and ∆*agr* mutant cells at LCD, whereas at HCD, expression of these two genes was significantly higher (10‐fold) in the ∆*agr* mutant compared to WT (Figure 2A,B). Similar results were obtained when we measured the *cas* genes expression in both strains without phage infection (Figure S2A,B, Supporting Information). Since QS signaling molecule AIPs can accumulate in the growth medium from HCD culture, we cultured the WT and ∆*agr* mutants to LCD in the TSB medium supplemented with HCD culture supernatant (termed as HCD TSB), and *cas* gene expression was then quantified by qRT‐PCR. In the WT, this treatment led to a significant reduction in *cas* genes expression compared to cells grown in fresh medium (Figure S2C,D, Supporting Information). However, no significant change was observed in the ∆*agr* mutant (Figure S2C,D, Supporting Information). Consistent with the reduced *cas* genes expression, WT cells grown in HCD TSB exhibited increased phage susceptibility (Figure S2E, Supporting Information). Taken together, these results suggest that QS plays a negative role in regulating the type III‐A CRISPR‐Cas system.

To identify the key determinant in the *agr* system regulating the expression of the CRISPR‐Cas system, mutant strains lacking the *agrA*, *agrB*, *agrC*, *agrD*, and *RNA III* were generated and tested for CRISPR‐*cas* expression at HCD (Figure 1A). Compared with the WT, the deletion of *RNA III* has no effect on the *cas* genes and *crispr1/2* expression (Figure S2F, Supporting Information). In contrast, deletion of *agrB*, *agrC*, or *agrD* resulted in only a slight upregulation of *cas* genes and *crispr1* (Figure S2F, Supporting Information), whereas the deletion of *agrA* increased the expression of *cas* genes to the same level as that in the ∆*agr* mutant at HCD (Figure 2C), indicating that AgrA plays a dominant role in repressing CRISPR‐Cas system in the *agr* system. Furthermore, the *crispr1* expression was significantly higher in the ∆*agrA* mutant than in the WT (5‐fold), whereas the expression of *crispr2* in the ∆*agrA* mutant was not significantly affected (Figure S2G, Supporting Information). To determine whether QS regulates *cas* genes expression at the protein level, we performed Western blot analysis using Csm3 as an indicator protein. Consistent with the qRT‐PCR transcriptional data, Csm3 protein was significantly elevated in the ∆*agrA* mutant compared to WT at HCD (Figure 2D). Overall, these data suggest that QS regulation of the type III‐A CRISPR‐Cas system is mediated by AgrA, which functions as a repressor of *cas* genes.

### 2.3 Quorum Sensing Regulates Type III‐A CRISPR‐Cas Activity

In the light of observation that QS global regulator AgrA inhibits the CRISPR‐Cas expression, we sought to determine whether AgrA has effects on CRISPR‐Cas activity in defending against phages. To this end, we infected the WT, ∆CRISPR ∆*cas*, and ∆*agrA* strains with phage philPLA‐RODI. The *Staphylococcus* phage phiSA012, without homology to any spacers in WT, was used as a non‐CRISPR targeted control. As expected, all these strains were sensitive to untargeted phage phiSA012 (Figure S2H, Supporting Information). In contrast, we observed that the *agrA* mutant exhibited enhanced resistance to phage philPLA‐RODI compared to WT (Figure 2E), suggesting that AgrA facilitates phage infection by reducing the CRISPR‐Cas interference activity. Given that AgrD and AgrB are responsible for the synthesis and mature of AIPs, which can activate AgrA, we infected the WT, ∆CRISPR ∆*cas*, ∆*agrA*, ∆*agrB*, and ∆*agrD* mutant strains with phage philPLA‐RODI to test whether AgrD and AgrB also influence the CRISPR‐Cas immunity. Compared to the WT strain, ∆*agrB* and ∆*agrD* mutants showed an increased immunity against phage infection, but their phage resistance was weaker than that of the ∆*agrA* mutant. This is consistent with the *cas* genes expression pattern in these strains and further confirms that AgrA is the key regulator of the CRISPR‐Cas system in the *agr* system. Additionally, to test whether chemical inhibition of the *agr* system can influence CRISPR‐Cas activity, we exposed the WT, ∆CRISPR ∆*cas*, and ∆*agrA* strains to phage philPLA‐RODI in the presence or absence of 3‐hydroxybenzoic acid (3‐HBA), a known AgrA inhibitor.^[^ ^43^ ^]^ The results showed that treatment with 3‐HBA enhanced the resistance of the WT strain to phage infection, but had no effect on the phage susceptibility of the ∆*agrA* mutant (Figure S2H, Supporting Information), further supporting the regulatory role of AgrA in repressing CRISPR‐Cas activity.

To assess the effect of AgrA on the ability of the CRISPR‐Cas system in eliminating invading plasmids, we measured the retention of an untargeted plasmid pRMC2 and a CRISPR‐targeted plasmid pCR1SP1, which carries a protospacer complementary to spacer 1 (*sp1*) in the CRISPR 1 locus of TZ0912, in the WT, ∆CRISPR ∆*cas*, and ∆*agrA* strains. We used anhydrotetracycline (ATc) to induce the Pxy/tet‐inducible promoter that drives transcription of the plasmid protospacer. The untargeted plasmid was completely retained in all strains (Figure 2F). In contrast, we observed a robust clearance of the pCR1SP1 plasmid in the WT, confirming that the WT had effective CRISPR‐Cas activity against the targeted plasmid. The ∆*agrA* mutant showed a 3‐fold decrease in the retention of the pCR1SP1 plasmid compared to WT (Figure 2F), indicating that AgrA represses the interference activity of the type III‐A CRISPR‐Cas system against the plasmid. Collectively, these results demonstrate that AgrA represses type III‐A CRISPR‐Cas interference in *S. aureus*, and thereby promoting phage infections and plasmid invasions.

### 2.4 Identification of Promoters for the CRISPR1 Array and *Cas* Gene Cluster

**Figure 3:**
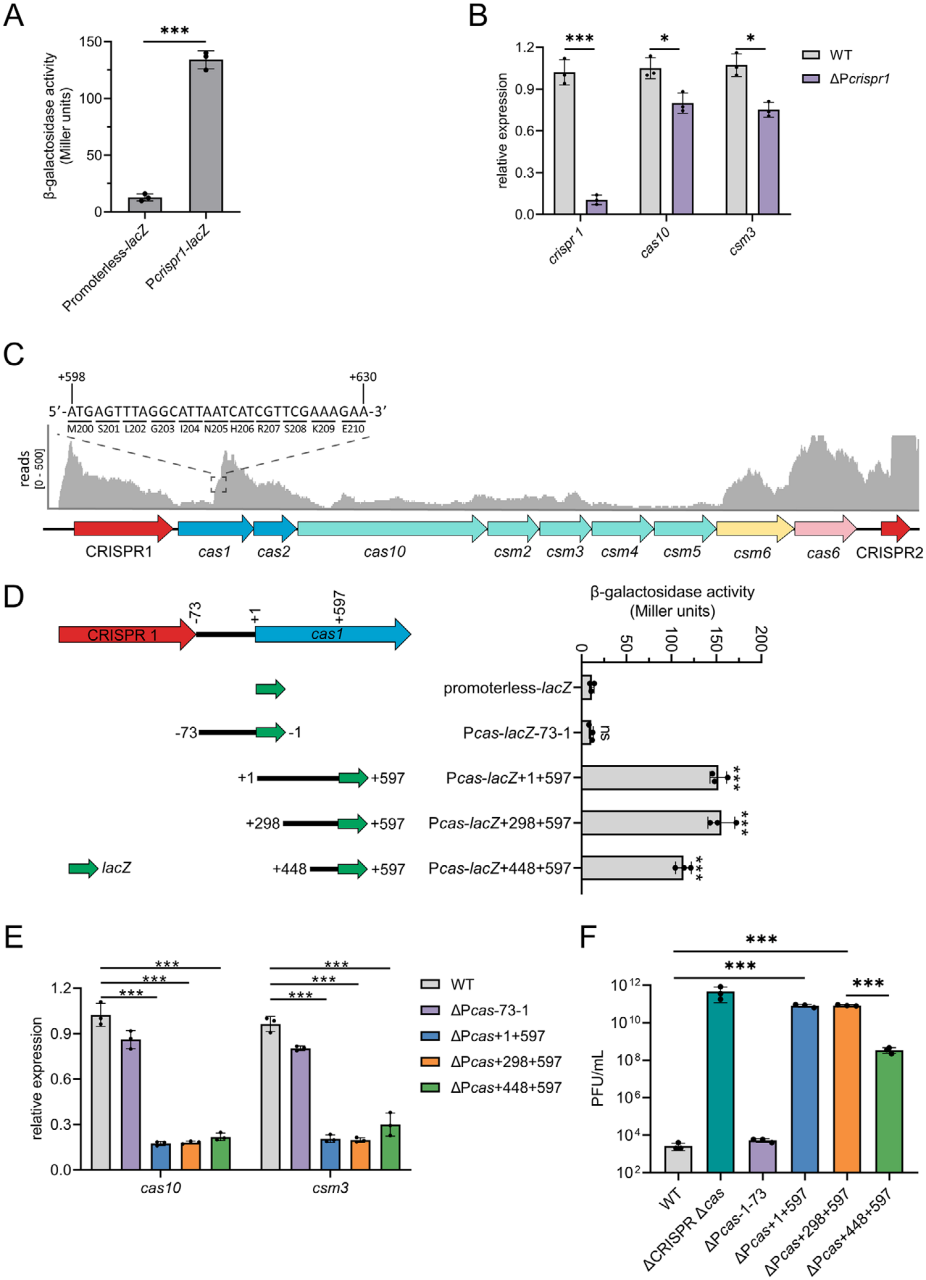
The P*crispr1* sequence and a promoter located within the *cas1* gene promote transcription of CRISPR1 and *cas*, respectively. A) β‐galactosidase activity of a promoterless *lacZ* fusion and of the P*crispr1*‐*lacZ* reporter fusion in the WT. B) qRT‐PCR of *crispr*, *cas10* and *csm3* expression in the WT and in a ∆P*crispr1* mutant, respectively. C) RNA‐seq reads were mapped to the WT genome to determine the relative abundance of the complete type III‐A CRISPR‐Cas system expression by IGV software. An ATG at the (position between +598 to +600) is located within the *cas1* encoding region. D) Transcriptional fusions are represented as lines with a short green arrow at their 3′ ends, indicating the respective genomic region (lines) and the *lacZ* reporter gene (green arrows). All of the positions indicated are relative to the translational start site (+1) of *cas1*. β‐galactosidase activity of a promoterless *lacZ* reporter and of the respective *lacZ* reporter fusions were measured in the WT. E) qRT‐PCR of *cas10* and *csm3* in the WT and respective P*cas* mutants. F) Phage titers were measured after infection with phage phiIPLA‐RODI on the bacterial lawns of *S. aureus* WT and mutant strains carrying the pCRISPR plasmid that expresses an additional crRNA for enhanced immunity. All the data shown above (A‐B, D‐F) are means ± standard deviation of three independent experiments. A two‐tailed unpaired Student's *t*‐test was used to calculate *P* values; ns, not significant, ^*^ *p* < 0.05, ^***^ *p* < 0.001.

Next, we examined the underlying inhibitory mechanism of QS on CRISPR‐Cas in *S. aureus*. Since the CRISPR promoter was not identified in WT, we first determined whether the promoter for the pre‐crRNA transcript was located in the leader sequence upstream of CRISPR1, as reported in *E. coli*.^[^ ^44^ ^]^ We performed RT‐PCR analysis using nine sets of primers to amplify regions spanning different gene combinations (Table S2, Supporting Information) to examine the potential co‐transcription of the CRISPR1 locus and *cas* genes (Figure 1B). Each targeted region showed a specific size of DNA fragment from both gDNA and cDNA templates, indicating that the CRISPR1 locus and *cas* genes were transcribed on a single polycistronic mRNA by a potential promoter located in the leader sequence of the CRISPR1 locus (termed P*crispr1*) (Figure S3A, Supporting Information). The P*crispr1* sequence was then cloned into a promoterless *lacZ* fusion vector to evaluate its role in transcription. We observed a 10‐fold increase in the expression of the P*crispr1*‐*lacZ* transcriptional fusion compared to the promoterless *lacZ* fusion, revealing that the P*crispr1* sequence might harbor a key element driving transcription (**Figure** **3**A). After constructing a ∆P*crispr1* mutant, we evaluated the importance of the P*crispr1* sequence in CRISPR‐Cas locus transcription by measuring *crispr1* and *cas* genes expression using qRT‐PCR. The expression of CRISPR1 pre‐crRNA was downregulated 13‐fold in the ∆P*crispr1* mutant compared to that in the WT strain (Figure 3B), indicating that the P*crispr1* sequence contained a promoter for CRISPR‐*cas* in the WT. RNA‐seq analysis in the ∆P*crispr1* mutant corroborated this result (Figure S3B, Supporting Information). However, compared to the dramatically decreased level of *crispr1*, the abundance of *cas10* and *csm3* transcripts was only modestly downregulated (≈1.5‐fold) in the ∆P*crispr1* mutant (Figure 3B), implying that there was potentially a second promoter regulating *cas* genes expression.

We quantified the abundance of *cas* transcripts in the WT strain using RNA‐seq to determine the predominant *cas* gene promoter. Within the *cas1* coding sequence, increased transcriptional activity was observed starting from the position of *cas1* +598 (Figure 3C). Further analysis showed that the position from +598 to +600 of *cas1* is ATG, indicating that there is a potential promoter upstream of position +597 in *cas1* to initiate the transcription of other *cas* genes (Figure 3C). To identify core promoters within this region, we constructed a series of transcriptional fusions carrying different 5′ end deletions and tested the constructs using the β‐galactosidase assay (Figure 3D). Since there is a 73 bp fragment between the CRISPR1 array and *cas1*, we also fused it into the *lacZ* reporter gene (Figure 3D). The promoterless *lacZ* and P*cas*‐*lacZ*‐73‐1 yielded minimal reporter activity. P*cas*‐*lacZ*+1+597 resulted in a 15‐fold increase in reporter activity compared to the promoterless *lacZ*. A further deletion with the remaining 300 bp at the 3′ end (P*cas*‐*lacZ*+298+597) yielded a similar reporter expression level to that of P*cas*‐*lacZ*+1+597, while P*cas*‐*lacZ*+448+597 showed a slight decrease in reporter activity relative to P*cas*‐*lacZ*+1+597 (Figure 3D). This suggests that a potential core promoter exists between positions +298 and +597 of *cas1*. To examine whether the putative promoters have an effect on *cas* genes transcription, we deleted the corresponding regions (Figure S3C, Supporting Information) and quantified *cas* genes transcripts using qRT‐PCR. Both *cas10* and *csm3* expression levels were reduced 5‐fold in the ∆P*cas*+1+597, ∆P*cas*+298+597, and ∆P*cas*+448+597 mutants compared with those in the WT strain, whereas their expression was not significantly affected in the ∆P*cas*‐73‐1 mutant (Figure 3E). Importantly, a strain lacking the 308 bp at the 3′‐end of *cas1* (∆*cas1*+598+905) had no effect on *cas* genes transcription, and complementation of the ∆P*cas*+1+597 mutant using a plasmid‐borne *cas1*+1+597 could not restore *cas10* and *csm3* expression to the WT level, further confirming that an active promoter is located between +1 and +597 of *cas1* (Figure S3C,D, Supporting Information).

To test the effect of putative promoters on CRISPR‐Cas activity, we carried out plaque assays. Because our previous study showed that the WT strain only has weak immunity against phage philPLA‐RODI infection in a plaque assay compared to the ∆CRISPR ∆*cas* mutant,^[^ ^21^ ^]^ we constructed a pCRISPR plasmid expressing a crRNA targeting phage philPLA‐RODI, in order to enhance the immunity of the WT strain. Specifically, we designed a plasmid to carry the leader sequence of the CRISPR array, two repeats interrupted with a spacer targeting the phage philPLA‐RODI *gp036* gene (Figure S3E, Supporting Information); and the recombinant plasmid was transformed into the WT, ∆CRISPR ∆*cas*, and promoter mutant strains. Transformants were used as recipients when infected with phage philPLA‐RODI. In accordance with our *cas* genes expression data, the EOP of phage philPLA‐RODI on the ∆P*cas*+1+597 and ∆P*cas*+298+597 mutant strains was decreased by more than 10^7^‐fold compared with WT (Figure 3F, Figure S3F, Supporting Information), whereas no difference in EOP was observed for ∆P*cas*‐73‐1 and ∆*cas1*+598+905 mutant strains compared with the WT (Figure 3F, Figure S3G,H, Supporting Information). Notably, the EOP on the ∆P*cas*+448+597 mutant was 10^3^‐fold higher than that on the ∆P*cas*+298+597 mutant (Figure 3F, Figure S3F, Supporting Information), which was consistent with the weak reporter activity detected in the β‐galactosidase assay. These data demonstrate that an active promoter located between positions +298 and +597 of *cas1* (termed P*cas*) drives transcription of *cas* genes downstream of *cas1*.

To determine whether AgrA directly interacts with the promoter regions, we examined the interaction of AgrA with the CRISPR‐Cas P*crispr1* and P*cas* sequences using EMSAs. As shown in Figure S3I (Supporting Information), AgrA‐His_6_ protein shifted the P2 promoter of the *agr* locus, starting at a concentration of 0.25 µm, as expected,^[^ ^45^ ^]^ confirming that the tagged protein was functionally active. However, the P*crispr1* and P*cas* DNA fragments did not shift in the presence of AgrA‐His_6_, even at a concentration of 2.5 µm, indicating that AgrA could not specifically bind to these promoter regions (Figure S3I, Supporting Information). These results indicate that AgrA indirectly represses the expression of *cas* genes and that additional regulatory factors downstream of AgrA may underlie the QS‐mediated repression of *cas* genes.

### 2.5 SarA and ArcR Directly Activate Type III‐A CRISPR‐Cas Expression and Interference

To identify the direct transcriptional regulators of the type III‐A CRISPR‐Cas system, we applied a DNA pull‐down assay using the P*crispr1* and P*cas* sequences as bait to capture binding proteins from WT cellular lysates. Mass spectrometry analysis showed that 17 and 50 proteins had potential interactions with the P*crispr1* and P*cas* sequences, respectively (Tables S3 and S4, Supporting Information). Among the 17 candidate proteins interacting with the P*crispr1* sequence, six were predicted to be transcriptional regulators. Additionally, 20 out of 50 proteins captured with the P*cas* sequence were transcriptional regulators. Consistent with our findings that AgrA did not bind directly to the P*crispr1* and P*cas* promoters (Figure S3F, Supporting Information), AgrA was not captured in the DNA pull‐down assays, further confirming that the AgrA‐mediated control of CRISPR‐*cas* expression is indirect. In addition, four transcriptional regulators, SarA, SarR, ArcR, and Rot, were captured by both the P*crispr1* and P*cas* sequences in the DNA pull‐down assays (Tables S3 and S4, Supporting Information).

**Figure 4:**
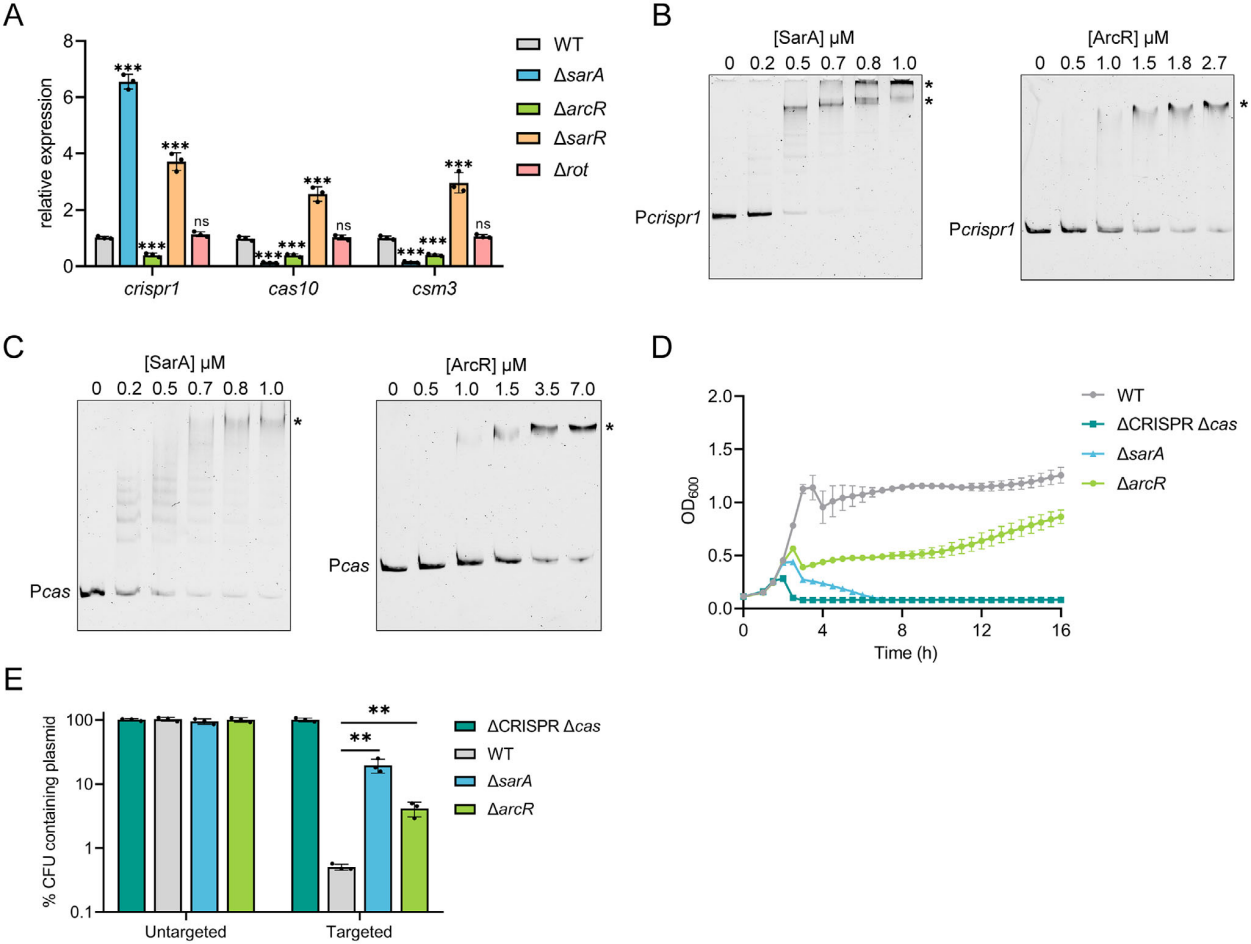
SarA and ArcR directly regulate the expression of the type III‐A CRISPR‐Cas system. A) qRT‐PCR analysis of *crispr1*, *cas10*, and *csm3* expression in the WT and the individual mutant strains infected by phage phiIPLA‐RODI after 10 h of growth in liquid culture, respectively. B and C) EMSAs were performed to analyze SarA and ArcR binding to the P*crispr1* sequence (B) or P*cas* sequence (C). FAM‐5′‐end‐labelled P*crispr1* sequence (B) or P*cas* sequence (C) was incubated with the increasing concentrations of purified SarA‐His_6_ and ArcR‐His_6_ proteins. DNA‐protein complexes are indicated by an asterisk. Competitive EMSAs. D) Growth curve of the WT, ∆CRISPR ∆*cas*, ∆*sarA*, and ∆*arcR* strains infected with phage phiIPLA‐RODI at an MOI of 0.01. E) Retention of the untargeted plasmid pRMC2 or targeted plasmid pCR1SP1 in the WT, ∆CRISPR ∆*cas*, ∆*sarA*, and ∆*arcR* strains. The strains containing the plasmid were grown in TSB with ATc for 10 h followed by plating on TSA with or without chloramphenicol. % CFUs containing plasmid were scored as the number of Cm^R^ CFU/the total number of CFU. All the data shown above (A, D and E) are means ± standard deviation of three independent experiments. A two‐tailed unpaired Student's *t*‐test was used to calculate *P* values; ns, not significant, ^**^ *p* < 0.005, ^***^ *p* < 0.001.

To examine whether these four transcriptional regulators are involved in the transcription of both *crispr* and *cas* genes, we measured the expression levels of *crispr1*, *cas10*, and *csm3* in four individual mutants (∆*sarA*, ∆*sarR*, ∆*arcR*, and ∆*rot*) either with or without phage philPLA‐RODI infection at HCD using qRT‐PCR. After treatment with phage infection, a marked increase in the expression of *crispr1* was observed in the ∆*sarA* mutant, whereas the expression of *cas* genes was decreased by 8‐fold in comparison with the WT (**Figure** **4**A). We observed increased expression of *crispr1* and *cas* genes in the ∆*sarR* mutant relative to the WT, but decreased expression in the ∆*arcR* mutant (Figure 4A). Furthermore, deletion of *rot* had no significant effect on the expression of *crispr1* and *cas* genes (Figure 4A). In agreement with these results, we observed similar *crispr1* and *cas* gene expression profiles in these mutants in the absence of phage infection (Figure S4A, Supporting Information).

Since SarA, SarR, and ArcR affected the expression of *crispr 1* and *cas* genes, we performed EMSAs to test whether these regulators could directly bind to the P*crispr1* and P*cas* sequences, respectively. None of the three proteins shifted the promoter sequence of *gmk*, which served as a negative control (Figure S4B, Supporting Information). As shown in Figure 4B,C, both SarA‐His_6_ and ArcR‐His_6_ proteins specifically shifted the P*crispr1* and P*cas* sequences, but SarR‐His_6_ did not (Figure S4C, Supporting Information). The specific binding of SarA‐His_6_ and ArcR‐His_6_ to the P*crispr1* and P*cas* sequences was confirmed by competitive EMSAs (Figure S4D,E, Supporting Information). Collectively, these results demonstrate that SarA binds to the P*cas* promoter and activates *cas* genes expression but represses the transcription of CRISPR1 by interacting with the P*crispr1* sequence, and that ArcR functions as a direct positive regulator of both promoters.

Next, we determined whether SarA‐ and ArcR‐mediated direct activation of *cas* genes altered CRISPR‐Cas interference activity. We exposed the mutant strains to phage phiIPLA‐RODI, and monitored the bacterial growth over time in liquid culture. Compared to the WT strain, cells lacking the *sarA* gene succumbed to phage phiIPLA‐RODI infection, similar to the ∆CRISPR ∆*cas* mutant (Figure 4D). However, the ∆*arcR* mutant exhibited a slight reduction in the immunity level relative to the WT (Figure 4D). Similar trends in antiplasmid immunity were detected in ∆*sarA* and ∆*arcR* mutants as observed for antiphage immunity. Specifically, compared with the retention of the pCR1SP1 plasmid in the WT, we observed a 40‐fold and 8‐fold increase in plasmid retention in the ∆*sarA* and ∆*arcR* mutants, respectively (Figure 4E). Taken together, these data demonstrate that SarA and ArcR promote the type III‐A CRISPR‐Cas expression by binding to the P*cas* promoter, and thus triggering the activation of its interference activity in *S. aureus*.

### 2.6 The Binding Sites for SarA and ArcR in P*crispr1* and P*cas* Sequence

**Figure 5:**
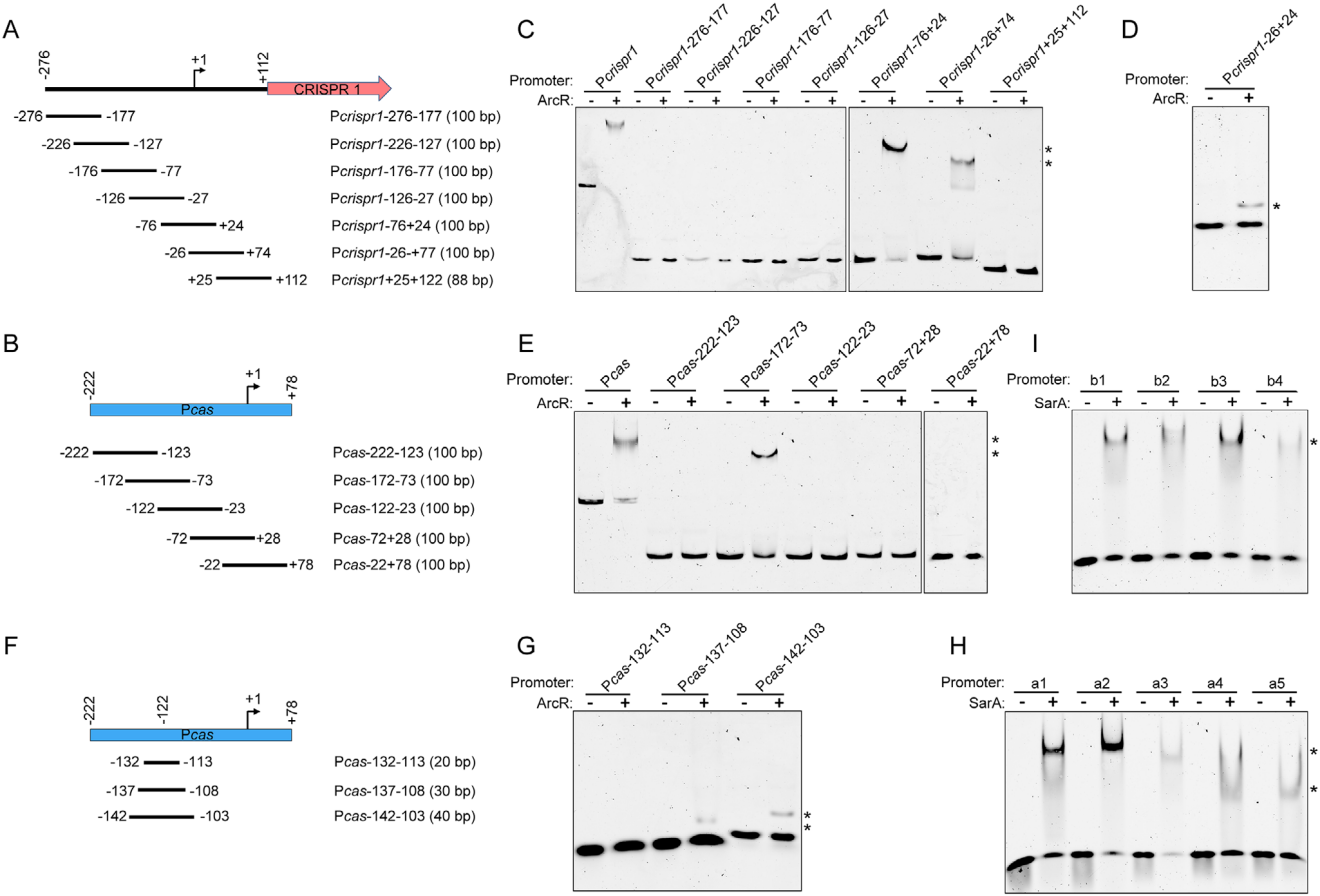
Binding sites for SarA and ArcR in the P*crispr1* and P*cas* sequences. Schematic representation of P*crispr1* A) and P*cas* B) loci. Various ≈50 bp overlapping DNA fragments covering different regions of P*crispr1* and P*cas* were tested in EMSA experiments. The transcriptional start sites (+1) of P*crispr1* and P*cas* are indicated by bent arrows. All positions indicated are relative to the transcriptional start site. EMSAs. C) Seven truncated FAM‐5′‐end‐labelled P*crispr1* fragments were incubated with ArcR‐His_6_ at a concentration of 7.2 µm. D) FAM‐5′‐end‐labelled P*crispr1*‐26+24 fragment was incubated with 7.2 µm ArcR‐His_6_. E) Five truncated FAM‐5′‐end‐labelled P*cas* fragments were incubated with 7.2 µm ArcR‐His_6_. F) Schematic representation of three DNA fragments flanking position ‐122 of P*cas*. G) Three FAM‐5′‐end‐labelled DNA fragments were incubated with ArcR‐His_6_ at a concentration of 7.2 µm, respectively. The FAM‐5′‐end‐labelled putative SarA‐binding sites on P*crispr1* (a1‐a5) H) and P*cas* (b1‐b4) I) fragments were incubated with SarA‐His_6_ at a concentration of 1.0 µm, respectively. DNA‐protein complexes are indicated by asterisks. +, with protein; –, without protein.

To further determine the binding sites of SarA and ArcR in P*crispr1* and P*cas* sequences, we divided the P*crispr1* and P*cas* sequences into seven and five ≈100 bp DNA fragments, respectively. The truncated DNA fragments were named according to the 5′ and 3′ positions of the P*crispr1* and P*cas* with respect to their transcriptional start site. There was a 50 bp overlap between these consecutive DNA fragments (**Figure** **5**A,B). The binding between the DNA fragments and purified SarA‐His_6_ or ArcR‐His_6_ proteins was analyzed using EMSAs. We observed that ArcR‐His_6_ specifically shifted the DNA fragments P*crispr1*‐76+24 and P*crispr1*‐26+74, but did not shift the DNA fragments P*crispr1*‐276‐177, P*crispr1*‐266‐127, P*crispr1*‐176‐77, P*crispr1*‐126‐27, and P*crispr1*+25+112 (Figure 5C), indicating that the binding site for ArcR was located in P*crispr1*‐26 and P*crispr1*+24. Binding of ArcR‐His_6_ to the fragment P*crispr1*‐26+24 was then confirmed by EMSAs (Figure 5D). ArcR‐His_6_ shifted the DNA fragment P*cas*‐172‐73, but not the other truncated P*cas* sequences (Figure 5E). The non‐binding of ArcR‐His_6_ to the DNA fragment P*cas*‐222‐123 and P*cas*‐122‐23 indicated that the ArcR‐binding site in P*cas* sequence was close to position P*cas*‐122. Therefore, we generated three DNA fragments, including position P*cas*‐122 for EMSAs (Figure 5F). The results showed that both P*cas‐*137‐108 and P*cas*‐142‐103 interacted with ArcR‐His_6_, but not P*cas*‐132‐113 (Figure 5G). Previous studies revealed that ArcR in *S. aureus* was predicted to be a CRP family transcriptional regulator,^[^ ^41^ ^]^ and the conserved DNA binding motif of CRP is TGTGA‐N_6_‐TCACA in *E. coli*.^[^ ^46^ ^]^ Therefore, we examined whether the P*crispr1*‐26+24 and P*cas‐*137‐108 fragments contain a conserved DNA‐binding motif for ArcR. The homology analysis showed that the putative ArcR binding site is located between positions +4 and +20 (5′‐**TGTCA**AAAAAAG**TGACA**‐3′) in the P*crispr1*‐26+24 sequence and between positions ‐133 and ‐118 (5′‐**TGTGA**AGTTAT**TAGTA**‐3′) in the P*cas‐*137‐108 sequence. However, the EMSAs results showed that ArcR‐His_6_ could not shift these two putative binding sites (Figure S5A, Supporting Information), implying that the nucleotides flanking the putative binding site were also necessary for ArcR binding. Our results indicate that ArcR can specifically bind to P*crispr1*‐26+24 and P*cas‐*137‐108 sequences (Figure S5B,C, Supporting Information).

Moreover, EMSAs showed that the SarA‐His_6_ could bind to each truncated P*crispr1* and P*cas* sequence (Figure S5D, E), implying that multiple SarA‐binding sites were present in the P*crispr1* and P*cas* sequences. SarA has been reported to have a preference for AT‐rich binding sites,^[^ ^47^, ^48^ ^]^ and the consensus SarA‐binding motif has been predicted to be ATTTTAT.^[^ ^47^, ^49^ ^]^ In agreement, we identified five and four putative SarA‐binding sites in the P*crispr1* (a1‐a5) and P*cas* (b1‐b4) sequences, respectively (Figure S5B,C, Supporting Information). EMSAs confirmed that SarA‐His_6_ could bind to a1‐a5 and b1‐b4 binding sites (Figure 5H,I). Notably, we found that the a4 and b2 SarA‐binding sites in the P*crispr1* and P*cas* sequences were located close to ArcR‐binding sites (Figure S5B,C, Supporting Information), indicating that SarA and ArcR might compete for interactions with the P*crispr1* and P*cas* sequences.

### 2.7 SarA and ArcR Compete for Binding to the P*crispr1* and P*cas* Sequence

**Figure 6:**
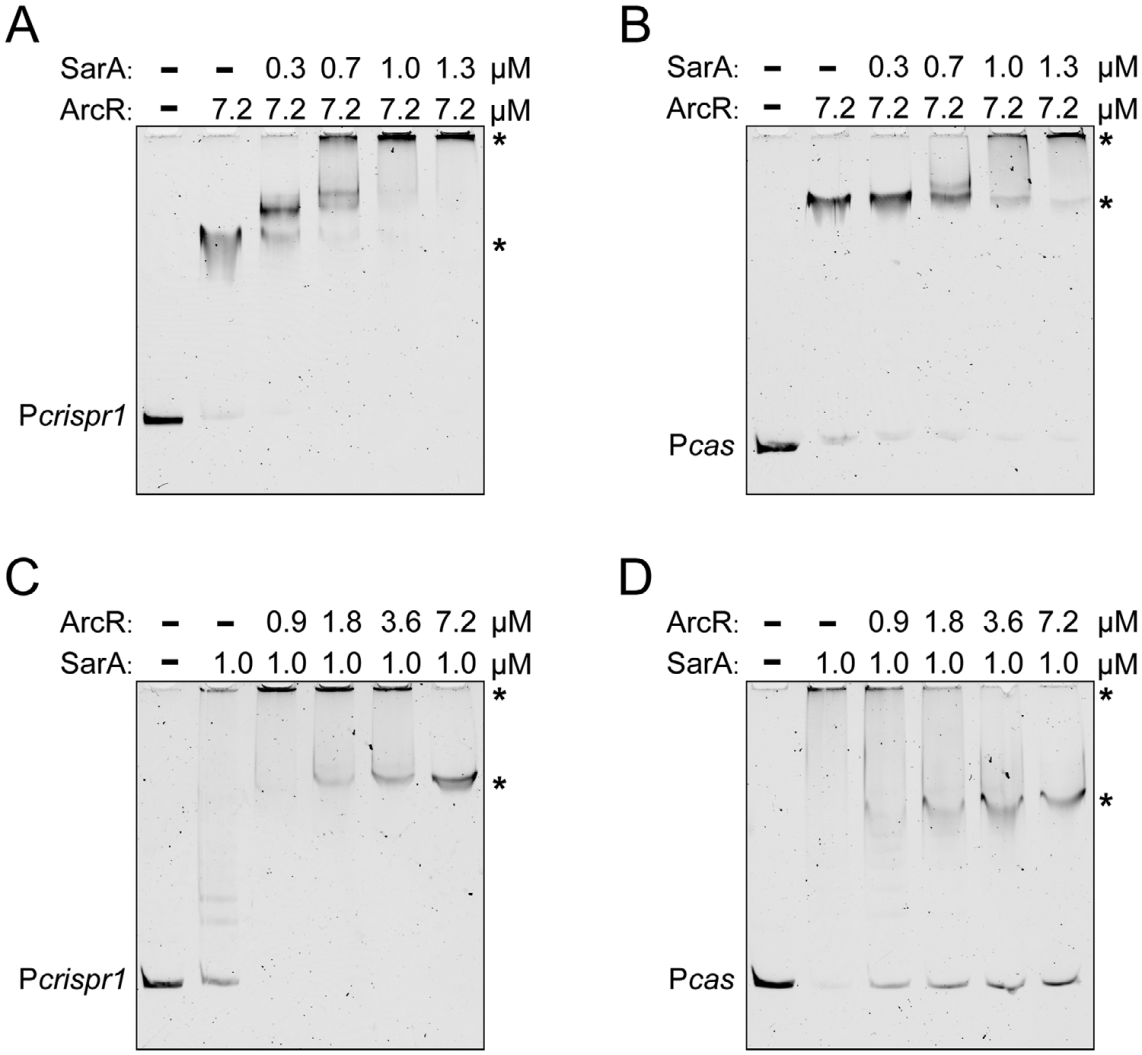
Competition between SarA and ArcR binding to P*crispr1* and P*cas* sequences using competitive EMSAs. FAM‐5′‐end‐labelled P*crispr1* sequence A) or P*cas* sequence B) was first incubated with 7.2 µm purified ArcR‐His_6_ followed by the addition of increasing concentrations of SarA‐His_6_ (from 0.3 to 1.3 µm). FAM‐5′‐end‐labelled P*crispr1* sequence C) or P*cas* sequence D) was first incubated with 1.0 µm purified SarA‐His_6_ followed by the addition of increasing concentrations of ArcR‐His_6_ (from 0.9 to 7.2 µm). The shifted bands are indicated by asterisks.

Our above results showed that SarA and ArcR binding sites were located close to each other, indicating a potential competition between SarA and ArcR. Thus, we performed competitive EMSAs to investigate if SarA and ArcR could compete for binding to the P*crispr1* and P*cas* sequences. The P*crispr1* or P*cas* fragment was preincubated with ArcR‐His_6_ at room temperature for 30 min, followed by adding increasing concentrations of SarA‐His_6_. As shown in **Figure** **6**A,B, the increasing concentrations of SarA‐His_6_ shifted the P*crispr1*‐ArcR‐His_6_ or P*cas*‐ArcR‐His_6_ complex to a slower‐migrating complex similar to that formed by SarA‐His_6_ alone, suggesting that SarA‐His_6_ could displace ArcR‐His_6_ from the P*crispr1* and P*cas* sequences. A similar assay was performed by first incubating the P*crispr1* and P*cas* fragments with SarA‐His_6_, followed by adding increasing concentrations of ArcR‐His_6_. The results showed that ArcR‐His_6_ could also displace SarA‐His_6_ from the P*crispr1* and P*cas* sequences. However, this displacement of SarA‐His_6_ required a much higher ArcR‐His_6_ concentration of > 3.6 µm (Figure 6C,D). Together, our data demonstrates that SarA is more competitive than ArcR in terms of interaction with P*crispr1* and P*cas* sequences. Considering that the P*cas* contained multiple SarA‐binding sites, we further analyzed the effect of SarA on P*cas* transcription by performing RNA‐seq. Our RNA‐seq data indicated a decreased abundance of P*cas* transcripts in the ∆*sarA* mutant compared to the WT, suggesting that SarA positively regulated the transcription of P*cas* (Figure S4F, Supporting Information). Combined with the results showing that ∆*sarA* is susceptible to phage phiIPLA‐RODI infection, similar to the ∆CRISPR ∆*cas* mutant, while ∆*arcR* was still able to grow under phage infection (Figure 4D), this demonstrates that SarA plays a predominant role in regulating CRISPR‐Cas system expression.

### 2.8 AgrA Represses type III‐A CRISPR‐Cas Expression and Interference Through Downregulation of SarA and ArcR Regulators

**Figure 7:**
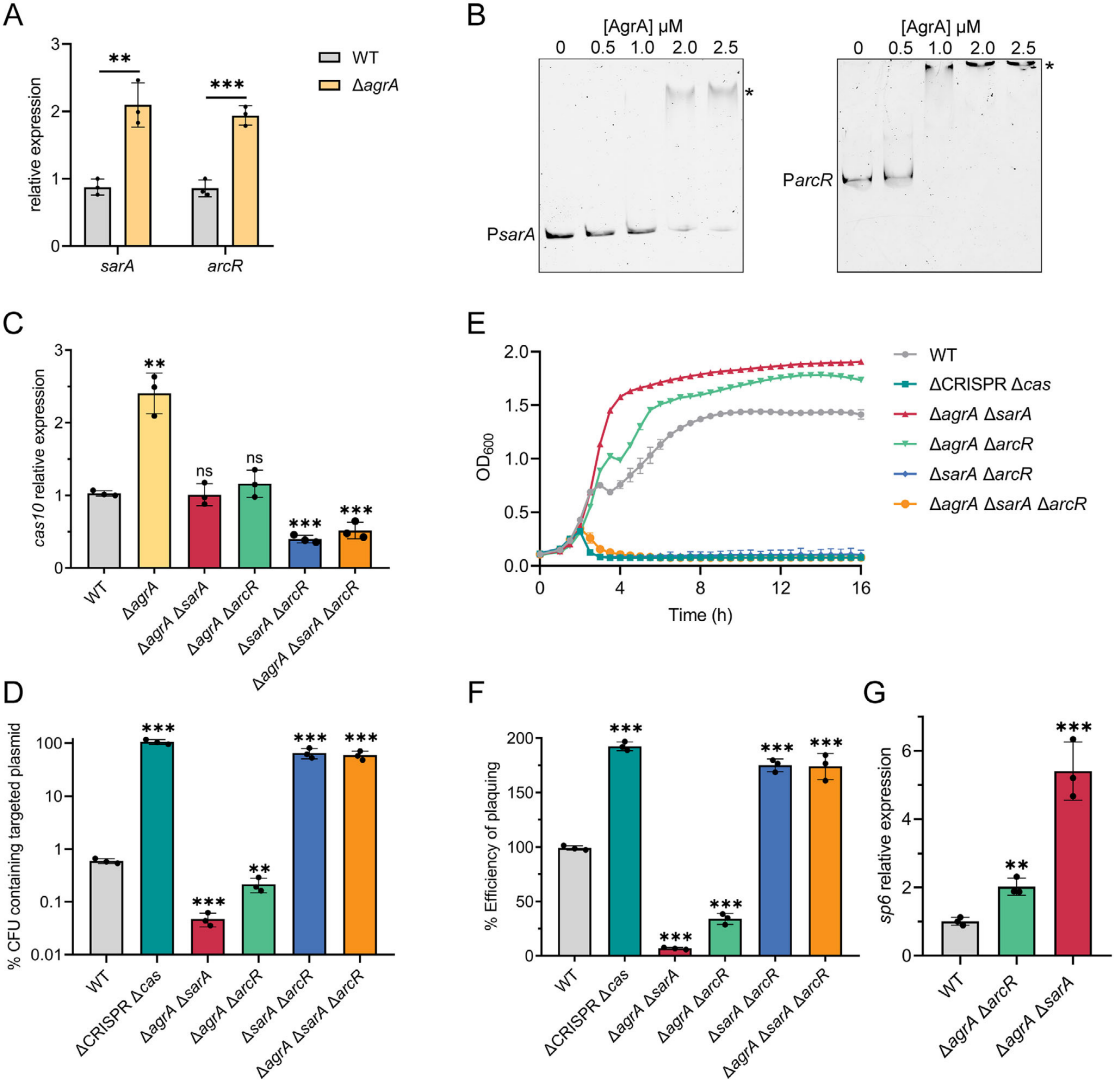
The AgrA‐mediated regulation of CRISPR‐Cas is dependent on SarA and ArcR. A) qRT‐PCR measurement of *sarA* and *arcR* expression in the WT and ∆*agrA* mutant after 10 h of growth in liquid culture, respectively. B) FAM‐5′‐end‐labelled P*sarA* sequence and P*arcR* sequence were incubated with the increasing concentrations of purified AgrA‐His_6_ protein. Acetyl phosphate (50 mM) was added to all EMSAs. DNA‐protein complexes are indicated by an asterisk. C) qRT‐PCR measurement of *cas10* expression in the WT, ∆*agrA*, ∆*sarA*, ∆*arcR*, and double mutants (∆*agrA* ∆*sarA*, ∆*agrA* ∆*arcR*, ∆*sarA* ∆*arcR*), and triple mutant ∆*agrA* ∆*sarA* ∆*arcR* of the TZ0912 strain after 10 h of growth in liquid culture, respectively. D) Retention of the targeted plasmid pCR1SP1 in the WT, ∆CRISPR ∆*cas*, and double mutants (∆*agrA* ∆*sarA*, ∆*agrA* ∆*arcR*, ∆*sarA* ∆*arcR*), and triple mutant ∆*agrA* ∆*sarA* ∆*arcR*. The strains containing the pCR1SP1 plasmid were grown in TSB with ATc for 10 h followed by plating on TSA with or without chloramphenicol. % CFUs represents the ratio of bacteria grown on plates containing chloramphenicol to the number of bacteria growing on plates without antibiotics. E) Growth curve of the WT, ∆CRISPR ∆*cas*, and double mutants (∆*agrA* ∆*sarA*, ∆*agrA* ∆*arcR*, ∆*sarA* ∆*arcR*), and triple mutant ∆*agrA* ∆*sarA* ∆*arcR* infected with phage phiIPLA‐RODI at an MOI of 0.01. F) The immunity against phage phiIPLA‐RODI of relevant strains was represented as efficiency of plaquing, which is a ratio relative to the number of plaques measured on the WT. G) qRT‐PCR measurement of *spc6* expression in the WT, and double mutants (∆*agrA* ∆*sarA*, ∆*agrA* ∆*arcR*) after 10 h of growth in liquid culture, respectively. All the data shown above (A, C–G) are means ± standard deviation of three independent experiments. A two‐tailed unpaired Student's *t*‐test was used to calculate *P* values; ns, not significant, ^**^ *p* < 0.005, ^***^ *p* < 0.001.

Interestingly, a previous microarray study of the *agr* system showed that the *arcR* and *sarA* were downregulated by *agr* in a HA‐MRSA strain.^[^ ^50^ ^]^ Our qRT‐PCR analysis confirmed this observation. As shown in **Figure** **7**A, both *sarA* and *arcR* were upregulated in the ∆*agrA* mutant compared to the WT at HCD. EMSAs further confirmed that AgrA directly repressed the expression of *sarA* and *arcR* by binding to their promoters. (Figure 7B, Figure S6A, Supporting Information). Therefore, we hypothesized that AgrA may repress type III‐A CRISPR‐Cas expression by inhibiting SarA‐ and ArcR‐mediated activation of *cas* genes. To confirm our hypothesis, we constructed the double mutants ∆*agrA* ∆*sarA*, ∆*agrA* ∆*arcR*, and ∆*sarA* ∆*arcR*, as well as a triple mutant ∆*agrA* ∆*sarA* ∆*arcR*, and quantified the expression of *cas10* by qRT‐PCR. Deletion of *arcR* or *sarA* restored *cas10* expression in the ∆*agrA* mutant to the WT level (Figure 7C). Importantly, the level of *cas10* expression was equivalent between the ∆*sarA* ∆*arcR* mutant and the ∆*agrA* ∆*sarA* ∆*arcR* mutant (Figure 7C), indicating that AgrA represses *cas* genes expression in a SarA/ArcR‐dependent manner. These results suggest that when the bacteria grow to HCD, AgrA represses CRISPR‐Cas expression via inhibiting the activation of *cas* genes by SarA and ArcR. We next wanted to investigate whether the regulation of P*sarA* and P*arcR* by AgrA is essential for controlling CRISPR‐Cas expression. To this end, we constructed two sets of complementation strains for *sarA* and *arcR* in the ∆*sarA* ∆*arcR* background using a low‐copy‐number plasmid pLI50. In one set, *sarA* and *arcR* were reintroduced under the control of their native promoters (P*native*‐*sarA arcR*) (Figure S6B, Supporting Information). In the other set, both genes were driven by the P*gmk* promoter, a constitutive promoter that is not regulated by AgrA (P*gmk*‐*sarA arcR*) (Figure S6B, Supporting Information). We introduced the P*native‐sarA arcR* and P*gmk‐sarA arcR* plasmids into *∆agrA ∆sarA ∆arcR* and *∆sarA ∆arcR* mutants, generating TZ0912‐1 (*∆sarA ∆arcR*::P*native*‐*sarA arcR*), TZ0912‐2 (*∆sarA ∆arcR*::P*gmk‐sarA arcR*), TZ0912‐3 (*∆agrA ∆sarA ∆arcR*::P*native‐sarA arcR*), and TZ0912‐4 (*∆agrA ∆sarA ∆arcR*::P*gmk‐sarA arcR*) strains, and analyzed *cas10* expression. As shown in Figure S6C (Supporting Information), we observed AgrA‐mediated repression of *cas10* in the TZ0912‐1 strain. In contrast, *cas10* expression was significantly increased in the TZ0912‐3 strain, where AgrA is absent and thus unable to repress *sarA* and *arcR* expression. Additionally, no significant difference in *cas10* expression was observed between TZ0912‐2 and TZ0912‐4 strains (Figure S6C, Supporting Information). These results further support our hypothesis that AgrA represses the CRISPR‐Cas system by downregulating *sarA* and *arcR*, and that AgrA‐mediated *cas* genes repression depends on the binding of AgrA to P*sarA* and P*arcR*. To assess whether these findings are specific to the TZ0912 strain, we selected an additional *S. aureus* 08BA02176 strain, which carries a type III‐A CRISPR‐Cas system and was isolated from pig farmers in Canada.^[^ ^51^ ^]^ We generated multiple deletion mutants of this strain, including ∆*agrA*, ∆*sarA*, ∆*arcR*, ∆*agrA* ∆*sarA*, ∆*agrA* ∆*arcR*, ∆*sarA* ∆*arcR*, and ∆*agrA* ∆*sarA* ∆*arcR* mutants. Subsequently, qRT‐PCR analysis was performed and showed a similar *cas* gene expression pattern to that observed in the TZ0912 strain (Figure S6D, Supporting Information). These findings suggest that the regulatory mechanism we revealed is not restricted to the TZ0912 strain but may represent a conserved feature in *S. aureus*.

The DNA cleavage activity of Cas10 is required for the type III‐A CRISPR‐Cas immunity against phage infection.^[^ ^13^ ^]^ Therefore, we examined the effect of altered *cas10* expression on CRISPR‐Cas‐mediated immunity against the targeted plasmids and phages. Interference against plasmids was assessed by measuring the retention of the targeted plasmid pCR1SP1 and the untargeted plasmid in the WT and mutant strains. As expected, none of the strains lost the untargeted plasmid (Figure S6E, Supporting Information). Compared with the retention of the pCR1SP1 plasmid in the WT, the deletion of both *sarA* and *arcR* in the ∆*agrA* mutant abolished type III‐A against the targeted plasmid, which was also observed in the ∆*sarA* ∆*arcR* mutant (Figure 7D), indicating that AgrA‐mediated repression was dependent on SarA and ArcR. Besides, a decrease in pCRISP1 retention was observed in the ∆*agrA* ∆*sarA*, and ∆*agrA* ∆*arcR* mutants, indicating that both activated ArcR and SarA could promote CRISPR‐Cas expression and interference activity (Figure 7D).

Moreover, we exposed the mutant strains to phiIPLA‐RODI and monitored the bacterial growth over time in liquid culture. Compared to the WT strain, the ∆*sarA* ∆*arcR* and ∆*agrA* ∆*sarA* ∆*arcR* mutants failed to provide immunity (Figure 7E), consistent with the reduced *cas10* expression. Although *cas10* expression in the ∆*agrA* ∆*sarA*, and ∆*agrA* ∆*arcR* mutants was restored to the level of the WT, these two mutants showed more effective antiphage immunity than the WT (Figure 7E). A similar result was obtained when we measured the EOP of phiIPLA‐RODI on these mutant strains (Figure 7F). We have previously demonstrated that the expression level of crRNA is also closely related with CRISPR‐Cas immune efficiency in the WT,^[^ ^21^ ^]^ which has also been shown in *Streptococcus pyogenes* (*S. pyogenes*) type II‐A CRISPR‐Cas system.^[^ ^52^ ^]^ Therefore, we analyzed the transcriptional level of *sp6* in the WT, ∆*agrA* ∆*arcR*, and ∆*agrA* ∆*sarA* strains and found increased *sp6* expression levels in both mutant strains compared to the WT strain (Figure 7G), indicating that the increased antiphage immunity against phiIPLA‐RODI in both mutant strains was due to higher expression of *sp6* than that in the WT strain. These results demonstrate that AgrA can repress the type III‐A CRISPR‐Cas interference in *S. aureus* by inhibiting both SarA and ArcR, which are positive regulators of the type III‐A CRISPR‐Cas system.

### 2.9 P*crispr1* and P*cas* Sequences are Conserved in Staphylococcal type III‐A CRISPR‐Cas System

**Figure 8:**
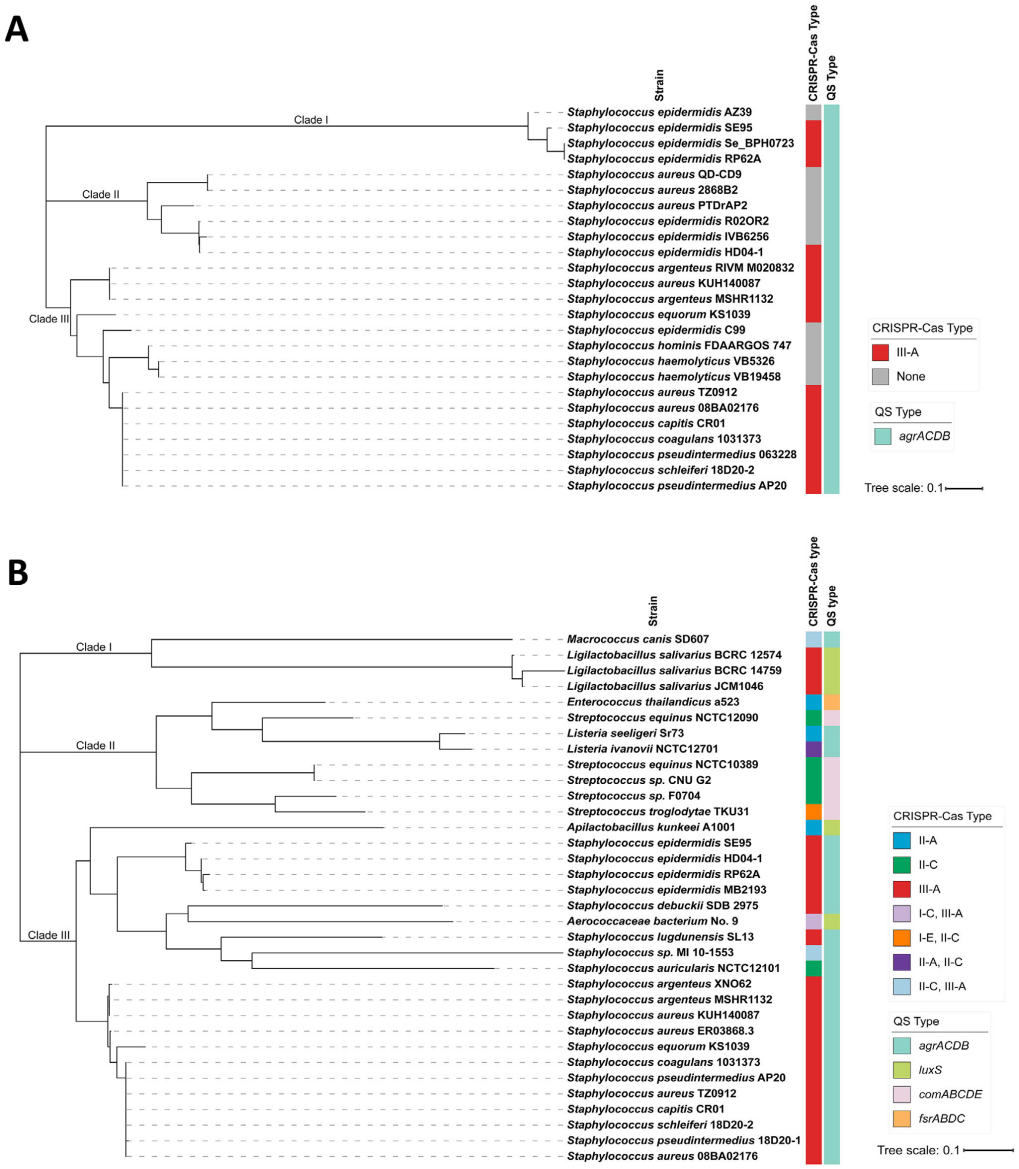
Distribution of P*crispr1* and P*cas* sequences in Firmicutes. Phylogenetic trees were constructed based on P*crispr1* A) and P*cas* B) homologous sequences. More related P*crispr1* and P*cas* homologs were also identified in Firmicutes that are not present in trees, and are reported in Tables S5 and S6 (Supporting Information), respectively.

To investigate whether the identified P*crispr1* and P*cas* sequence is conserved in Firmicutes, we performed BLAST analysis against the NCBI nr nucleotide database limited to Firmicutes, using the identified P*crispr1* and P*cas* sequence as queries. A comprehensive list of P*crispr1* and P*cas* homologs was listed in Tables S5 and S6 (Supporting Information), respectively. P*crispr1* homologs are mainly distributed in *Staphylococcus* of Firmicutes (Table S5, Supporting Information). Maximum likelihood phylogenetic analysis of the P*crispr1* homologs revealed that most homologs are highly conserved in *Staphylococci* carrying the type III‐A CRISPR‐Cas system (**Figure** **8**A). In comparison, the P*cas* sequence is widespread in various Firmicutes, including *Staphylococcaceae*, *Listeriaceae*, *Aerococcaceae*, *Lactobacillaceae*, *Enterococcaceae*, and *Streptococcaceae* family (Table S6, Supporting Information). The phylogenetic tree of the P*cas* homologs was divided into three clades, which correspond to the related bacterial families (Figure 8B). The high conservation of P*cas* sequences in the type II and III CRISPR‐Cas systems in Firmicutes indicated that P*cas* was a conserved promoter of *cas* genes in both CRISPR‐Cas systems (Figure 8B). All P*cas* sequence from the staphylococcal type III‐A CRISPR‐Cas system was conserved, which clustered together in the phylogenetic tree. Meanwhile, the occurrence of the QS system was also examined in the P*crispr1*‐ and P*cas*‐positive strains. We found that all identified P*crispr1*‐ and P*cas*‐positive strains encoded a QS system (Figure 8A,B). Interestingly, the *comABCDE* operon in *Streptococcus* and *fsrABDC* operon in *Enterococcus* encode a *S. aureus agr*‐like two‐component regulatory system,^[^ ^53^, ^54^ ^]^ suggesting that *agr*‐mediated repression of the CRISPR‐Cas system may be a common phenomenon in Firmicutes. Additionally, we performed BLAST analysis using representative SarA and ArcR protein sequences against publicly available genomes of P*cas*‐positive strains. SarA was found to be widely distributed and highly conserved across all *Staphylococcus* species analyzed, with amino acid sequence identities exceeding 70% among different strains (Table S7, Supporting Information). ArcR was also conserved in most species, except for its absence in *S. lugdunensis*, *S. equorum*, and *S. debuckii* (Table S7, Supporting Information). We further assessed the conservation of identified SarA‐ and ArcR‐binding sites on P*cas* across P*cas*‐positive strains, and found that nearly all the strains (98.3%, 59/60) harbor these binding sites (Table S7, Supporting Information). Taken together, the widespread distribution and sequence conservation of both regulators and their binding sites on P*cas* suggest that the regulatory mechanism involving SarA and ArcR is likely conserved across diverse *Staphylococcus* species.

## 3 Discussion

**Figure 9:**
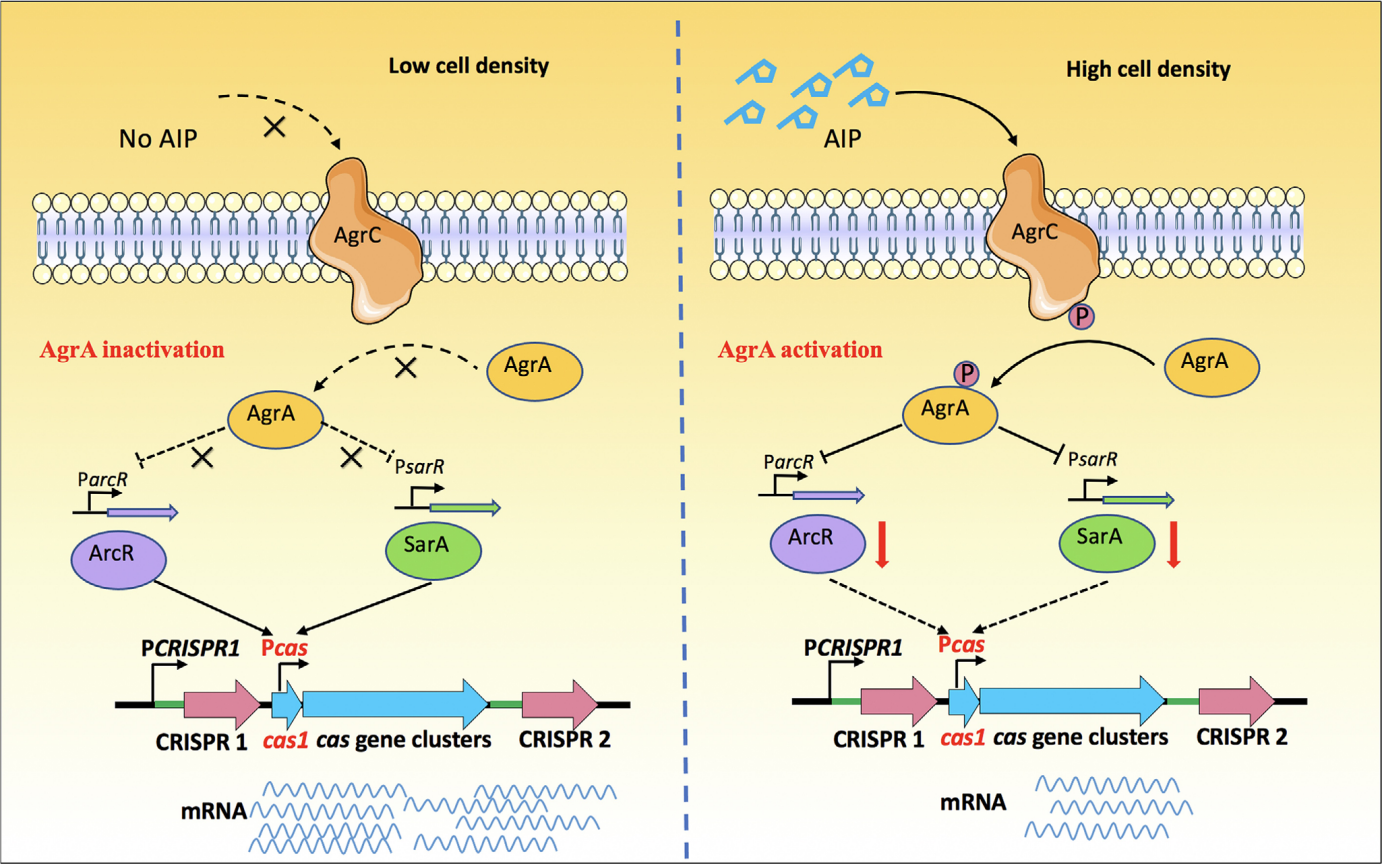
Model of QS‐mediated regulation of type III‐A CRISPR‐Cas system in *S. aureus*. At low cell density, no AIPs are sensed by AgrC, preventing AgrC from phosphorylating and activating AgrA. SarA and ArcR activate *cas* genes expression by binding to the P*cas* sequence. However, when the bacteria grow to high cell density, the AIPs concentration reaches a threshold, activating the kinase domain of AgrC and enabling autophosphorylation. AgrC then phosphorylates and activates AgrA. The activated AgrA subsequently represses the expression of SarA and ArcR, thereby inhibiting the direct activation of *cas* genes by SarA and ArcR.

Although the structure and immune mechanism of the type III‐A CRISPR‐Cas systems in *Staphylococci* have been extensively studied, the regulatory pathways controlling the expression of this system are underexplored. In the present study, we reveal that the QS regulator AgrA indirectly represses the expression and interference of the type III‐A CRISPR‐Cas system in *S. aureus*, which is contrary to previous observations in Gram‐negative bacteria.^[^ ^25^, ^26^, ^27^, ^28^, ^29^, ^30^ ^]^ We identified the promoters P*crispr1* and P*cas* that drove the transcription of crRNA and *cas* genes, respectively. These two promoters of the type III‐A CRISPR‐Cas system were used as baits to screen proteins that could interact with these elements. We found that the transcriptional regulators SarA and ArcR could directly bind to the P*cas* sequence, thereby enhancing the transcription of the CRISPR‐Cas system. Importantly, we confirmed that AgrA represses type III‐A CRISPR‐Cas activity via inhibiting the two direct regulators, SarA and ArcR at high cell density (**Figure** **9**). Our discovery that QS negatively regulates the type III‐A CRISPR‐Cas system in *S. aureus* suggests that this bacterium may benefit from acquiring antibiotic resistance or virulence genes carried on plasmids or prophages, specifically at high cell density.

QS is a universal phenomenon observed across and between Gram‐negative and Gram‐positive bacteria, and it allows bacteria to coordinate biological functions in response to cell density and microbial composition.^[^ ^55^ ^]^ Although the basic principle of QS is the same in all bacteria, QS can have profoundly different impacts on target genes expression in various bacterial species. For instance, Gram‐negative bacteria use their QS system to promote biofilm formation, and repress phage adsorption by downregulating the expression of phage receptor genes.^[^ ^31^, ^32^, ^56^ ^]^ In contrast, the QS *agr* system of *S. aureus* has the opposite effect on biofilm formation and phage adsorption.^[^ ^33^, ^57^ ^]^ Similarly, the QS‐mediated activation of the expression, activity, and adaptation of CRISPR‐Cas systems has been demonstrated in Gram‐negative bacteria *P. aeruginosa* and *Serratia*.^[^ ^25^, ^26^ ^]^ However, here we observed the opposite role of QS in regulating the *S. aureus* type III‐A CRISPR‐Cas system. This provides a new example of QS playing opposite roles in Gram‐positive and Gram‐negative bacteria. Our study highlights the diversity and complexity of QS regulation across different bacterial species. In response to changes in the population density, bacteria typically utilize QS system to control the expression of target genes to meet their needs. In *S. aureus*, numerous putative and proven resistance and virulence genes are located on mobile genetic elements (MGEs), including plasmids, prophages, transposons, insertion sequences, pathogenicity islands, and staphylococcal cassette chromosomes,^[^ ^58^ ^]^ which constitute ≈25% of the staphylococcal genome.^[^ ^59^ ^]^ Remarkably, the majority of clinical *S. aureus* isolates harbor at least one or up to three prophages.^[^ ^60^ ^]^ Since the transfer of these MGEs commonly occurs at high cell density, we propose that QS‐inhibited CRISPR‐Cas activity could facilitate the transfer of MGEs between cells, and ensure that type III‐A CRISPR‐Cas systems can co‐exist with their targeted MGEs in *S. aureus*, which contributes to staphylococcal pathogenesis and genome evolution. In support of this proposal, we analyzed the antibiotic resistance/virulence genes in the CRISPR‐Cas positive *Staphylococcus* strains using MobileElementFinder. Our analysis revealed that almost all the *S. aureus*, *S. epidermidis*, and *S. pseudintermedius* strains carry the methicillin resistance gene *mecA*, which is typically located on the SCC*mec* element. Furthermore, the prophage‐encoded virulence genes, including staphylokinase (*sak*) and enterotoxins, are frequently detected in *S. aureus*. These findings suggest that horizontal gene transfer from MGEs can still occur even in the presence of a functional CRISPR‐Cas system. This could be attributed to the bacterial inhibition of the CRISPR‐Cas system, such as QS‐mediated inhibition. However, we acknowledge that the number of CRISPR‐Cas positive strains included in our analysis was limited. Deep metagenomic sequences, including CRISPR‐Cas‐negative strains and additional *Staphylococcal* species, could be used in future studies to assess the relationship between CRISPR‐Cas systems, MGEs, and the QS regulatory network. Bioinformatics analysis also revealed that *agr*‐like QS systems are widespread in Gram‐positive bacteria carrying CRISPR‐Cas systems, implying that QS‐mediated repression of CRISPR‐Cas systems may be a common phenomenon in these bacteria.

The repression of CRISPR‐Cas could increase the susceptibility of bacteria to phage attacks. Therefore, other innate immune systems, including surface modifications, restriction‐modifications (RM), and abortive infection systems, are required to interfere with phage infection. In this study, we observed that although the WT strain exhibited reduced phage immunity compared to the QS mutant, it did not completely succumb to phage infection as the ∆CRISPR ∆*cas* mutant did. This suggests that QS‐mediated repression of the type III‐A CRISPR‐Cas system cannot lead to a complete loss of immunity during phage infection. Interestingly, a previous study reported that a type I RM and abortive infection system are located near the type III‐A CRISPR‐Cas system in *S. epidermidis* RP62A strain, indicating potential synergy between these defense systems.^[^ ^61^ ^]^ In support of this, the co‐existence of RM and CRISPR‐Cas system in *S. aureus* and *S. thermophilus* has been shown to work together to enhance immunity.^[^ ^62^, ^63^ ^]^ Consistent with *S. epidermidis* RP62A strain, *S. aureus* TZ0912 strain also contains a type I RM system adjacent to the type III‐A CRISPR‐Cas system.^[^ ^21^ ^]^ It is noteworthy that in *Serratia*, the Rcs membrane stress response pathway represses all three CRISPR‐Cas systems (type I‐E, I‐F, and III‐A) to promote the uptake of resistance genes, while it enhances the surface‐based innate immunity against phage infection.^[^ ^64^ ^]^ In *S. pyogenes* type II‐A CRISPR‐Cas system, a natural single‐guide RNA can direct Cas9 to repress CRISPR‐Cas activation to avoid autoimmunity at the cost of reduced immunity against phages.^[^ ^65^ ^]^ Hence, tightly coordinated and balanced regulation of adaptive and innate immune systems would allow bacteria to optimize their gene acquisition and defense systems according to environmental needs. Since certain proteins encoded by bacteria can inhibit the expression and interference of CRISPR‐Cas systems, plasmids or phages are likely to obtain these protein homologues in response to CRISPR‐Cas immunity. Indeed, it was recently reported that homologues of the CRISPR‐*cas* suppressors AmrZ and RsmA are widely present on plasmids or phages,^[^ ^66^, ^67^ ^]^ implying that these proteins potentially function to overcome CRISPR‐Cas immunity.

We showed that the CRISPR1 array and *cas* genes are transcribed as a single transcript, whereas the *cas* genes downstream of *cas1* can also be transcribed using its own promoter, P*cas*, located within *cas1*. This suggests that the CRISPR1 array and *cas* genes can be separately regulated. Indeed, our results indicated that ArcR functions as a positive regulator for both the *crispr1* array and *cas* genes, whereas SarA has a dual role: repressing *crispr1* expression while activating *cas* genes. This independent regulation may serve to fine‐tune CRISPR‐Cas activity, allowing the cell to maintain a balance between expression of effector proteins and crRNA biogenesis. Similar separate regulation of CRISPR arrays and *cas* genes has also been observed in other bacteria, such as *Sulfolobus islandicus* and the cyanobacterium *Synechocystis*.^[^ ^68^, ^69^ ^]^ The presence of a promoter in the CRISPR leader sequence is in agreement with the type I CRISPR‐Cas systems in *E. coli* and *Pectobacterium atrosepticum* (*P. atrosepticum*).^[^ ^44^, ^70^ ^]^ In contrast to previous studies reporting that *cas1* has no effect on antiplasmid immunity in *S. aureus* and *S. epidermidis* type III‐A CRISPR‐Cas system,^[^ ^22^, ^71^ ^]^ our results showed that mutation of *cas1* (including ∆P*cas*+1+597, ∆P*cas*+298+597, and ∆P*cas*+448+597 mutant strains) led to weaker antiphage immunity than the WT strain, likely because this abolishes expression of the downstream *cas* genes. Additionally, our RNA‐seq results showed that the transcriptional expression level of *cas1* gene was not abundant, which probably explains why spacer adaptation was not observed in the type III‐A CRISPR‐Cas system under the experimental conditions.

Several transcriptional regulators have been shown to control the expression of CRISPR‐*cas* systems.^[^ ^24^ ^]^ For instance, the histone‐like nucleoid‐structuring (H‐NS) DNA‐binding protein was reported to repress the type I‐E CRISPR‐*cas* expression in *E. coli*.^[^ ^44^ ^]^ The extracytoplasmic function (ECF) σ factor DdvS has also been implicated in type III‐B CRISPR‐*cas* activation by binding to the promoter of *cas* genes in *Myxococcus xanthus*.^[^ ^72^ ^]^ Likewise, we find that the regulation of the type III‐A CRISPR‐Cas system in *S. aureus* is controlled by transcriptional regulators AgrA, SarA, and ArcR in this study. In *S. aureus*, SarA is a global transcriptional regulator that binds to an AT‐rich site. It can modulate the expression of multiple target genes involved in virulence.^[^ ^40^ ^]^ Mutation of *sarA* in *S. aureus* resulted in a significant decrease in biofilm formation, indicating that SarA is crucial for biofilm formation.^[^ ^73^ ^]^ Several studies have confirmed that phage therapy is an important method for the inhibition of *S. aureus* biofilm formation.^[^ ^74^, ^75^ ^]^ Here, we show that SarA functions as a positive regulator of the CRISPR‐Cas system, thus protecting bacteria from phage attack. Our study connects the regulation of biofilm formation and the activity of CRISPR‐Cas system to the common regulator SarA, suggesting that SarA‐mediated activation of CRISPR‐Cas system could act as an alternative antiphage defense mechanism when biofilms are destroyed by phages. Additionally, our results indicated that the transcriptional regulator SarR can indirectly repress the expression of *cas* genes. SarR is a negative regulator of *sarA* expression in *S. aureus*.^[^ ^76^ ^]^ Therefore, it is likely that SarR decreases CRISPR‐Cas expression by repressing the direct regulator SarA. With the exception of transcriptional regulation, the CRISPR‐Cas systems can also be controlled through post‐transcriptional regulatory pathways. For example, a recent study on *Serratia* demonstrated that the dimeric RNA‐binding protein RsmA directly affects CRISPR‐Cas adaptive immunity by binding to *cas* transcripts.^[^ ^66^ ^]^

ArcR can control the expression of numerous genes required for anaerobic growth of *S. aureus* by binding to a conserved CRP‐like sequence motif TGTGA‐N_6_‐TCACA.^[^ ^41^ ^]^ In the current study, we determined that ArcR activates the type III‐A CRISPR‐Cas activity by binding to the CRP‐like sequence motif in the P*crispr1* and P*cas* sequences. CRP‐mediated regulation of type I and III CRISPR‐Cas systems has also been reported in many bacteria,^[^ ^77^, ^78^, ^79^, ^80^ ^]^ suggesting that it might be a general phenomenon in diverse bacteria. ArcR utilizes arginine as an energy source for growth under anaerobic conditions, which can be repressed by glucose in *S. aureus*.^[^ ^41^ ^]^ Hence, the ArcR‐activated CRISPR‐Cas system could be suppressed when glucose serves as the main energy source, thereby reducing unnecessary resource consumption and providing more energy for bacterial growth, which is consistent with the findings observed in *P. atrosepticum*.^[^ ^78^ ^]^ Notably, deletion of *arcR* alone resulted in a slightly weaker immunity against plasmid and phage infection. However, it is interesting that deletion of *arcR* in the ∆*sarA* or ∆*agrA* ∆*sarA* mutant completely abolished the immunity of CRISPR‐Cas system, which was similar to ∆*sarA* mutant, indicating that ArcR could provide a strong immunity in the absence of SarA. This could be explained by the fact that SarA was more competitive than ArcR in binding to the P*crispr1* and P*cas* fragments because several SarA‐binding sites were present in the P*crispr1* and P*cas* sequences. Alternatively, since ArcR exerts its regulatory function under anaerobic conditions^[^ ^41^ ^]^ it is possible that ArcR has a much less pronounced effect on immunity under the physiological culture conditions employed in this study.

Since QS activates the CRISPR‐Cas system in *P. aeruginosa* and *Serratia*, QS inhibitors have been proposed to treat bacterial infections in combination with phage therapy.^[^ ^25^, ^26^ ^]^ However, QS also upregulates some phage receptors, and the use of QS inhibitors in connection with such phages results in an overall promotion of the evolution of CRISPR‐Cas immunity, due to higher primary infection rates of QS‐treated *P. aeruginosa*.^[^ ^30^ ^]^ Similarly, a recent study showed that bacteriostatic antibiotics can promote CRISPR‐Cas immunity against phage infection, implying that the combination of bacteriostatic antibiotics and phage therapy may allow pathogens to maintain both virulence and phage resistance.^[^ ^81^ ^]^ In agreement with these results, our finding that AgrA inhibitor 3‐HBA enhanced the resistance of the WT to phage infection suggested that the combination of QS inhibitors and phages could reduce the efficacy of phage therapy against CRISPR‐Cas positive *S. aureus*. Additionally, previous clinical trials have proven that the *Myoviridae* bacteriophage AB‐SA01 is safe for treating patients with severe *S. aureus* infections, and no phage resistance has evolved in vivo.^[^ ^82^ ^]^ However, the increased prevalence of CRISPR‐Cas system in *S. aureus* may pose a serious challenge to phage therapy, and more clinical trials are required to prove the efficacy of phage therapy.

In summary, we found that the QS system AgrA could indirectly repress the expression and interference of the type III‐A CRISPR‐Cas system in *S. aureus*. We identified two transcriptional regulators, SarA and ArcR, that could activate the *cas* genes expression by binding directly to the CRISPR1 and *cas* promoters. Importantly, we further demonstrated that AgrA could inhibit the expression of *sarA* and *arcR*, underpinning the AgrA‐mediated regulation of the CRISPR‐Cas system in *S. aureus*.

## 4 Experimental Section

### Bacterial Strains and Growth Conditions

The bacterial strains, phage, and plasmids used in the present study are listed in Table S1 (Supporting Information). The *S. aureus* RN4220 or TZ0912 and *E. coli* IM08B were cultured in tryptic soy broth (TSB) and LB Broth media, respectively at 37 °C. When required, the medium was supplemented with ampicillin (100 µg mL^−1^) for *E. coli* and chloramphenicol (15 µg mL^−1^) for *S. aureus* to maintain the pRAB11 or pOS1‐*lacZ* plasmid. The pRAB11 plasmid was induced with 100 ng mL^−1^ anhydrotetracycline (ATc). The pIMAY plasmids used to construct mutants were selected using 25 µg mL^−1^ chloramphenicol for *E. coli* and 15 µg mL^−1^ chloramphenicol for *S. aureus*.

### Construction of Mutants and Plasmids

The oligonucleotides used in this study are listed in Table S2 (Supporting Information). The *S. aureus* TZ0912 mutant strains were generated by the allelic replacement method using temperature‐sensitive pIMAY plasmids as described previously.^[^ ^83^ ^]^ Briefly, ≈1000 bp DNA fragments flanking the genes of interest were amplified using the corresponding primer pairs USR_fw/ USR_rv and DSR_fw/ DSR_rv, and subsequently cloned into the pIMAY plasmid using NEBuilder HiFi DNA Assembly Master Mix. The resulting plasmids were transformed into *E. coli* IM08B and then electroporated into *S. aureus* TZ0912 followed by plating on TSA containing 15 µg mL^−1^ chloramphenicol at 28 °C. Allelic exchange was performed as previously described.^[^ ^83^ ^]^ All mutants were verified by PCR amplification and sequencing.

For the construction of *cas1*+1+597 complementation mutant, the *cas1*+1+597 sequence was amplified by PCR from genomic TZ0912 DNA. The resulting DNA fragment was ligated into the pLI50 plasmid digested with EcoR I and Sal I. The recombinant plasmid was then transformed into the ∆P*cas*+1+597 mutant. For the *sarA* and *arcR* complementation, the fragment encompassing the *sarA* and *arcR* ORF, along with their native promoter sequences, were amplified from genomic TZ0912 DNA using the primers listed in Table S2 (Supporting Information). The resulting DNA fragments were ligated into the pLI50 plasmid digested with EcoR I and Sal I. The recombinant plasmid was then transformed into the ∆*sarA* ∆*arcR* or *∆agrA ∆sarA ∆arcR* mutant. As a control, the same plasmid with *sarA* and *arcR* genes driven by the P*gmk* promoter was introduced into the mutants.

To construct the CRISPR‐targeted plasmid, the protospacer sequence was annealed and cloned into the pRMC2 plasmid between the restriction sites Kpn I and Bgl II to obtain pCR1SP1. To construct plasmids containing the transcriptional fusions P*crispr1‐lacZ and* P*cas‐lacZ*, the P*crispr1* and putative P*cas* sequences were amplified by PCR from *S. aureus* TZ0912. The generated PCR products were cloned into the pOS1‐*lacZ* plasmid digested with BamH I and EcoR I. The pET‐AgrA, pET‐SarA, pET‐SarR and pET‐ArcR plasmids used for purification of AgrA‐His_6_, SarA‐His_6_, SarR‐His_6,_ and ArcR‐His_6_ proteins were generated by cloning the coding regions of the corresponding genes into the pET28a plasmid digested with Nde I and Xho I.

### Retention of Plasmid Assay

*S. aureus* strains were transformed with the untargeted plasmid pRMC2 or CRISPR‐targeted plasmid pCR1SP1. Single fresh *S. aureus* colonies of each transformation experiment with the pRMC2 or pCR1SP1 plasmid were picked and resuspended in 50 µL of TSB. Triplicates of 5 µL were inoculated in 5 mL of TSB with 100 ng mL^−1^ ATc and then incubated at 37 °C for 10 h. After incubation, the cultures were serially diluted and plated on TSB agar and TSB agar with 15 µg mL^−1^ chloramphenicol and incubated overnight at 37 °C before enumerating the CFUs. % CFUs containing plasmid was scored as the number of Cm^R^ CFU divided by the total number of CFU.

### Plaque Assay

*S. aureus* overnight cultures (100 µL) were added to soft TSB agar (0.5% agar) and poured onto TSA plates. After solidification of the top agar, 5 µL of serially diluted phages was spotted onto the top agar and incubated overnight at 37 °C. Full‐plate assays were used to count plaques. Briefly, 100 µL of phage dilution was mixed with 100 µL of *S. aureus* cultures grown for 2 or 10 h, and then incubated for 10 min at 37 °C. Soft TSB agar (5 mL) was then added, and the mixture was poured onto TSA agar. Individual plaques were counted and the efficiency of plaquing (EOP) was calculated.

### Adsorption Rate Assay

Overnight cultures of *S. aureus* were diluted to an OD_600_ of 0.05 in TSB and then incubated at 37 °C with shaking. 500 µL of *S. aureus* cultures grown for 2 or 10 h were mixed with phage phiIPLA‐RODI at a multiplicity of infection (MOI) of 0.1, followed by incubation at 37 °C for 10 min. TSB broth mixed with phage without bacteria was used as the control. The mixtures were then centrifuged at 7000 × *g* for 5 min, and the titer of free phage in the supernatant was determined. The adsorbed phage rate was calculated as follows: adsorption rate (%) = [(initial phage titer – phage titer in the supernatant)/(initial phage titer)] × 100.

### Growth Curve

Overnight cultures of *S. aureus* were diluted to an OD_600_ of 0.05 in TSB with 5 mm CaCl_2_ and infected with phage phiIPLA‐RODI at a MOI of 0.01 in biological triplicate in a 96‐well plate. When indicated, 25 µg mL^−1^ 3‐HBA was added into the cultures. The plate was incubated at 37 °C with shaking for 24 h, and the OD_600_ was measured every 30 min using a microplate reader (TECAN, Spark).

### Expression and Purification of Proteins

AgrA‐His_6_, SarA‐His_6_, SarR‐His_6_ and ArcR‐His_6_ were expressed in *E. coli* BL21(DE3) cells. The cells were grown in 1 liter of LB broth at 37 °C with shaking. At an optical density (OD_600_) of 0.4–0.6, 0.5 mm IPTG (isopropyl‐β‐D‐thiogalactopyranoside) was added to induce protein expression. The cultures were incubated for an additional 16 h at 15 °C with shaking, harvested by centrifugation, resuspended in phosphate buffered saline (137 mm NaCl, 2.7 mm KCl, 8.0 mm Na_2_HPO_4_, 1.5 mm KH_2_PO_4_, pH 7.4), and followed by sonication. After centrifugation, the clarified lysates were purified using an Ni^2+^‐NTA‐agarose affinity column (Novagen) according to the manufacturer's protocol.

### Electrophoretic Mobility Shift Assays (EMSAs)

EMSAs were performed as follows. The labeled and unlabeled P*crispr1*, P*cas*, P*sarA*, and P*arcR* sequences were amplified with the primer pairs PE001/PE002, PE003/PE002, PE017/PE018, PE019/PE018, PE020/PE021, PE021/PE020, PE023/PE024 and PE025/PE024, respectively, using *S. aureus* TZ0912 chromosomal DNA as the template. The DNA fragment P2 promoter, which was used as a positive control, was annealed using the primer pair PE005/PE006. As a negative control probe, the house‐keeping gene *gmk* intragenic DNA of *S. aureus* TZ0912 was also amplified with the primer pair PE007/PE008 by PCR. The PCR products were labeled using FAM‐5′‐end‐labeled primers and then gel‐purified using the MiniBEST Agarose Gel DNA Extraction kit (Takara). The binding reaction was carried out by incubating the labeled probe (20 ng) with increasing concentrations of the protein for 30 min at 37 °C in a 20 µL solution containing the binding buffer (750 mm NaCl, 50 mm Tris‐HCl (pH 7.4), 0.5 mm DTT, 0.5 mm EDTA). Protein‐DNA binding reactions were resolved in a 6% non‐denaturing acrylamide gel in 0.5 × TBE buffer, and images were scanned using a Typhoon FLA 9500 (GE Healthcare).

### β‐Galactosidase Assays

The β‐galactosidase activity was determined using o‐nitrophenyl‐β‐D‐galactopyranoside (ONPG) as described previously.^[^ ^84^ ^]^ Briefly, overnight cultures of *S. aureus* strains containing the P*crispr1‐lacZ* or P*cas*‐*lacZ* reporter were diluted to an OD_600_ of 0.05 in 5 mL TSB and then incubated at 37 °C with shaking for 10 h. The OD_600_ of the cultures was measured, and 1 mL of the cultures was harvested by centrifugation. The cells were resuspended in 1 mL of Z buffer (60 mm Na_2_HPO_4_, 40 mm NaH_2_PHO_4_, 10 mm KCl, and 1 mm MgSO_4_, pH 7.0). The cells were incubated for an additional 15 min at 37 °C with lysostaphin (1 mg mL^−1^), after which 0.2 mL of ONPG (4 g mL^−1^) was added and incubated at 37 °C until the solution turned yellow. 0.4 mL of 1 m Na_2_CO_3_ was added to stop the enzymatic reactions, and the OD_420_ was measured. The β‐galactosidase activity was calculated as Miller Units. The assay was repeated at least three times.

### Total RNA Extraction and qRT‐PCR

Overnight cultures of *S. aureus* strains were diluted to an OD_600_ of 0.05 in 30 mL TSB and infected with phage phiIPLA‐RODI at a MOI of 0.01 (if needed). The cultures were then incubated at 37 °C with shaking. Cells were harvested by centrifugation at the indicated time points. The cells were lysed with lysostaphin, and total RNA was extracted using the RNeasy Mini Kit according to the manufacturer's instructions (Qiagen). The removal of genomic DNA (gDNA) and reverse transcription reactions were performed using the PrimeScript RT Regent Kit with gDNA Eraser (Takara). cDNA was quantified by qRT‐PCR using FastStart Universal SYBR Green Master (ROX) (Roche). The *gyrB* gene was used as an endogenous control for normalization. Each qRT‐PCR analysis was performed with at least three biological replicates.

### RNA‐Seq

RNA libraries were prepared with a TruSeq RNA Sample Prep Kit (Illumina) using 3 µg of total RNA extracted from cultures grown for 9 h and then sequenced on an Illumina HiSeq4000 sequencer. RNA‐seq reads were aligned to the reference genome *S. aureus* TZ0912 using Bowtie2.

### Western Blot

Overnight cultures of *S. aureus* strains were diluted to an OD_600_ of 0.05 in 5 mL TSB and infected with phage phiIPLA‐RODI at a MOI of 0.01. The cultures were then incubated at 37 °C with shaking. Cells were harvested by centrifugation at the indicated time points and then resuspended in 200 µL PBS buffer. After treatment with lysostaphin at 37 °C for 20 min, the sample was lysed by sonication. Clarified whole cell lysate was obtained by centrifuging at 10 000×g for 5 min, and then was mixed with 5×loading buffer, boiled at 100 °C for 10 min. Proteins were loaded onto 4%–20% SDS‐PAGE gel and transferred onto PVDF membrane. The membrane was blocked with PBS containing 0.1% Tween‐20 and 5% nonfat dry milk for 2 h at room temperature. Subsequently, the samples were probed overnight at 4 °C with primary antibodies: Csm3 mouse polyclonal antibodies (PcAb) and RpoB mouse PcAb, respectively. Then the membrane was washed three times in PBST and followed by incubation with HRP‐conjugated goat anti‐mouse IgG secondary antibody for 1 h at room temperature. After washing three times, SuperSignal West Pico PLUS chemiluminescence substrates (ThermoFisher Scientific) were used, and bands were visualized using Amersham Imager 600.

### RT‐PCR

A total of 2 µL of the cDNA product was used as a template for subsequent PCR using primer pairs that amplify regions spanning different combinations of genes. As negative and positive controls, the RNA with gDNA removed and gDNA were used as templates in the PCR reaction, respectively.

### DNA Pull‐Down

DNA pull‐down was performed as described previously, with modifications to the protocol.^[^ ^85^ ^]^ In brief, the P*crispr1* and P*cas* sequences were amplified with the 5′‐biotin‐labeled primer pairs PD001/PE002 and PD005/PE018 by PCR using *S. aureus* TZ0912 chromosomal DNA as the template, respectively. As a negative control probe, the house‐keeping gene *gmk* intragenic DNA of *S. aureus* TZ0912 was also amplified with the 5′‐biotin‐labeled primer pair PD003/PE008 by PCR. The resulting PCR products were gel‐purified using the MiniBEST Agarose Gel DNA Extraction kit (Takara). Overnight cultures of *S. aureus* TZ0912 were diluted to an OD_600_ of 0.05 in 1 L TSB and then incubated at 37 °C with shaking for 10 h. The cells were harvested by centrifugation, resuspended in BS/THES buffer (22 mm Tris‐HCl pH = 7.5, 4.4 mm EDTA, 8.9% (w/v) sucrose, 62 mm NaCl, 0.7% Protease Inhibitor Cocktail (v/v), 10 mm HEPES, 5 mm CaCl_2_, 50 mm KCl, 12% glycerol). After treatment with lysostaphin at 37 °C for 1 h, the sample was lysed by sonication. The clarified lysate was obtained by centrifuging twice. The 200 µL Dynabeads M‐280 Streptavidin (Invitrogen) was washed twice with 500 µL 2×B/W buffer (10 mm Tris HCl pH 7.5, 1 mm EDTA, 2 m NaCl) before incubating the beads and 40 µg DNA probe at 25 °C with shaking for 1 h. The bead‐DNA complex was washed twice with 400 µL TE buffer (0.5 m Tris‐HCl, pH 8.0; 1 mM EDTA, pH 8.0) and then twice with 500 µL BS/THES buffer. The bead‐DNA was resuspended in 500 µL of BS/THES buffer supplemented with 10 µg mL^−1^ poly(dI‐dC) (Sigma) and mixed with 700 µL clarified lysate at 25 °C for 30 min. The bead‐DNA was washed five times with BS/THES buffer containing 10 µg mL^−1^ poly(dI‐dC), and then twice with BS/THES. The bound proteins were eluted with increasing NaCl concentrations (0.1, 0.2, 0.3, 0.5, 0.75, 1 m). The eluates were subjected to SDS‐PAGE followed by silver staining and mass spectrometry.

### Phylogenetic Analysis

To identify the P*crispr1* and P*cas* homologs in Firmicutes, the sequences were used as queries against the NCBI nr nucleotide database. Hits with sequence identity >65% and query coverage >35% were retained for further analysis. The representative sequences were aligned using Clustal Omega, and phylogenetic trees were constructed using the neighbor‐joining clustering method. Branch support was computed using 1000 bootstrap replicates. The tree data were visualized and modified using iTOL software.

### Statistical Analysis

GraphPad Prism 8.0 software was used to perform statistical analyses. Error bars and number of replicates for each experiment are defined in the figure legends. Statistical significance between groups was determined by two‐tailed Student's *t*‐test. Statistical significance was considered to be represented by *P* values of <0.05.

## Conflict of Interest

The authors declare no conflict of interest.

## Author Contributions

L.Q.C., J.X.A., H.I., N.M.H.K., and L.Y. conceived and designed the study; L.Y. performed all the experiments with the help of T.Y.Y. and L.X.F.; L.Y. and L.X.F. analyzed RNA‐seq and mass spectrometry proteomics data; L.Q.C. and J.X.A. supervised the project; L.Y. and L.Q.C. wrote the manuscript. All authors contributed to data analysis and writing.

## Supporting information



Supporting Information

Supporting Information

Supporting Information

Supporting Information

## Data Availability

The whole genome sequence of TZ0912 strain has been deposited in the GenBank under accession number CP142839.1. The RNA‐seq data have been deposited in the NCBI Sequence Read Archive under accession number PRJNA836752. The mass spectrometry proteomics data have been deposited in the ProteomeXchange under accession number PXD033796.
