# Supplementary material for: Quorum Sensing Inhibits Type III‐A CRISPR‐Cas System Activity Through Repressing Positive Regulators SarA and ArcR in Staphylococcus Aureus
Source: Adv Sci (Weinh). 2025 Jul 11;12(36):e06049. doi: 10.1002/advs.202506049 (PMC12462963; doi:10.1002/advs.202506049)
Supplement: Supplementary file 1 — Supporting Information [file ADVS-12-e06049-s001.pdf]

ADVANCED SCIENCE
Open Access

# Supporting Information

for *Adv. Sci.*, DOI 10.1002/advs.202506049

Quorum Sensing Inhibits Type III-A CRISPR-Cas System Activity Through Repressing Positive Regulators SarA and ArcR in *Staphylococcus Aureus*

*Yang Li, Yuanyue Tang, Xiaofei Li, Nina Molin Høyland-Kroghsbo, Hanne Ingmer*, Xinan Jiao* and Qiuchun Li**

**SUPPLEMENTARY MATERIAL**

# Quorum sensing inhibits Type III-A CRISPR-Cas system activity through repressing positive regulators SarA and ArcR in *Staphylococcus aureus*

Yang Li[1,2,†], Yuanyue Tang[1,2 ,†], Xiaofei Li[1,2,†], Nina Molin Høyland-Kroghsbo[3], Hanne Ingmer[4,*], Xinan Jiao[1,2,5,*], Qiuchun Li[1,2,5,*]

[1] Jiangsu Key Lab of Zoonosis/Jiangsu Co-Innovation Center for Prevention and Control of Important Animal Infectious Diseases and Zoonoses, Yangzhou University, Jiangsu, China;
[2] Key Laboratory of Prevention and Control of Biological Hazard Factors (Animal Origin) for Agri-food Safety and Quality, Ministry of Agriculture of China, Yangzhou University, Jiangsu, China;
[3] Department of Plant and Environmental Sciences, University of Copenhagen, Copenhagen, Denmark;
[4] Department of Veterinary and Animal Sciences, University of Copenhagen, Copenhagen, Denmark;
[5] Joint International Research Laboratory of Agriculture and Agri-Product Safety, Yangzhou University, Jiangsu, China.
* To whom correspondence should be addressed. Tel: (0086) 514-87971136; Email: qcli@yzu.edu.cn
Correspondence may also be addressed to Xinan Jiao, Email: jiao@yzu.edu.cn and Hanne Ingmer, Email: hi@sund.ku.dk
[†] The authors wish it to be known that, in their opinion, the first three authors should be regarded as joint First Authors.

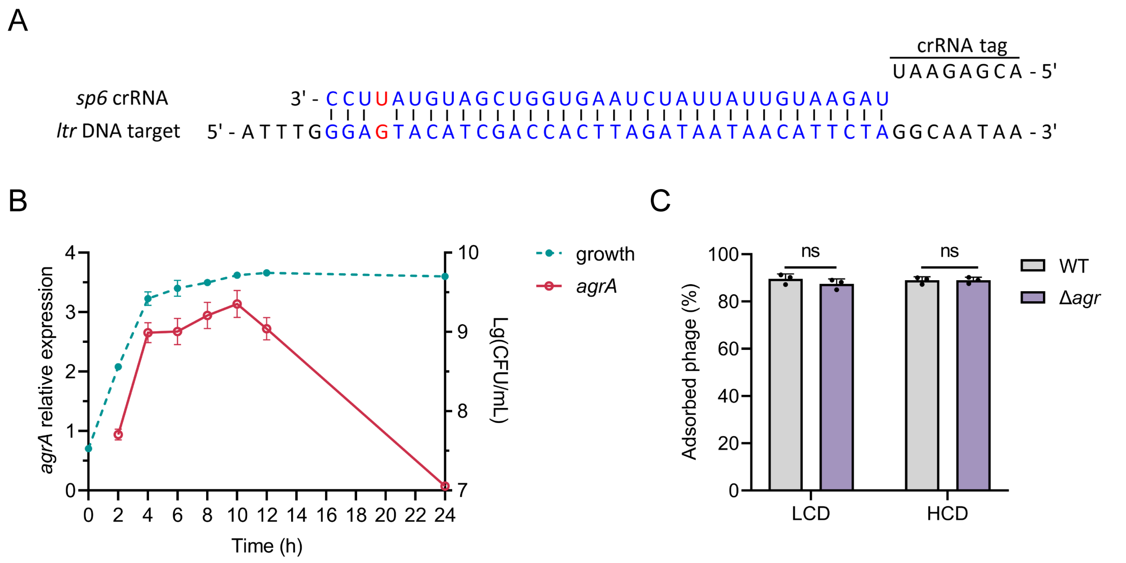


**Figure S1. QS mediates the susceptibility to phage infections in *S. aureus*.** (**A**) Spacer 6 in the CRISPR1 array encodes a crRNA that targets the *ltr* gene of the *Staphylococcus* phage phiIPLA-RODI. There is one base (in red) mismatch between the *sp6* crRNA and the *ltr* protospacer. (**B**) Growth curve of *S. aureus* TZ0912 strain and *agrA* mRNA levels. The transcriptional level of *agrA* was measured by qRT-PCR. The *gyrB* was used as an internal control. (**C**) Adsorption of phage phiIPLA-RODI onto WT and Δ*agrA* mutant cultured for 2 h and 10 h, respectively. All the data shown above (**B** and **C**) are means ± standard deviation of three independent experiments. A two-tailed unpaired Student's *t*-test was used to calculate *P* values; ns, not significant.

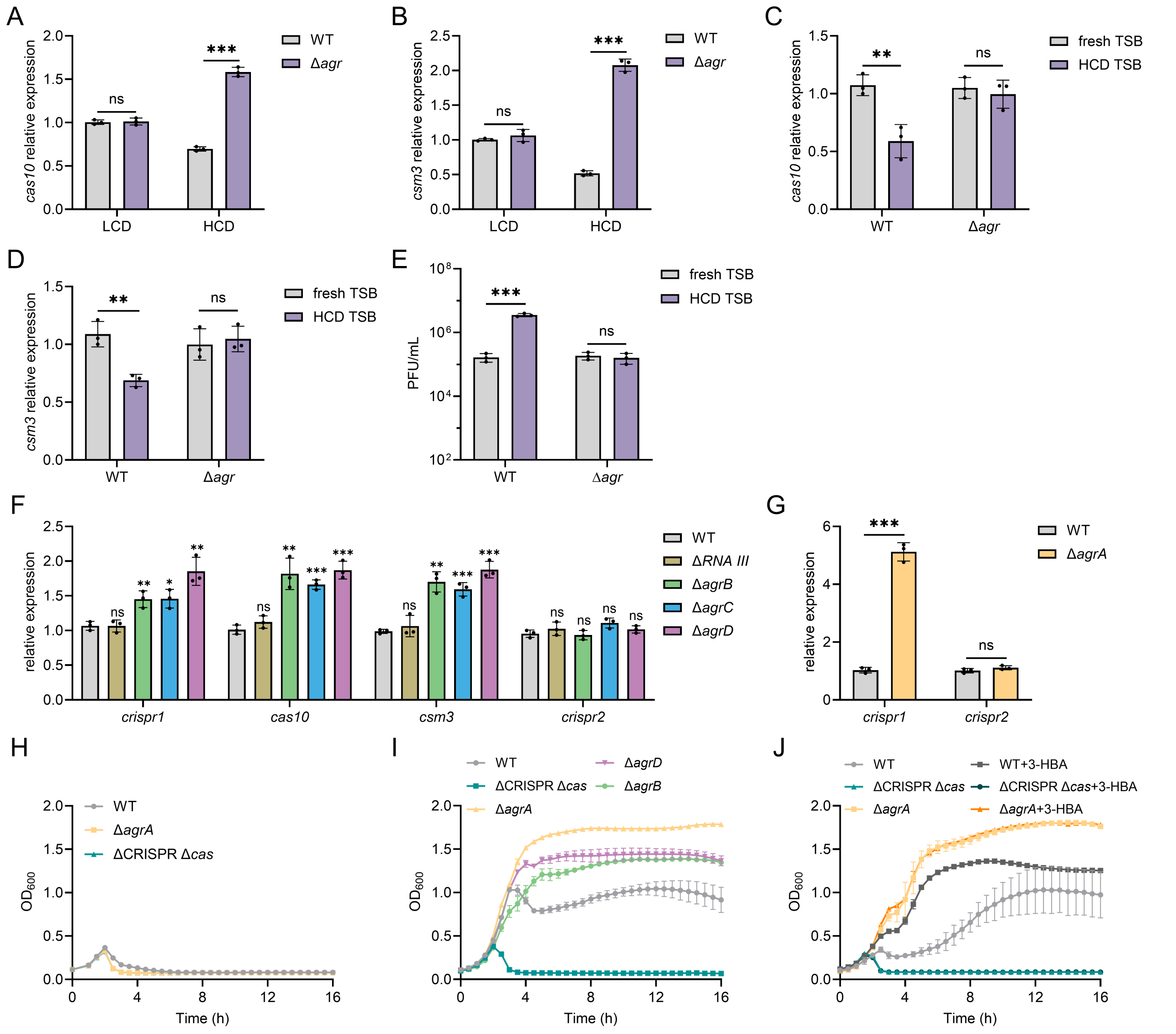


**Figure S2. The immunity against phage infection of QS mutant.** (**A**) The expression of *cas10* was measured by qRT-PCR in the WT and Δ*agr* mutant at both LCD and HCD, respectively. The *gyrB* gene was used as the internal control. (**B**) Same as (A), but the *csm3* expression was assessed. (**C**) The expression of *cas10* was measured by qRT-PCR in the WT and Δ*agr* mutant at LCD. The *gyrB* gene was used as the internal control. Fresh TSB refers to freshly prepared and sterile TSB. HCD TSB refers to a mixture of fresh TSB and HCD culture supernatant at a ratio of 50:1. (**D**) Same as (C), but the *csm3* expression was assessed. (**E**) Phage titers were measured at LCD after infection with phage phiIPLA-RODI at an MOI of 0.01 on the WT and Δ*agr* strains grown in fresh TSB or HCD TSB. (**F**) qRT-PCR analysis of *crispr1*, *crispr2*, *cas10* and *csm3* expression in the WT, Δ*RNA III*, Δ*agrB*, Δ*agrC*, and Δ*agrD* mutant strains at HCD after infection with phage phiIPLA-RODI at an MOI of 0.01. (**G**)

qRT-PCR analysis of *crispr1* and *crispr2* expression in the WT and Δ*agrA* mutant at HCD after infection with phage phiIPLA-RODI at an MOI of 0.01. (**H**) Growth curve of the WT, Δ*agrA*, and ΔCRISPR Δ*cas* strains infected with phage phiSA012 at an MOI of 0.01. (**I**) Growth curve of the WT, ΔCRISPR Δ*cas,* Δ*agrA*, Δ*agrD* and Δ*agrB* strains infected with phage phiIPLA-RODI at an MOI of 0.01. (**J**) Growth curve of the WT, Δ*agrA*, and ΔCRISPR Δ*cas* strains infected with phage phiIPLA-RODI at an MOI of 0.01 in the presence or absence of 3-HBA treatment. All the data shown above (**A**-**J**) shown are means ± standard deviation of three independent experiments. A two-tailed unpaired Student's *t*-test was used to calculate *P* values; ns, not significant, ***$p$<0.001.

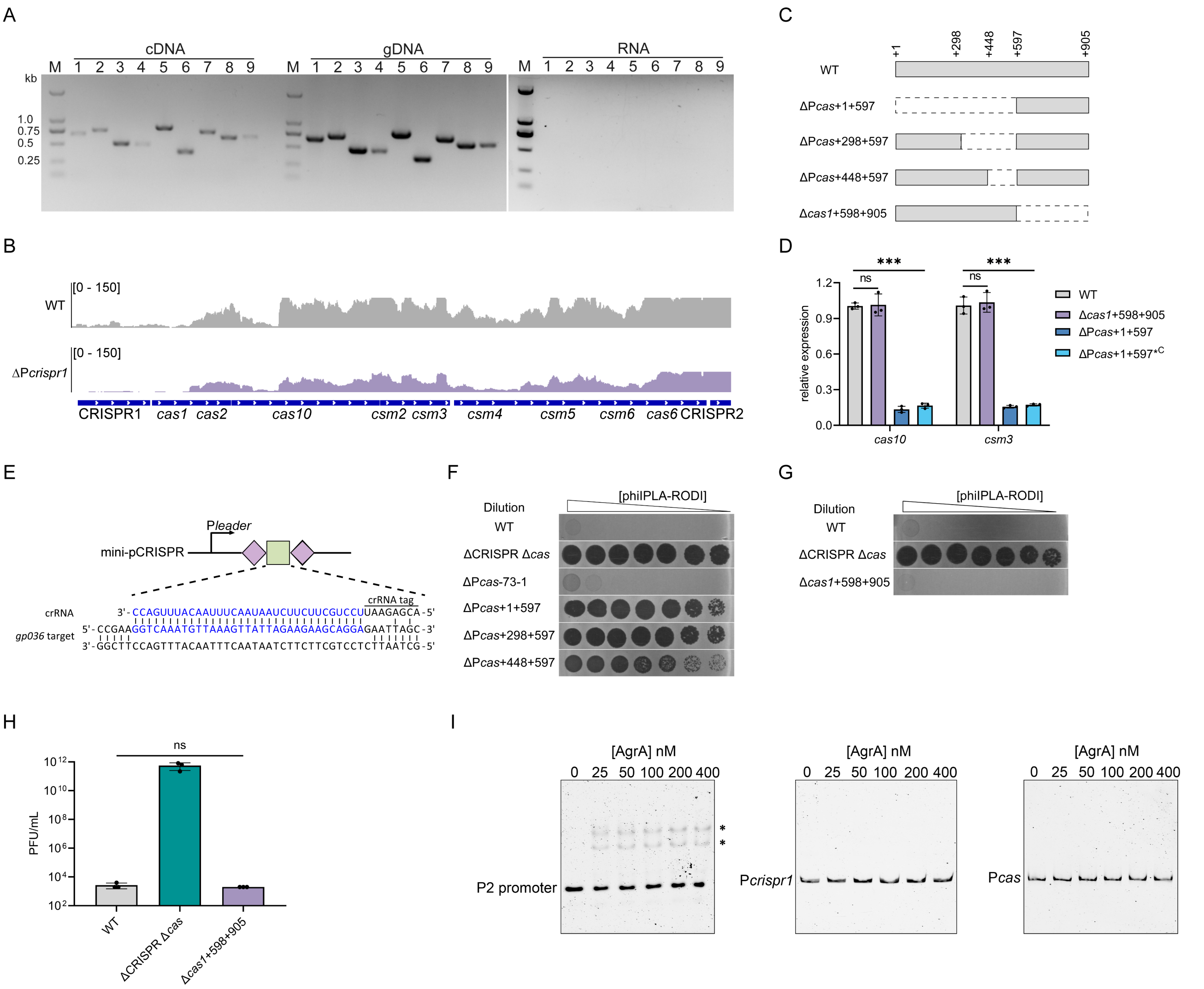


**Figure S3. AgrA cannot bind to the P*crispr1* and P*cas* sequence.** (**A**) RT-PCR amplification of intergenic regions was conducted using cDNA, gDNA and RNA as templates, respectively. The amplified intergenic regions were indicated by numbers that correspond to the as black horizontal lines numbered 1-9 in Fig. 1B. M, 2 kb DNA ladder. (**B**) RNA-seq reads were mapped to the WT and ΔP*crispr1* genome to determine the relative abundance of complete type III-A CRISPR-Cas system expression by IGV software. (**C**) Schematic diagram of the respective P*cas* mutants used in this study. The deleted region was indicated by dashed box. (**D**) qRT-PCR measurement of *cas10* and *csm3* expression in the WT, Δ*cas1*+598+905, ΔP*cas*+1+597 and ΔP*cas*+1+597$^{*C}$ mutants after 10 h of growth in liquid culture, respectively. (**E**) The pCRISPR plasmid encodes a spacer that targets the *gp036* gene of phage phiIPLA-RODI. The crRNA and target sequences are shown in blue. (**F**) Tenfold serial dilution of phage phiIPLA-RODI was spotted on the bacterial lawns of *S. aureus* WT and mutant strains carrying the pCRISPR plasmid that expresses an additional crRNA for enhanced immunity.

Plaquing experiments were replicated three times and consistent results were seen. (**G**) Tenfold serial dilution of phage phiIPLA-RODI was spotted on the bacterial lawns of WT, ΔCRISPR Δ*cas* and Δ*cas1*+598+905 mutant carrying the pCRISPR plasmid. Plaquing experiments were replicated three times and consistent results were seen. (**H**) Phage titers were measured after infection with phage phiIPLA-RODI on the bacterial lawns of *S. aureus* WT, ΔCRISPR Δ*cas*, and Δ*cas1*+598+905 mutant carrying the pCRISPR plasmid. (**I**) FAM-5'-end-labelled P*crispr1* and P*cas* DNA fragments were incubated with increasing concentrations of purified AgrA-$His_6$ protein. Acetyl phosphate (50 mM) was added to all EMSAs. The P2 promoter, a promoter of the *agr* QS system that is regulated directly by AgrA, was used as a positive control. DNA-protein complexes are indicated by an asterisk. All the data shown above (**D** and **H**) shown are means ± standard deviation of three independent experiments. A two-tailed unpaired Student's *t*-test was used to calculate *P* values; ns, not significant, ***$p$<0.001.

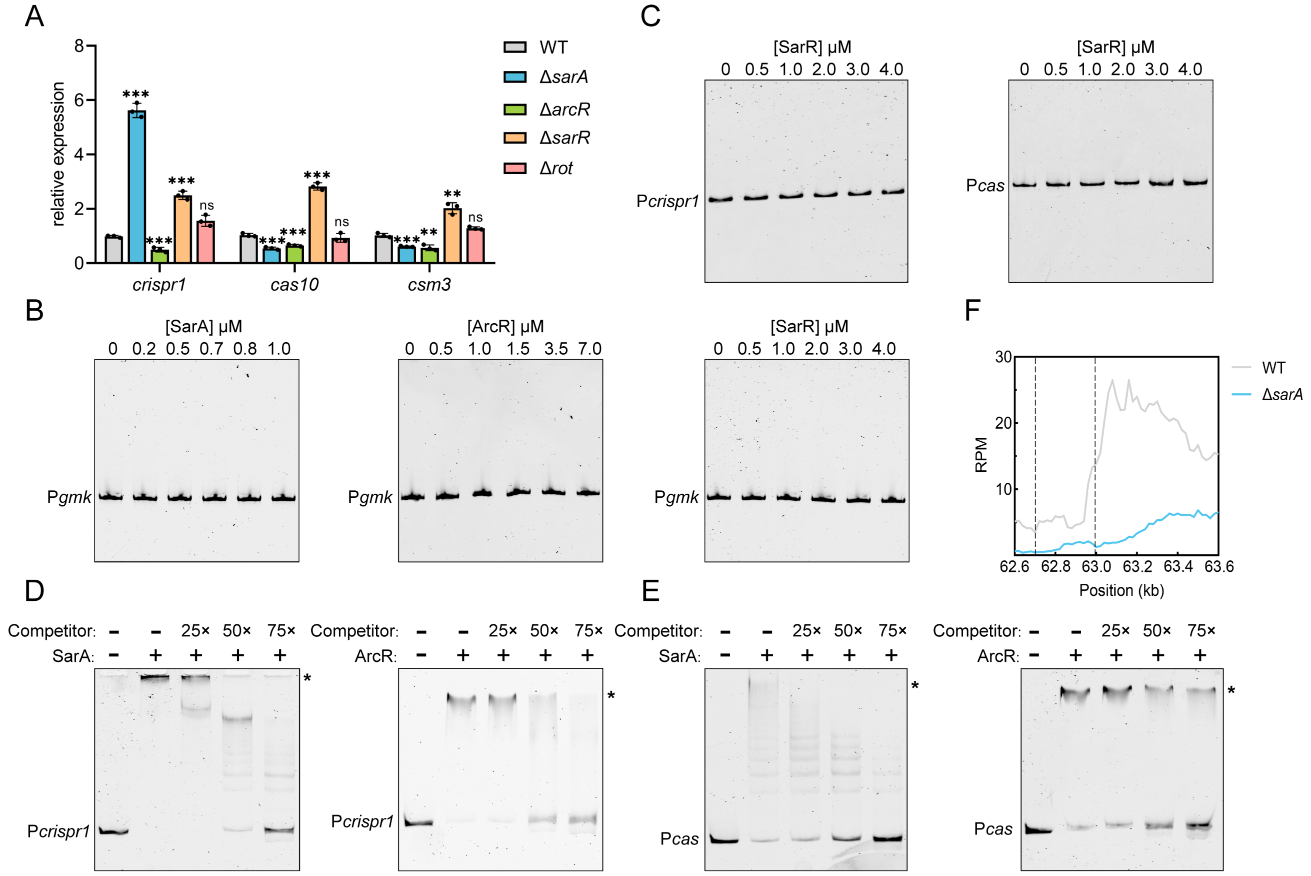


**Figure S4. SarA and ArcR cannot interact with negative probe.** (A) qRT-PCR analysis of *crispr1*, *cas10* and *csm3* expression in the WT and the individual mutant strains at HCD without phage infections, respectively. The data are means ± standard deviation of three independent experiments. A two-tailed unpaired Student's *t*-test was used to calculate *P* values; ns, not significant, $**p<0.01$, $***p<0.001$. (**B**) FAM-5'-end-labelled DNA fragment of P*gmk* was incubated with increasing concentration of purified SarA-His$_6$, ArcR-His$_6$ and SarR-His$_6$ protein. The DNA fragment of *gmk* was used as a negative control. (**C**) FAM-5'-end-labelled P*crispr1* and P*cas* sequence was incubated with increasing concentration of purified SarR-His$_6$ protein, respectively. (**D**) Competitive EMSAs. FAM-5'-end-labelled P*crispr1* sequence was incubated with 1.0 μM purified SarA-His$_6$ and 3.0 μM purified ArcR-His$_6$ in the absence or presence of 25-, 50-, and 75- fold excess of unlabeled P*crispr1* sequence competitors. DNA-protein complexes are indicated by an asterisk. (**E**) Same assay as in (D). FAM-5'-end-labelled P*cas* sequence was incubated with 1.0 μM purified SarA-His$_6$ and 7.0 μM purified ArcR-His$_6$ in the absence or presence of 25-, 50-, and 75- fold excess of unlabeled P*cas* sequence competitors. DNA-protein complexes are indicated by an asterisk. (F) RNA-seq reads were mapped to the WT and Δ*sarA* genome to determine the relative abundance (reads per million mapped reads, RPM) of P*cas* sequence. Position of P*cas* sequence is indicated with vertical dotted lines.

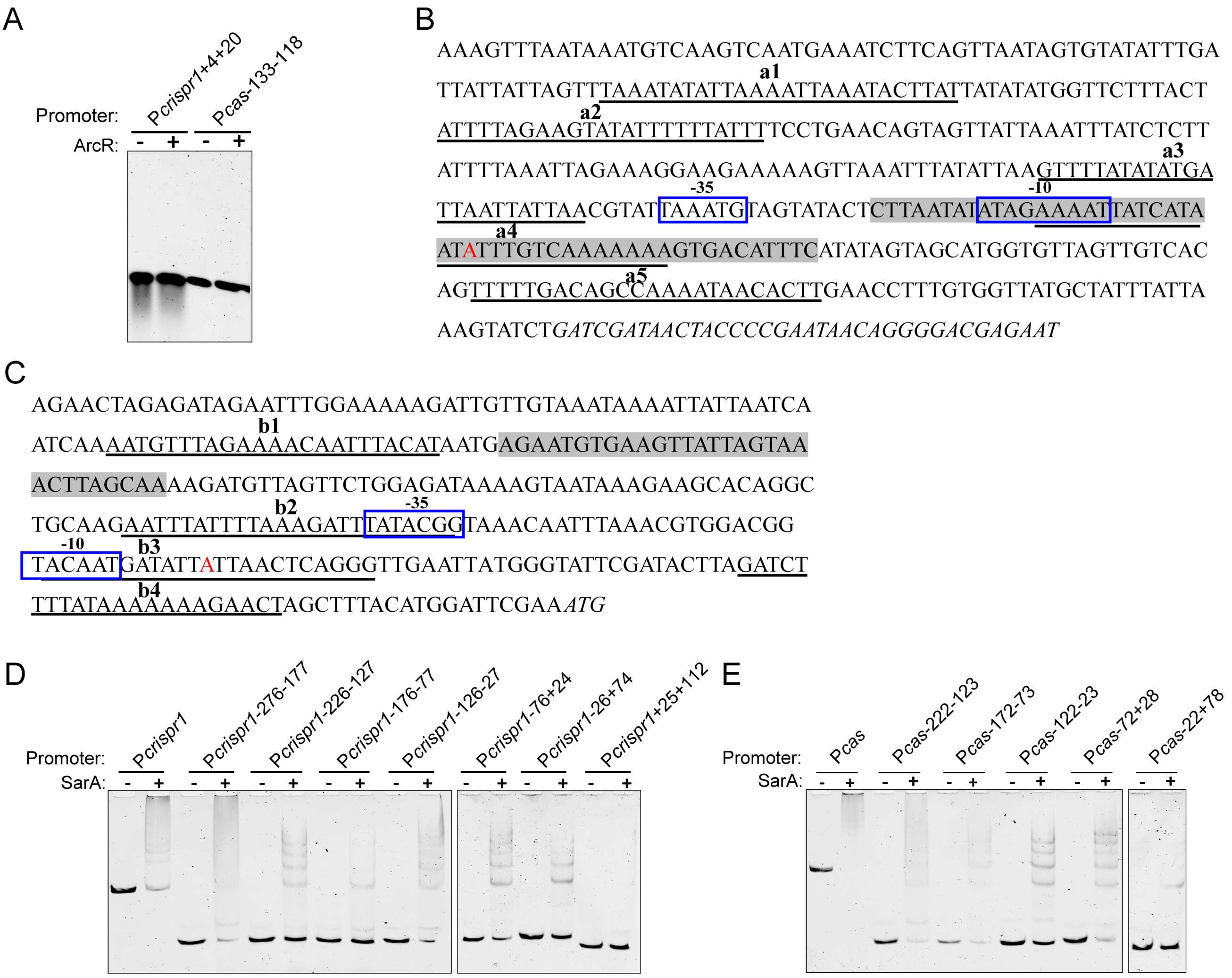


**Figure S5. Determination of ArcR and SarA binding sites in P*crispr1* and P*cas* sequence by EMSA.** (**A**) FAM-5'-end-labelled P*crispr1*+4+20 and P*cas*-133-118 fragment was incubated with ArcR-His$_6$ at the concentration of 7.2 μM, respectively. (**B**) Nucleotide sequence of the P*crispr1* sequence. Transcriptional start site predicted by RNA-seq is indicated in red. -10 and -35 regions, labeled in blue, are also boxed. ArcR binding site is shadowed in gray. SarA binding sites (a1-a5) are underlined. The first repeat of the CRISPR1 array is marked in italic font. (**C**) Nucleotide sequence of the P*cas* sequence. Transcriptional start site predicted by RNA-seq is indicated in red. -10 and -35 regions, labeled in blue, are also boxed. ArcR binding site is shadowed in gray. SarA binding sites (b1-b4) are underlined. The ATG position from +598 to +600 of *cas1* is marked in italic font. (**D**) Seven truncated FAM-5'-end-labelled P*crispr1* fragments were incubated with SarA-His$_6$ at the concentration of 1.0 μM, respectively. (**E**) Five truncated FAM-5'-end-labelled P*cas* fragments were incubated with SarA-His$_6$ at the concentration of 1.0 μM, respectively. DNA-protein complexes are indicated by an asterisk. +, with protein; –, without protein. (**F**) RNA-seq reads were mapped to the

WT and Δ*sarA* genome to determine the relative abundance of P*cas* sequence. Position of P*cas* sequence is indicated with vertical dotted lines.

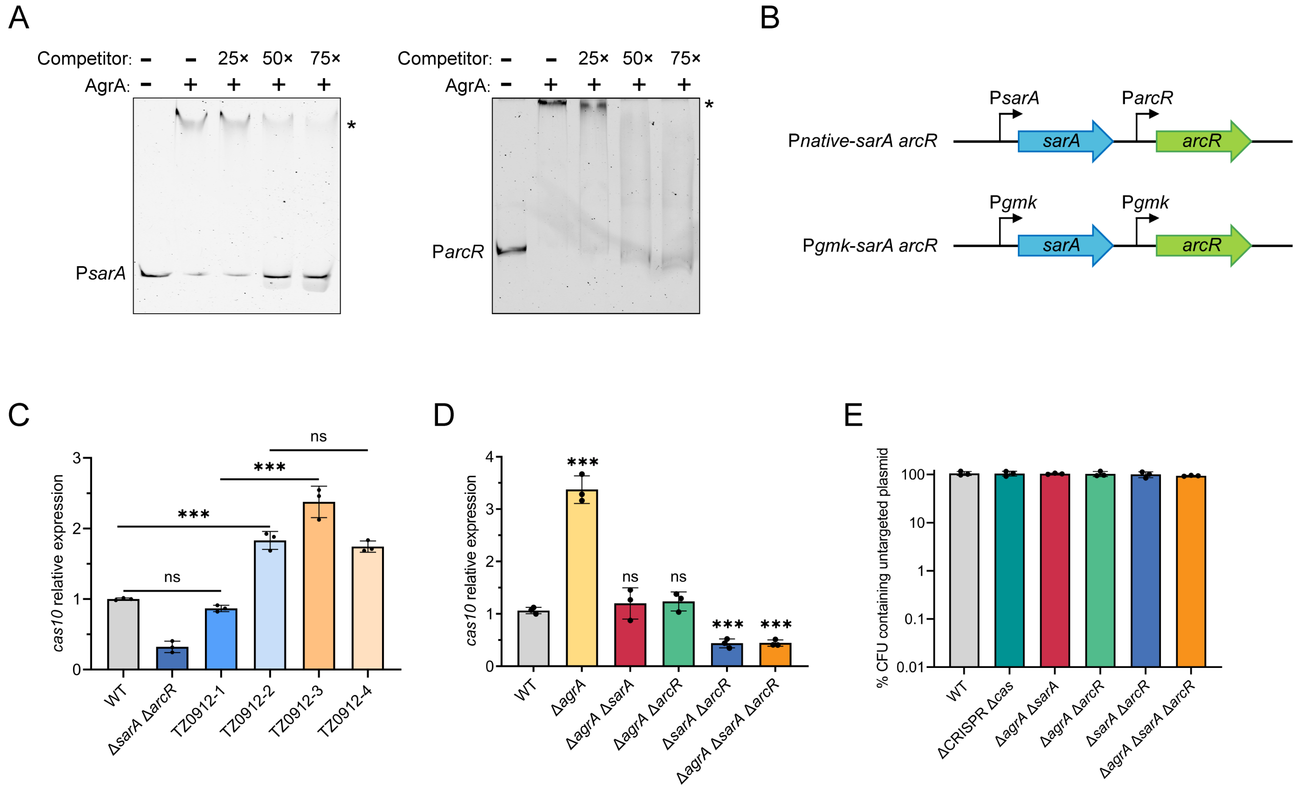


**Figure S6. AgrA specifically binds to the P*sarA* and P*arcR* sequence.** (**A**) Competitive EMSAs. FAM-5'-end-labelled P*sarA* sequence or P*arcR* sequence was incubated with 2 μM purified AgrA-$His_6$ protein in the absence or presence of 25-, 50-, and 75- fold excess of unlabeled P*sarA* sequence or P*arcR* sequence competitors, respectively. DNA-protein complexes are indicated by an asterisk. (**B**) Schematic diagram of the P*native-sarA arcR and* P*gmk-sarA arcR* plasmids used in this study. (**C**) qRT-PCR measurement of *cas10* expression in the WT, Δ*sarA* Δ*arcR*, TZ0912-1 (Δ*sarA* Δ*arcR*::P*native-sarA arcR*), TZ0912-2 (Δ*sarA* Δ*arcR::*P*gmk-sarA arcR*), TZ0912-3 (Δ*agrA* Δ*sarA* Δ*arcR::*P*native-sarA arcR*), and TZ0912-4 (Δ*agrA* Δ*sarA* Δ*arcR::*P*gmk-sarA arcR*) strains after 10 h of growth in liquid culture, respectively. (**D**) qRT-PCR measurement of *cas10* expression in the WT, Δ*agrA*, Δ*sarA*, Δ*arcR*, and double mutants (Δ*agrA* Δ*sarA*, Δ*agrA* Δ*arcR,* Δ*sarA* Δ*arcR*), and triple mutant Δ*agrA* Δ*sarA* Δ*arcR* of the 08BA02176 strain after 10 h of growth in liquid culture, respectively. (**E**) Retention of the untargeted plasmid pRMC2 in the WT, ΔCRISPR Δ*cas*, double mutants (Δ*agrA* Δ*sarA*, Δ*agrA* Δ*arcR,* Δ*sarA* Δ*arcR*), and triple mutant Δ*agrA* Δ*sarA* Δ*arcR*. The strains containing the pRMC2 plasmid were grown in TSB with ATc for 10 h followed by plating on TSA with or without chloramphenicol. % CFUs represents the ratio of bacteria grown on plates containing chloramphenicol to the number of bacteria growing on plates without antibiotics. All the data shown above (**C**-E) are

means ± standard deviation of three independent experiments. A two-tailed unpaired Student's *t*-test was used to calculate *P* values; ns, not significant, ***$p<0.001$.

**Supplementary Table S1. Strains, phages and plasmids used in this study.**

| Bacteria strain, phage or plasmid | Description | Source |
|---|---|---|
| **Strains** | | |
| *E. coli* IM08B | Lab strain used for cloning | (1) |
| RN4220 | NCTC8325-4 derivative, transformable with *E. coli* DNA | Laboratory stock |
| Wild Type | Wild Type *S. aureus* TZ0912, clinical isolate of methicillin-resistant *S. aureus* from human | Laboratory stock |
| ΔCRISPR Δ*cas* | TZ0912 lacking entire CRISPR-*cas* locus | (2) |
| Δ*agr* | TZ0912 lacking entire *agr* locus | This study |
| Δ*agrA* | TZ0912 lacking *agrA* gene | This study |
| Δ*agrB* | TZ0912 lacking *agrB* gene | This study |
| Δ*agrC* | TZ0912 lacking *agrC* gene | This study |
| Δ*agrD* | TZ0912 lacking *agrD* gene | This study |
| Δ*RNA III* | TZ0912 lacking *RNA III* gene | This study |
| ΔCRISPR Δ*cas* Δ*agr* | TZ0912 lacking entire CRISPR-*cas* locus and *agr* gene | This study |
| Δ*sarR* | TZ0912 lacking *sarR* gene | This study |
| Δ*rot* | TZ0912 lacking *rot* gene | This study |
| Δ*sarA* | TZ0912 lacking *sarA* gene | This study |
| Δ*arcR* | TZ0912 lacking *arcR* gene | This study |
| Δ*agrA* Δ*sarA* | TZ0912 lacking *agrA* and *sarA* gene | This study |
| Δ*agrA* Δ*arcR* | TZ0912 lacking *agrA* and *arcR* gene | This study |
| Δ*sarA* Δ*arcR* | TZ0912 lacking *sarA* and *arcR* gene | This study |
| Δ*agrA* Δ*sarA* Δ*arcR* | TZ0912 lacking *agrA*, *sarA* and *arcR* gene | This study |
| TZ0912-1 | Δ*sarA* Δ*arcR* mutant containing P*native-sarA arcR* plasmid | This study |
| TZ0912-2 | Δ*sarA* Δ*arcR* mutant containing P*gmk-sarA arcR* plasmid | This study |

| | | |
|---|---|---|
| TZ0912-3 | Δ*agrA* Δ*sarA* Δ*arcR* mutant containing P*native-sarA arcR* plasmid | This study |
| TZ0912-4 | Δ*agrA* Δ*sarA* Δ*arcR* mutant containing P*gmk-sarA arcR* plasmid | This study |
| 08BA02176 | A strain carries type III-A CRISPR-Cas system and was isolated from pig farmers in Canada | (3) |
| Δ*agrA* | 08BA02176 lacking *agrA* gene | This study |
| Δ*sarA* | 08BA02176 lacking *sarA* gene | This study |
| Δ*arcR* | 08BA02176 lacking *arcR* gene | This study |
| Δ*agrA* Δ*sarA* | 08BA02176 lacking *agrA* and *sarA* gene | This study |
| Δ*agrA* Δ*arcR* | 08BA02176 lacking *agrA* and *arcR* gene | This study |
| Δ*sarA* Δ*arcR* | 08BA02176 lacking *sarA* and *arcR* gene | This study |
| Δ*agrA* Δ*sarA* Δ*arcR* | 08BA02176 lacking *agrA*, *sarA* and *arcR* gene | This study |
| ΔP*crispr1* | TZ0912 lacking leader sequence of CRISPR 1 array | This study |
| ΔP*cas*-73-1 | TZ0912 lacking 73 bp intergenic region between *CRISPR 1* and *cas1* | This study |
| ΔP*cas*+1+597 | TZ0912 lacking +1 to +597 nucleotides of *cas1* gene | This study |
| ΔP*cas*+298+597 | TZ0912 lacking +298 to +597 nucleotides of *cas1* gene | This study |
| ΔP*cas*+448+597 | TZ0912 lacking +448 to +597 nucleotides of *cas1* gene | This study |
| Δ*cas1*+598+905 | TZ0912 lacking +598 to +905 nucleotides of *cas1* gene | This study |
| ΔP*cas*+1+597*$^{C}$ | ΔP*cas*+1+597 mutant containing p*cas1*+1+597 plasmid | This study |
| **Phages** | | |
| *Staphylococcus* phage phiIPLA-RODI | Phage targeted by CRISPR1 spacer 6; Generous gift from Pilar García, Instituto de Productos Lácteos de Asturias, Spain | (4) |
| *Staphylococcus* phage phiSA012 | Phage untargeted by TZ0912 | (5) |
| **Plasmids** | | |
| pIMAY | Temperature-sensitive plasmid for allelic exchange in *S. aureus*; Cm$^{R}$ in *E. coli* and *S. aureus* | (6) |
| pRMC2 | Empty plasmid; *cat bla tetR*, Pxyl/tet (1×*tetO*), Amp$^{R}$ in *E. coli* and Cm$^{R}$ in *S. aureus* | (7) |

| | | |
|---|---|---|
| pRAB11 | Empty plasmid; pRMC2 with Pxyl/tet (2×*tetO*); $Amp^R$ in *E. coli* and $Cm^R$ in *S. aureus* | (8) |
| pLI50 | Empty plasmid; low-copy-number plasmid, $Amp^R$ in *E. coli* and $Cm^R$ in *S. aureus* | (9) |
| pET28a | T7 expression vector; $Km^R$ | Laboratory stock |
| pCR1SP1 | pRMC2 containing the protospacer to CRISPR1 spacer 1; $Amp^R$ in *E. coli* and $Cm^R$ in *S. aureus* | This study |
| pCRISPR | pRAB11 containing two repeats and a spacer that targets the *gp036* gene of phage phiIPLA-RODI; $Amp^R$ in *E. coli* and $Cm^R$ in *S. aureus* | This study |
| Promoterless-*lacZ* | pOS1-*lacZ* containing a Promoterless *lacZ* reporter; $Amp^R$ in *E. coli* and $Cm^R$ in *S. aureus* | (10) |
| P*crispr1*-*lacZ* | pOS1-*lacZ* containing a P*crispr1*-*lacZ* reporter; $Amp^R$ in *E. coli* and $Cm^R$ in *S. aureus* | This study |
| P*cas*-*lacZ*-73-1 | pOS1-*lacZ* containing a P*cas* (73 bp)-*lacZ* reporter; $Amp^R$ in *E. coli* and $Cm^R$ in *S. aureus* | This study |
| P*cas*-*lacZ*+1+597 | pOS1-*lacZ* containing a P*cas* (597 bp)-*lacZ* reporter; $Amp^R$ in *E. coli* and $Cm^R$ in *S. aureus* | This study |
| P*cas*-*lacZ*+298+597 | pOS1-*lacZ* containing a P*cas* (300 bp)-*lacZ* reporter; $Amp^R$ in *E. coli* and $Cm^R$ in *S. aureus* | This study |
| P*cas*-*lacZ*+448+597 | pOS1-*lacZ* containing a P*cas* (150 bp)-*lacZ* reporter; $Amp^R$ in *E. coli* and $Cm^R$ in *S. aureus* | This study |
| p*cas1*+1+597 | pLI50 containing +1 to +597 nucleotides of *cas1* gene; $Amp^R$ in *E. coli* and $Cm^R$ in *S. aureus* | This study |
| P*native*-*sarA arcR* | pLI50 containing *sarA* and *arcR* gene driven by their native promoter; $Amp^R$ in *E. coli* and $Cm^R$ in *S. aureus* | This study |
| P*gmk*-*sarA arcR* | pLI50 containing *sarA* and *arcR* gene driven by a constitutive promoter P*gmk*; $Amp^R$ in *E. coli* and $Cm^R$ in *S. aureus* | This study |
| pET-AgrA | pET28a containing *agrA* gene; $Km^R$ | This study |
| pET-SarA | pET28a containing *sarA* gene; $Km^R$ | This study |
| pET-ArcR | pET28a containing *arcR* gene; $Km^R$ | This study |
| pET-SarR | pET28a containing *sarR* gene; $Km^R$ | This study |

**Supplementary Table S2. Primers used in this study.**

| Name | Sequence (5'-3') | Description | Restriction sites |
|---|---|---|---|
| **Mutant construction** | | | |
| PM001 | ACGCGGTACCTCACTTTGCGGTGAGAGATG | CRISPR-*cas* deletion; USR*; fw | Kpn I |
| PM002 | CAAGATGAAGATACTTTAATAAATAGCATAACCACAAAGGTTCAAG | CRISPR-*cas* deletion; USR; rv | |
| PM003 | AGTATCTTCATCTTGATGATCCCAATTGGCTTGG | CRISPR-*cas* deletion; DSR*; fw | |
| PM004 | ACGAGCTCTGATTTTGCTAAAAACCTTCAAA | CRISPR-*cas* deletion; DSR; rv | Sac I |
| PM005 | AAATCGGGTTGTTCAAGACC | Check for deletion of CRISPR-*cas*; fw | |
| PM006 | TGTTTCTGTTTATTTAGAAATTGAATG | Check for deletion of CRISPR-*cas*; rv | |
| PM011 | AGGGAACAAAAGCTGGGTACCACGTAGCTGAACCCTTTCAATTG | *agr* deletion; USR; fw | Kpn I |
| PM012 | TAACTGACTTTATTATCTTAGATGAATAATTAATTACTTTCATTGTAAATTTGT | *agr* deletion; USR; rv | |
| PM013 | AAAGTAATTAATTATTCATCTAAGATAATAAAGTCAGTTAACGGCG | *agr* deletion; DSR; fw | |
| PM014 | CTATAGGGCGAATTGGAGCTCCTCTGCTGATATGTTATTTGAACCAA | *agr* deletion; DSR; rv | Sac I |
| PM015 | ATCCCTTCTTCATTACAAA | Check for deletion of *agr*; fw | |
| PM016 | AAAATAAGATTCACGGAGT | Check for deletion of *agr*; rv | |
| PM021 | CTATAGGGCGAATTGGAGCTCATACTGTGCTAACTACATGTATATAGTTATATATG C | *agrA* deletion; USR; fw | Sac I |

| | | | |
|---|---|---|---|
| PM022 | TAACTGACTTTATTATCTTAACATTCACATCCTTATGGCTAGTTGTTAATAATTT | *agrA* deletion; USR; rv | |
| PM023 | AGCCATAAGGATGTGAATGTTAAGATAATAAAGTCAGTTAACGGCGTATTCAATTGTAAATCT | *agrA* deletion; DSR; fw | |
| PM024 | GGGCCCCCCCTCGAGGTCGACATCGTAAGCCCTCTGCTGATATG | *agrA* deletion; DSR; rv | Sal I |
| PM031 | AATACCCGATGAAGTAAGTAGC | Check for deletion of *agAr*; fw | |
| PM032 | TTGCCAACATTACAAGAGGT | Check for deletion of *agrA*; rv | |
| PM053 | AGGGAACAAAAGCTGGGTACCACAGAAGCAACAGCTGAAGCACA | *arcR* deletion; USR; fw | Kpn I |
| PM054 | AAGTTAAACTCCTTCAAACCTTGCA | *arcR* deletion; USR; rv | |
| PM055 | GGTTTGAAGGAGTTTAACTTTATACAATGTAAAATGAATAAGTTGAACATG | *arcR* deletion; DSR; fw | |
| PM056 | CTATAGGGCGAATTGGAGCTCACATTTGTACCTTTAGCATCAGCAG | *arcR* deletion; DSR; rv | Sac I |
| PM059 | GTAAGTTTGCGGAAGGATCG | Check for deletion of *arcR*; fw | |
| PM060 | TCGTTCGATTGTTCTGAAGC | Check for deletion of *arcR*; rv | |
| PM063 | GGGCCCCCCCTCGAGGTCGACCTTATCATTTTAACTTGTAATTATTTTTAAAAGTG | *sarA* deletion; USR; fw | Sal I |
| PM064 | ACAAAAGTTTAAAACCTCCCTATTTGATGCA | *sarA* deletion; USR; rv | |
| PM065 | GGGAGGTTTTAAACTTTTGTTTAGCGCAATTTGGTG | *sarA* deletion; DSR; fw | |
| PM066 | TCCCCCGGGCTGCAGGAATTCGCAACATCAACTAGCATCATCATATAA | *sarA* deletion; DSR; rv | EcoR I |
| PM071 | CCCAGAAATACAATCACTGTGTC | Check for deletion of *sarA*; fw | |
| PM072 | ACCGCATATTGAAGAACCAGA | Check for deletion of *sarA*; rv | |
| PM083 | GGGCCCCCCCTCGAGGTCGACGCGGTTAGCCCGATTTTAAA | *sarR* deletion; USR; fw | Sal I |

| | | | |
|---|---|---|---|
| PM084 | CCTTGATTATTAAACCACTCCTCTGATGCACA | *sarR* deletion; USR; rv | |
| PM085 | GAGTGGTTTAATAATCAAGGTTAATTGCGTTTAATAACAT | *sarR* deletion; DSR; fw | |
| PM086 | TCCCCCGGGCTGCAGGAATTCCAATGGACTGGGCAACTTGATT | *sarR* deletion; DSR; rv | EcoR I |
| PM091 | TTGTGAGCAAGCCATCCAAT | Check for deletion of *sarR*; fw | |
| PM092 | GACTAGTGTACCTTGTTTCAAGC | Check for deletion of *sarR*; rv | |
| PM093 | GGGCCCCCCCTCGAGGTCGACCATCGTGGTGCAACATCCTATC | *rot* deletion; USR; fw | Sal I |
| PM094 | GCTATTAAACAAAACTACAAGTGTAAATAAACTTGCTTTC | *rot* deletion; USR; rv | |
| PM095 | TTGTAGTTTTGTTTAATAGCATAAAAAGAGGTTTTCATT | *rot* deletion; DSR; fw | |
| PM096 | TCCCCCGGGCTGCAGGAATTCGATGGCTACTGCCAAAGAAGTTG | *rot* deletion; DSR; rv | EcoR I |
| PM101 | ACCAATTTAGCCTCATTCGGTT | Check for deletion of *rot*; fw | |
| PM102 | ATGCTCCATTCATTTGTGCCA | Check for deletion of *rot*; rv | |
| PM113 | GGGCCCCCCCTCGAGGTCGACAGCAAGTGATGGAAATGTTAAAACC | P*crispr1* deletion; USR; fw | Sal I |
| PM114 | CGGGGTAGTTATCGATCCTTTAGGTCTTGAACAACCCGATT | P*crispr1*deletion; USR; rv | |
| PM115 | AAGGATCGATAACTACCCCGAATAACAGGGGACGAGAATTCTATAAGTTCATTA ATTCCGATACCTAG | P*crispr1* deletion; DSR; fw | |
| PM116 | TCCCCCGGGCTGCAGGAATTCTACAAATGAATTAATCAGTGACATGAAAG | P*crispr1* deletion; DSR; rv | EcoR I |
| PM121 | TCTTTGGCGAGTTGAATGAG | Check for deletion of P*crispr1*; fw | |
| PM122 | TGATCTGAAAGGGTGGAAAA | Check for deletion of | |

| | | | |
|---|---|---|---|
| | | P*crispr1*; rv | |
| PM123 | GGGCCCCCCCTCGAGGTCGACTTTTTTCCACCCTTTCAGATCATC | P*cas*-73-1 deletion; USR; fw | Sal I |
| PM124 | TCTTTCATCACTCTGTCCCCTGTTATTCGGGATAGTTATCGATCACCGACGTTTTATTACC | P*cas*-73-1 deletion; USR; rv | |
| PM125 | GGGGACAGAGTGATGAAAGATGTTATTTATGTAGAAAATCATTA | P*cas*-73-1 deletion; DSR; fw | |
| PM126 | TCCCCCGGGCTGCAGGAATTCACATCGCTGACATACCTCTCGTATA | P*cas*-73-1 deletion; DSR; rv | EcoR I |
| PM127 | GGTAATAGTTGCTCAATAGG | Check for deletion of P*cas*-73-1; fw | |
| PM128 | TTGCGTTAATGGAGAGTGCTT | Check for deletion of P*cas*-73-1; rv | |
| PM129 | GGGCCCCCCCTCGAGGTCGACTCCACCCTTTCAGATCATCTATGA | P*cas*+1+597 deletion; USR; fw | Sal I |
| PM130 | CTAAACTCATTTTTCATCCCCTAAAAATTAATCAATG | P*cas*+1+597 deletion; USR; rv | |
| PM131 | GGGATGAAAAATGAGTTTAGGCATTAATCATCGTTC | P*cas*+1+597 deletion; DSR; fw | |
| PM132 | TCCCCCGGGCTGCAGGAATTCATTCTTCGTCGCCCTCTTCTATAA | P*cas*+1+597 deletion; DSR; rv | EcoR I |
| PM135 | GCTCAATAGGTAATAAAACGTCGG | Check for deletion of P*cas*+1+597; fw | |
| PM136 | AGGGGTGTTTCTTCATAGCA | Check for deletion of P*cas*+1+597; rv | |
| PM137 | GGGCCCCCCCTCGAGGTCGACTAGAATGTTATTATCTAAGTGGTCGATGTAT | P*cas*+298+597 | Sal I |

| | | | |
|---|---|---|---|
| | | deletion; USR; fw | |
| PM138 | GCCTAAACTCATCCCAGATAATTGAAACTGACTTTGAA | P*cas*+298+597 deletion; USR; rv | |
| PM139 | TATCTGGGATGAGTTTAGGCATTAATCATCGTTC | P*cas*+298+597 deletion; DSR; fw | |
| PM140 | TCCCCCGGGCTGCAGGAATTCATTCTTCGTCGCCCTCTTCTATAA | P*cas*+298+597 deletion; DSR; rv | EcoR I |
| PM143 | GCTCAATAGGTAATAAAACGTCGGT | Check for deletion of P*cas*+298+597; fw | |
| PM144 | ACATCGCTGACATACCTCTCG | Check for deletion of P*cas*+298+597; rv | |
| PM145 | GGGCCCCCCCTCGAGGTCGACCCATGAATAACACCCTCCTTTTTT | P*cas*+448+597 deletion; USR; fw | Sal I |
| PM146 | GCCTAAACTCATTTCTTTATTACTTTTATCTCCAGAACTAACA | P*cas*+448+597 deletion; USR; rv | |
| PM147 | ATAAAGAAATGAGTTTAGGCATTAATCATCGTTC | P*cas*+448+597 deletion; DSR; fw | |
| PM148 | TCCCCCGGGCTGCAGGAATTCCGTATTCTTCGTCGCCCTCTT | P*cas*+448+597 deletion; DSR; rv | EcoR I |
| PM151 | GCACTCTCCATTAACGCAACT | Check for deletion of P*cas*+448+597; fw | |
| PM152 | AGGGGTGTTTCTTCATAGCA | Check for deletion of P*cas*+448+597; rv | |
| PM187 | GGGCCCCCCCTCGAGGTCGACATATTTCTTCCATGAATAACACCCTCC | *cas1*+598+905 deletion; USR; fw | Sal I |
| PM188 | CATTTCGAATCCATGTAAAGCTAGTTCTT | *cas1*+598+905 deletion; | |

| | | | |
|---|---|---|---|
| | | USR; rv | |
| PM189 | CTTTACATGGATTCGAAATGTATTTATTAGTTAGTTTTGACTTACCTAGAGA | *cas1*+598+905 deletion; DSR; fw | |
| PM190 | TCCCCCGGGCTGCAGGAATTCACAACTAGCAATAGCACATGTAATACGA | *cas1*+598+905 deletion; DSR; rv | EcoR I |
| PM193 | AAAGAAGCACAGGCTGCAAG | Check for deletion of *cas1*+598+905; fw | |
| PM194 | ACATCGCTGACATACCTCTCG | Check for deletion of *cas1*+598+905; rv | |
| PM195 | GGGCCCCCCCTCGAGGTCGACGTTGCTGCAGGAGCATATACTGAG | *RNA III* deletion; USR; fw | Sal I |
| PM196 | TCATCCAACTATTTTCCATCACATCTCTGTG | *RNA III* deletion; USR; rv | |
| PM197 | GATGGAAAATAGTTGGATGAATAATTAATTACTTTCATTGTAAATTTG | *RNA III* deletion; DSR; fw | |
| PM198 | TCCCCCGGGCTGCAGGAATTCAGCGCTATCAGAGATCTCGGAA | *RNA III* deletion; DSR; rv | EcoR I |
| PM201 | CACTCTCCTCACTGTCATTATACGA | Check for deletion of *RNA III*; fw | |
| PM202 | CATAGCACTGAGTCCAAGGAAA | Check for deletion of *RNA III*; rv | |
| PM445 | GGGCCCCCCCTCGAGGTCGACCTTAAATGAAGTAGAACAGCAACGC | *agrB* deletion; USR; fw | Sal I |
| PM446 | TTTTACACCACTCTCCTCACTGTCA | *agrB* deletion; USR; rv | |
| PM447 | GTGAGGAGAGTGGTGTAAAAATACATTATTTAACTTATTTTTGATTTTATTACTG | *agrB* deletion; DSR; fw | |
| PM448 | TCCCCCGGGCTGCAGGAATTCTGCTACTTACTTCATCGGGTATTTCG | *agrB* deletion; DSR; rv | EcoR I |
| PM451 | AGCACTGAGTCCAAGGAAAC | Check for deletion of | |

| | | | |
|---|---|---|---|
| | | *agrB*; fw | |
| PM452 | AAGGACGCGCTATCAAACAT | Check for deletion of *agrB*; rv | |
| PM453 | GGGCCCCCCCTCGAGGTCGACTGTTCACTGTGTCGATAATCCATTT | *agrC* deletion; USR; fw | Sal I |
| PM454 | CACATCCTTATGGTTGAGTTAATACGAATAAAACAAAATTATAAC | *agrC* deletion; USR; rv | |
| PM455 | AACTCAACCATAAGGATGTGAATGTATGAAAATT | *agrC* deletion; DSR; fw | |
| PM456 | TCCCCCGGGCTGCAGGAATTCGCCAGCTATACAGTGCATTTGC | *agrC* deletion; DSR; rv | EcoR I |
| PM459 | TCGCAGCTTATAGTACTTGTGAC | Check for deletion of *agrC*; fw | |
| PM460 | CGAGGGCAATTTCCATAGGC | Check for deletion of *agrC*; rv | |
| PM461 | GGGCCCCCCCTCGAGGTCGACTACATAGCACTGAGTCCAAGGAAACT | *agrD* deletion; USR; fw | Sal I |
| PM462 | CTATTTAAATTTAAGTCCTCCTTAATAAAGAAAATAGGT | *agrD* deletion; USR; rv | |
| PM463 | GAGGACTTAAATTTAAATAGAGAGTGTGATAGTAGGTGGAA | *agrD* deletion; DSR; fw | |
| PM464 | TCCCCCGGGCTGCAGGAATTCCATGAATGCGTGGTATATCATCAG | *agrD* deletion; DSR; rv | EcoR I |
| PM465 | CCCATTCCTGTGCGACTTAT | Check for deletion of *agrD*; fw | |
| PM466 | AAGGACGCGCTATCAAACAT | Check for deletion of *agrD*; rv | |
| **Recombinant plasmids construction** | | | |
| PR013 | GTGCCGCGCGGCAGCCATATGATGAAAATTTTCATTTGCGAAGAC | pET-AgrA; fw | Nde I |
| PR014 | GTGGTGGTGGTGGTGCTCGAGTTATATTTTTTTAACGTTTCTCACCGA | pET-AgrA; rv | Xho I |
| PR015 | GTGCCGCGCGGCAGCCATATGATGACAGAAAACTTTATTTGGGTAGAA | pET-ArcR; fw | Nde I |
| PR016 | GTGGTGGTGGTGGTGCTCGAGTTAAACACATACATCATTGAATAAATGTTT | pET-ArcR; rv | Xho I |
| PR017 | GTGCCGCGCGGCAGCCATATGATGGCAATTACAAAAATCAATGATTG | pET-SarA; fw | Nde I |

| | | | |
|---|---|---|---|
| PR018 | GTGGTGGTGGTGGTGCTCGAGTTATAGTTCAATTTCGTTGTTTGCTTC | pET-SarA; rv | Xho I |
| PR019 | GTGCCGCGCGGCAGCCATATGATGAGTAAAATTAATGACATTAATGATTTAGTC | pET-SarR; fw | Nde I |
| PR020 | GTGGTGGTGGTGGTGCTCGAGTTAATTTTTAATGTATTCTTCTAATTCTGAAAT | pET-SarR; rv | Xho I |
| PR087 | TAAAGACGATCCGGGGAATTCAAAGTTTAATAAATGTCAAGTCAAT | P*crispr1-lacZ*- pOS1; fw | EcoR I |
| PR088 | CGTTGTAAAACGACGGGATCCACTCATAGATACTTTAATAAATAGCATAACC | P*crispr1-lacZ*- pOS1; rv | BamH I |
| PR089 | TAAAGACGATCCGGGGAATTCTAAATTTAATTACACTCTAAAATTTGTAAATTTT TAATGGAATACGCATTGATTAATTTTTAGGGGATGAAAAATGAAA | P*cas-lacZ*-73-1; fw | EcoR I |
| PR090 | CGTTGTAAAACGACGGGATCCTTTCATTTTTCATCCCCTAAAAATTAATCAATGC GTATTCCATTAAAAATTTACAAATTTTAGAGTGTAATTAAATTTA | P*cas-lacZ*-73-1; rv | BamH I |
| PR091 | TAAAGACGATCCGGGGAATTCATGAAAGATGTTATTTATGTAGAAAATCATTA | P*cas-lacZ*+1+597; fw | EcoR I |
| PR092 | TAAAGACGATCCGGGGAATTCAGAACTAGAGATAGAATTTGG | P*cas-lacZ*+298+597; fw | EcoR I |
| PR093 | TAAAGACGATCCGGGGAATTCGCACAGGCTGCAAGAATTTATTT | P*cas-lacZ*+448+597; fw | EcoR I |
| PR094 | CGTTGTAAAACGACGGGATCCACTCATTTCGAATCCATGTAAAGCT | P*cas-lacZ*- pOS1; rv | BamH I |
| PR111 | ACCGTTAACAGATCTCTGAACAGTAGTTATTA | pCRISPR (leader)-pRAB11; fw | Bgl II |
| PR112 | ATTCTCGTCCCCTGTTATTCGGGGTAGTTATCGATCAGATACTTTAATAAATAGCA TAACC | pCRISPR (leader)-pRAB11; rv | |
| PR113 | GATCGATAACTACCCCGAATAACAGGGGACGAGAATTCCTGCTTCTTCTAATAA CTTTAACATTTGACC | pCRISPR (*gp036*)-pRAB11; fw | |
| PR114 | ACGGCCAGTGAATTCATTCTCGTCCCCTGTTATTCGGGGTAGTTATCGATCGGTC AAATGTTAAAGTTATTAGAAGAAGCAGGA | pCRISPR (*gp036*)-pRAB11; rv | EcoR I |
| PR115 | CAGATAATCTAGGTATCGGAATTAATGAACTTATAGAA | pCR1SP1 | Kpn I |
| PR116 | GATCTTCTATAAGTTCATTAATTCCGATACCTAGATTATCTGGTAC | pCR1SP1 | Bgl II |
| PR127 | CCCTTTCGTCTTCAAGAATTCATGAAAGATGTTATTTATGTAGAAAATCATTA | p*cas1*+1+597 | EcoR I |

| | | | |
|---|---|---|---|
| PR128 | CTTGCATGCCTGCAGGTCGACTTCGAATCCATGTAAAGCTAGTTCTT | p*cas1*+1+597 | Sal I |
| PR187 | CCCTTTCGTCTTCAAGAATTCGGAAGAGTTAAGCTATAACAAAGAATCTCT | P*sarA*-*sarA* product; fw | EcoR I |
| PR188 | CAGTCAAATGAATGACTATGTTATAGTTCAATTTCGTTGTTTGCTTC | P*sarA*-*sarA* product; rv | |
| PR189 | CATAGTCATTCATTTGACTGTATAAAAAACT | P*arcR* product; fw | |
| PR190 | CTGTCATGTCTATTTCCTCCTTTTATCTTTGAATTC | P*arcR* product; rv | |
| PR191 | GGAGGAAATAGACATGACAGAAAACTTTATTTTGGGTAGAA | *arcR* product; fw | |
| PR192 | CTTGCATGCCTGCAGGTCGACTTAAACACATACATCATTGAATAAATGTTT | *arcR* product; rv | Sal I |
| PR193 | TCCCCCGGGCTGCAGGAATTCGCCTTACGACCTCTCTAAGCTAATC | P*gmk* product; fw | EcoR I |
| PR194 | GCCATAATTTATCTTCCACCTTCTTCATATCAT | P*gmk* product; rv | |
| PR195 | GGTGGAAGATAAATTATGGCAATTACAAAAATCAATGATTG | *sarA* product; fw | |
| PR196 | CTTAGAGAGGTCGTAAGGCTTATAGTTCAATTTCGTTGTTTGCTTC | *sarA* product; rv | |
| PR197 | AGCCTTACGACCTCTCTAAGCTAATC | P*gmk* product; fw | |
| PR198 | GTCATAATTTATCTTCCACCTTCTTCATATCAT | P*gmk* product; rv | |
| PR199 | GGTGGAAGATAAATTATGACAGAAAACTTTATTTTGGGTAGAA | *arcR* product; fw | |
| PR200 | GGGCCCCCCCTCGAGGTCGACTTAAACACATACATCATTGAATAAATGTTT | *arcR* product; rv | Sal I |
| **EMSAs probes** | | | |
| PE001 | AAAGTTTAATAAATGTCAAGTCAAT | P*crispr1* probe, FAM-5'-end-labeled; fw | |
| PE002 | AGATACTTTAATAAATAGCATAACC | P*crispr1* probe; rv | |
| PE003 | AAAGTTTAATAAATGTCAAGTCAAT | P*crispr1* probe; fw | |
| PE005 | TACATTTAACAGTTAAGTATTTATTTCCTACAGTTAGGCAATATAATG | P2 promoter probe, FAM-5'-end-labeled; fw | |
| PE006 | CATTATATTGCCTAACTGTAGGAAATAAATACTTAACTGTTAAATGTA | P2 promoter probe; rv | |
| PE007 | ACTAGGGATGCGTTTGAAGC | Gmk probe, FAM-5'- | |

| | | |
|---|---|---|
| | | end-labeled; fw |
| PE008 | GTGTTCTAAACTTGGAGGTGCT | Gmk probe; rv |
| PE017 | GGGAGAACTAGAGATAGAATTTGG | P*cas* probe, FAM-5’-end-labeled; fw |
| PE018 | CGAACGATGATTAATGCCTAAACTC | P*cas* probe; rv |
| PE019 | GGGAGAACTAGAGATAGAATTTGG | P*cas* probe; fw |
| PE020 | GACTAAACCAAATGCTAACCCAG | P*sarA* probe, FAM-5’-end-labeled; fw |
| PE021 | GTGACCATTGATAACAACTCAAAGC | P*sarA* probe; rv |
| PE022 | GACTAAACCAAATGCTAACCCAG | P*sarA* probe; fw |
| PE023 | GCCTCAAATGATATGGCAGA | P*arcR* probe, FAM-5’-end-labeled; fw |
| PE024 | AAAGCTCCAATTTCGCTATTT | P*arcR* probe; rv |
| PE025 | GCCTCAAATGATATGGCAGA | P*arcR* probe; fw |
| PE026 | AAAGTTTAATAAATGTCAAGTCAAT | P*crispr1*-276-177 probe, FAM-5’-end-labeled; fw |
| PE027 | CATATATAATAAGTATTTAATTTTAATATATTTAAACTAATAATAATC | P*crispr1*-276-177 probe; rv |
| PE028 | TTGATTATTATTAGTTTAAATATATTAAAATTAAATACTT | P*crispr1*-226-127 probe, FAM-5’-end-labeled; fw |
| PE029 | TAACTACTGTTCAGGAAAATAAAAAATATACTTC | P*crispr1*-226-127 probe; rv |
| PE030 | GTTCTTTACTATTTTAGAAGTATATTTTTTATTTTCCTG | P*crispr1*-176-77 probe, FAM-5’-end-labeled; |

| | | |
|---|---|---|
| | | fw |
| PE031 | TAAATTTAACTTTTTCTTCCTTTCTAATTTAA | P*crispr1*-176-77 probe; rv |
| PE032 | TTAAATTTATCTCTTATTTTAAATTAGAAAGGA | P*crispr1*-126-27 probe, FAM-5'-end-labeled; fw |
| PE033 | AGTATACTACATTTAATACGTTAATAATTAATCATATATAA | P*crispr1*-126-27 probe; rv |
| PE034 | TATTAAGTTTTATATATGATTAATTATTAACGTATTAAA | P*crispr1*-76+24 probe, FAM-5'-end-labeled; fw |
| PE035 | GAAATGTCACTTTTTTTGACAAATATTATG | P*crispr1*-76+24 probe; rv |
| PE036 | CTTAATATATAGAAAATTATCATAATATTTGTCAAAA | P*crispr1*-26+74 probe, FAM-5'-end-labeled; fw |
| PE037 | TGTTATTTTGGCTGTCAAAAACTGT | P*crispr1*-26+74 probe; rv |
| PE038 | ATATAGTAGCATGGTGTTAGTTGTCACAG | P*crispr1*+25+112 probe, FAM-5'-end-labeled; fw |
| PE039 | AGATACTTTAATAAATAGCATAACCACAAAGG | P*crispr1*+25+112 probe; rv |
| PE040 | GGGAGAACTAGAGATAGAATTTGG | P*cas*-241-142 probe, FAM-5'-end-labeled; fw |

| | | |
|---|---|---|
| PE041 | ATAACTTCACATTCTCATTATGTAAATTGTT | P*cas*-241-142 probe; rv |
| PE042 | TCAATCAAAATGTTTAGAAAACAATTTAC | P*cas*-191-92 probe, FAM-5’-end-labeled; fw |
| PE043 | TTCTTTATTACTTTTATCTCCAGAACTAACA | P*cas*-191-92 probe; rv |
| PE044 | TAGTAAACTTAGCAAAAGATGTTAGTTCTGG | P*cas*-141-42 probe, FAM-5’-end-labeled; fw |
| PE045 | TTAAATTGTTTACCGTATAAATCTTTAAAATA | P*cas*-141-42 probe; rv |
| PE046 | GCACAGGCTGCAAGAATTTATTT | P*cas*-91+8 probe, FAM-5’-end-labeled; fw |
| PE047 | AATACCCATAATTCAACCCTGAGTTA | P*cas*-91+8 probe; rv |
| PE048 | ACGTGGACGGTACAATGATATTATTAA | P*cas*-41+59 probe, FAM-5’-end-labeled; fw |
| PE049 | TTCGAATCCATGTAAAGCTAGTTCTT | P*cas*-41+59 probe; rv |
| PE050 | CTTAATATATAGAAAATTATCATAATATTTGTCAAAAAAAGTGACATTTC | P*crispr1*-26+24 probe, FAM-5’-end-labeled; fw |
| PE051 | GAAATGTCACTTTTTTTGACAAATATTATGATAATTTTCTATATATTAAG | P*crispr1*-26+24 probe; rv |
| PE052 | GTGAAGTTATTAGTAAACTT | P*cas*-151-132 probe, FAM-5’-end-labeled; fw |
| PE053 | AAGTTTACTAATAACTTCAC | P*cas*-151-132 probe; rv |

| | | |
|---|---|---|
| PE054 | AGAATGTGAAGTTATTAGTAAACTTAGCAA | P*cas*-156-127 probe, FAM-5'-end-labeled; fw |
| PE055 | TTGCTAAGTTTACTAATAACTTCACATTCT | P*cas*-156-127 probe; rv |
| PE056 | TAATGAGAATGTGAAGTTATTAGTAAACTTAGCAAAAGAT | P*cas*-161-122 probe, FAM-5'-end-labeled; fw |
| PE057 | ATCTTTTGCTAAGTTTACTAATAACTTCACATTCTCATTA | P*cas*-161-122 probe; rv |
| PE058 | TGTCAAAAAAAGTGACA | P*crispr1*+4+20 probe, FAM-5'-end-labeled; fw |
| PE059 | TGTCACTTTTTTTGACA | P*crispr1*+4+20 probe; rv |
| PE060 | TGTGAAGTTATTAGTA | P*cas*-152-137 probe, FAM-5'-end-labeled; fw |
| PE061 | TACTAATAACTTCACA | P*cas*-152-137 probe; rv |
| **DNA pull-down probes** | | |
| PD001 | AAAGTTTAATAAATGTCAAGTCAAT | P*crispr1* probe, 5'-biotin-labeled; fw |
| PD003 | ACTAGGGATGCGTTTGAAGC | *gmk* probe, 5'-biotin-labeled; fw |
| PD005 | GGGAGAACTAGAGATAGAATTTGG | P*cas* probe, 5'-biotin-labeled; fw |
| **qRT-PCR analysis** | | |
| PQ001 | TGGCGCATGGGATGACATTA | *cas10*; fw |

| | | |
|---|---|---|
| PQ002 | TGAACATCCCAACACCAGCA | *cas10*; rv |
| PQ003 | AATGACTACCTTGGTGGCGG | *csm3*; fw |
| PQ004 | TCTCCAACAACCGTCTCGAT | *csm3*; rv |
| PQ015 | AAAAGATACAATCCTGTGATCG | *spc6*; fw |
| PQ016 | TCGATCGGAATACATCGACC | *spc6*; rv |
| PQ019 | ACGTAATGAGCATGATGAAAGAACT | *sarA*; fw |
| PQ020 | TGTTTGCTTCAGTGATTCGTTT | *sarA*; rv |
| PQ021 | CGGGAAATCCCAGCCATATCA | *arcR*; fw |
| PQ022 | TGTCAGACAGTAGGGTACGA | *arcR*; rv |
| PQ023 | ACTGATGCCGATGTGGA | *gyrB*; fw |
| PQ024 | AACGGTGGCTGTGCAATA | *gyrB*; rv |
| PQ025 | GACGCGATATGATTCTAATTGGTCTTC | *crispr2*; fw |
| PQ026 | GTGGTCCAGAATTAATACATGGTAAAGGAG | *crispr2*; rv |
| PQ029 | GCCCTCGCAACTGATAATCCT | *agrA*; fw |
| PQ030 | CCAACTGGGTCATGCTTACG | *agrA*; rv |
| **co-transcriptional analysis** | | |
| PC001 | GGGGACGAGAACTCATCTTTCATGTCACTG | intergenic regions 1; fw |
| PC002 | CTTGCAGCCTGTGCTTCTTT | intergenic regions 1; rv |
| PC003 | AAAGAAGCACAGGCTGCAAG | intergenic regions 2; fw |
| PC004 | TCGCACAACAACCTTAACCTC | intergenic regions 2; rv |
| PC005 | CGAGAGGTATGTCAGCGATGT | intergenic regions 3; fw |
| PC006 | ATTCTTCGTCGCCCTCTTCT | intergenic regions 3; rv |
| PC007 | TAGCACTTTGGGTGCAAGAA | intergenic regions 4; fw |
| PC008 | GTCGGTTCACTTGCTCCATC | intergenic regions 4; rv |
| PC009 | GGAGCAAGTGAACCGACTTT | intergenic regions 5; fw |

| | | |
|---|---|---|
| PC010 | CGCCACCAAGGTAGTCATTC | intergenic regions 5; rv |
| PC011 | CGAGACGGTTGTTGGAGAAT | intergenic regions 6; fw |
| PC012 | CTTCAGCGCTTAACTCACCA | intergenic regions 6; rv |
| PC013 | CGGCAATGGCTAAAGAAG | intergenic regions 7; fw |
| PC014 | AGGCTCTGAATCGCTGACTT | intergenic regions 7; rv |
| PC015 | CGGTATGCCCGATGTTCTAA | intergenic regions 8; fw |
| PC016 | GACTCCATTTGTGGTGTTCC | intergenic regions 8; rv |
| PC017 | GTGGGTCATTGGCCTACTCT | intergenic regions 9; fw |
| PC018 | CCCCATGCCTAAGCTTGTTT | intergenic regions 9; rv |

*The USR/DSR represents the primers used to amplified the upstream or downstream region of interest gene.

**Supplementary Table S3. Proteins interacting with P*crispr1* sequence of mass spectrometry analysis from DNA pull-down.** Proteins listed are mass spectrometry analysis eluted from 750 mM NaCl in the DNA pull-down. No. unique peptide represents the number of peptides that have similarity to the protein.

| **Protein ID** | **Protein** | **Description** | **Mass (kDa)** | **Coverage (%)** | **Scores** | **No. unique peptide** |
|---|---|---|---|---|---|---|
| TZ0912_GM002240 | SarA | A transcriptional regulator, involved in the QS regulation. | 14.7 | 73.39 | 52.61 | 27 |
| TZ0912_GM002752 | ArcR | A CRP-like transcriptional regulator, involved in the regulation of arginine catabolism. | 27.4 | 35.47 | 20.89 | 11 |
| TZ0912_GM002569 | Unknown | 5'-nucleotidase, lipoprotein e(P4) family. | 33.3 | 18.92 | 15.43 | 8 |
| TZ0912_GM002504 | HipB | A helix-turn-helix domain-containing transcriptional regulator. | 27.6 | 30.17 | 13.12 | 7 |
| TZ0912_GM002755 | ArcB | Ornithine carbamoyltransferase, reversibly catalyzes the transfer of the carbamoyl group from carbamoyl phosphate to the N atom of ornithine to produce L-citrulline. | 37.8 | 16.67 | 12.15 | 7 |
| TZ0912_GM000555 | SarR | A transcriptional regulator, involved in the QS regulation. | 13.7 | 40.00 | 11.14 | 5 |
| TZ0912_GM002083 | GapA | Glyceraldehyde-3-phosphate dehydrogenase, catalyzes the oxidative phosphorylation of glyceraldehyde 3-phosphate (G3P) to 1,3-bisphosphoglycerate using the cofactor NAD. | 36.3 | 13.39 | 6.76 | 4 |
| TZ0912_GM001670 | MraZ | A transcriptional regulator. | 17.0 | 17.02 | 4.97 | 3 |
| TZ0912_GM001595 | TopA | DNA topoisomerase 1, releases the supercoiling and torsional tension of DNA. | 79.3 | 3.04 | 4.31 | 2 |
| TZ0912_GM000740 | FbA | Fructose-bisphosphate aldolase, synthesizes D-glyceraldehyde 3-phosphate and glycerone phosphate from D-glucose. | 30.8 | 7.34 | 4 | 2 |
| TZ0912_GM002646 | IdH 1 | L-lactate dehydrogenase 1, catalyzes the conversion of | 34.6 | 7.26 | 3.28 | 2 |

| | | | | | | |
|---|---|---|---|---|---|---|
| | | lactate to pyruvate. | | | | |
| TZ0912_GM000766 | FabZ | 3-hydroxyacyl-[acyl-carrier-protein] dehydratase, involved in unsaturated fatty acids biosynthesis. | 16.1 | 18.49 | 3.16 | 3 |
| TZ0912_GM002079 | EnO | Enolase, catalyzes the reversible conversion of 2-phosphoglycerate into phosphoenolpyruvate. | 47.1 | 2.77 | 2.02 | 1 |
| TZ0912_GM001253 | RpsU | 30S ribosomal protein S21. | 69.7 | 13.79 | 2 | 1 |
| TZ0912_GM001490 | TkT | Transketolase, catalyzes the transfer of a two-carbon ketol group from a ketose donor to an aldose acceptor. | 72.2 | 1.81 | 2 | 1 |
| TZ0912_GM002081 | TpiA | Triosephosphate isomerase, catalyzes stereospecifically the conversion of dihydroxyacetone phosphate to G3P. | 31.0 | 3.85 | 1.89 | 1 |
| TZ0912_GM001076 | RoT | A transcriptional regulator, involved in the QS regulation. | 19.4 | 4.82 | 1.7 | 1 |

**Supplementary Table S4. Proteins interacting with P*cas* sequence of mass spectrometry analysis from DNA pull-down.** Proteins listed are mass spectrometry analysis eluted from 500 mM NaCl in the DNA pull-down. No. unique peptide represents the number of peptides that have similarity to the protein.

| Protein ID | Protein | Description | Mass (kDa) | Coverage (%) | Scores | No. unique peptide |
|---|---|---|---|---|---|---|
| TZ0912_GM001477 | ParC | DNA topoisomerase 4 subunit A, essential for chromosome segregation. | 91.0 | 76.63 | 176.23 | 103 |
| TZ0912_GM001148 | PolA | DNA polymerase I. In addition to polymerase activity, this DNA polymerase exhibits 5'-3' exonuclease activity. | 99.2 | 70.66 | 109.39 | 55 |
| TZ0912_GM002322 | RpoC | DNA-directed RNA polymerase subunit beta', catalyzes the transcription of DNA into RNA using the four ribonucleoside triphosphates as substrates. | 135.4 | 41.84 | 91.31 | 44 |
| TZ0912_GM002569 | Unknown | 5'-nucleotidase, lipoprotein e(P4) family. | 33.4 | 76.35 | 89.07 | 63 |
| TZ0912_GM002752 | ArcR | A CRP-like transcriptional regulator, involved in the regulation of arginine catabolism. | 27.4 | 83.33 | 67.15 | 50 |
| TZ0912_GM002323 | RpoB | DNA-directed RNA polymerase subunit beta, catalyzes the transcription of DNA into RNA. | 133.2 | 29.92 | 57.97 | 30 |
| TZ0912_GM000743 | AldH1 | 4,4'-diaponeurosporen-aldehyde dehydrogenase, involved in the biosynthesis of the yellow-orange carotenoid staphyloxanthin. | 51.7 | 48.15 | 34.01 | 17 |
| TZ0912_GM000912 | LigA | DNA ligase, essential for DNA replication and repair of damaged DNA. | 75.1 | 34.48 | 33.52 | 19 |
| TZ0912_GM002240 | SarA | A transcriptional regulator, involved in the QS regulation. | 14.7 | 77.42 | 31.61 | 21 |
| TZ0912_GM001125 | HsdM | Type I restriction-modification system subunit M. | 66.9 | 29.71 | 29.99 | 16 |
| TZ0912_GM000392 | Unknown | DUF1801 domain-containing protein. | 23.8 | 71.36 | 28.02 | 14 |

| | | | | | | |
|---|---|---|---|---|---|---|
| TZ0912_GM002167 | Unknown | Deoxyribodipyrimidine photolyase. | 54.4 | 42.45 | 24.94 | 13 |
| TZ0912_GM000130 | SarS | A transcriptional regulator, involved in the QS regulation. | 29.9 | 49.60 | 24.86 | 14 |
| TZ0912_GM000534 | Unknown | A phosphosugar-binding transcriptional regulator, belong to RpiR family. | 33.0 | 45.86 | 21.57 | 11 |
| TZ0912_GM001122 | ThiI | Probable tRNA sulfurtransferase, catalyzes the ATP-dependent transfer of a sulfur to tRNA to produce 4-thiouridine in position 8 of tRNAs. | 46.2 | 26.78 | 19.55 | 11 |
| TZ0912_GM001478 | ParE | DNA topoisomerase 4 subunit B, essential for chromosome segregation. | 74.4 | 14.63 | 18.57 | 10 |
| TZ0912_GM000623 | RpoA | DNA-directed RNA polymerase subunit alpha, catalyzes the transcription of DNA into RNA. | 35.0 | 33.76 | 18.17 | 10 |
| TZ0912_GM002443 | HsdS | Type I restriction modification system subunit S. | 47.1 | 26.55 | 18.05 | 10 |
| TZ0912_GM000555 | SarR | A transcriptional regulator, involved in the QS regulation. | 13.7 | 58.26 | 16.51 | 9 |
| TZ0912_GM002172 | MgrA | A transcriptional regulator, involved in autolytic activity, multidrug resistance and virulence regulation. | 17.1 | 57.14 | 16.23 | 9 |
| TZ0912_GM001271 | NfO | Probable endonuclease 4, plays a role in DNA repair. | 33.2 | 29.05 | 15.5 | 8 |
| TZ0912_GM001157 | InfC | Translation initiation factor IF-3. | 20.2 | 44.57 | 13.23 | 7 |
| TZ0912_GM000766 | FabZ | 3-hydroxyacyl-[acyl-carrier-protein] dehydratase, involved in unsaturated fatty acids biosynthesis. | 16.1 | 50.68 | 12.37 | 6 |
| TZ0912_GM001741 | Unknown | YktB family protein. | 24.0 | 37.25 | 12.06 | 6 |
| TZ0912_GM000824 | Rex | Redox-sensing transcriptional repressor Rex, in response to changes in cellular NADH/$NAD^+$ redox state. | 23.6 | 35.55 | 11.83 | 7 |
| TZ0912_GM000299 | Unknown | Pyruvate oxidase. | 63.8 | 13.64 | 11.61 | 7 |
| TZ0912_GM001267 | SigA | RNA polymerase sigma factor SigA, promote the attachment of RNA polymerase to specific initiation sites. | 42.1 | 23.10 | 11.5 | 6 |
| TZ0912_GM002098 | UvrA | UvrABC system protein A, is an ATPase and a DNA- | 105.3 | 8.54 | 11.27 | 6 |

| | | | | | | |
|---|---|---|---|---|---|---|
| | | binding protein. | | | | |
| TZ0912_GM000525 | Unknown | A transcriptional regulator, Hex regulon repressor. | 29.8 | 24.41 | 10.8 | 6 |
| TZ0912_GM000462 | SarZ | HTH-type transcriptional regulator SarZ, involved in the regulation of virulence genes. | 17.4 | 45.27 | 10.74 | 7 |
| TZ0912_GM002026 | Unknown | arsenate reductase family protein. | 13.6 | 66.10 | 10.2 | 6 |
| TZ0912_GM001100 | Unknown | Uncharacterized protein. | 55.1 | 10.89 | 10.03 | 5 |
| TZ0912_GM001076 | RoT | A transcriptional regulator, involved in the QS regulation. | 19.4 | 31.33 | 9.35 | 5 |
| TZ0912_GM001332 | XerD | Tyrosine recombinase XerD, acts by catalyzing the cutting and rejoining of the recombining DNA molecules. | 34.1 | 17.63 | 9.06 | 5 |
| TZ0912_GM000296 | Unknown | A transcriptional regulator, HTH lysR-type domain-containing protein. | 31.5 | 18.91 | 8 | 4 |
| TZ0912_GM001618 | FapR | Transcriptional regulator FapR, involved in regulation of membrane lipid biosynthesis. | 22.0 | 27.89 | 7.06 | 5 |
| TZ0912_GM001301 | Unknown | Biotin carboxyl carrier protein of acetyl-CoA carboxylase, is a component of the acetyl coenzyme A carboxylase complex. | 17.1 | 28.57 | 6.04 | 4 |
| TZ0912_GM001356 | HbsU | A transcriptional regulator, DNA-binding protein HU. | 9.6 | 51.11 | 6 | 3 |
| TZ0912_GM000006 | GyrA | DNA gyrase subunit A. | 99.3 | 3.61 | 5.44 | 3 |
| TZ0912_GM001636 | PriA | Primosomal protein N, involved in the restart of stalled replication forks. | 92.5 | 3.74 | 4.68 | 3 |
| TZ0912_GM000977 | Unknown | A transcriptional regulator, HTH cro/C1-type domain-containing protein. | 17.8 | 15.58 | 4.29 | 2 |
| TZ0912_GM000582 | SarV | A transcriptional regulator SarV, involved in the regulation of virulence genes. | 14.0 | 24.14 | 4.01 | 3 |
| TZ0912_GM001545 | GlpD | Aerobic glycerol-3-phosphate dehydrogenase. | 62.3 | 5.74 | 3.4 | 3 |
| TZ0912_GM000324 | Unknown | A transcriptional regulator, iron dependent repressor, HTH | 18.1 | 19.74 | 2.98 | 3 |

| | | | | | | |
|---|---|---|---|---|---|---|
| | | marR-type domain-containing protein. | | | | |
| TZ0912_GM001595 | TopA | DNA topoisomerase 1, DNA topological change. | 79.1 | 1.89 | 2 | 1 |
| TZ0912_GM001576 | InfB | Translation initiation factor IF-2, one of the essential components for the initiation of protein synthesis. | 77.9 | 1.28 | 2 | 1 |
| TZ0912_GM001873 | ClpB | Chaperone protein ClpB, involved in the recovery of the cell from heat-induced damage. | 98.3 | 1.50 | 2 | 1 |
| TZ0912_GM002190 | SarX | A transcriptional regulator SarX, involved in the regulation of virulence genes. | 14.2 | 7.56 | 2 | 1 |
| TZ0912_GM000490 | TcaR | Teicoplanin-resistance associated HTH-type transcriptional regulator TcaR. | 9.0 | 14.10 | 2 | 1 |
| TZ0912_GM001187 | RuvA | Holliday junction ATP-dependent DNA helicase RuvA. | 22.3 | 5.50 | 1.62 | 1 |

## References


1. Monk, I.R., Tree, J.J., Howden, B.P., Stinear, T.P. and Foster, T.J. (2015) Complete Bypass of Restriction Systems for Major *Staphylococcus aureus* Lineages. *mBio*, **6**, e00308-00315.
2. Li, Y., Mikkelsen, K., Lluch, I.G.O., Wang, Z., Tang, Y., Jiao, X., Ingmer, H., Hoyland-Kroghsbo, N.M. and Li, Q. (2021) Functional characterization of Type III-A CRISPR-Cas in a clinical human Methicillin-R *Staphylococcus aureus* strain. *CRISPR J*, **4**, 686-698.
3. Golding, G.R., Bryden, L., Levett, P.N., McDonald, R.R., Wong, A., Wylie, J., Graham, M.R., Tyler, S., Van Domselaar, G., Simor, A.E. *et al.* (2010) Livestock-associated methicillin-resistant *Staphylococcus aureus* sequence type 398 in humans, Canada. *Emerging Infect Dis*, **16**, 587-594.
4. Gutiérrez, D., Vandenheuvel, D., Martínez, B., Rodríguez, A., Lavigne, R., García, P. and Wommack, K.E. (2015) Two phages, phiIPLA-RODI and phiIPLA-C1C, lyse mono- and dual-species staphylococcal biofilms. *Appl Environ Microbiol*, **81**, 3336-3348.

5. Takeuchi, I., Osada, K., Azam, A.H., Asakawa, H., Miyanaga, K. and Tanji, Y. (2016) The presence of two receptor-binding proteins contributes to the wide host range of staphylococcal twort-like phages. *Appl Environ Microbiol*, **82**, 5763-5774.
6. Monk, I.R., Shah, I.M., Xu, M., Tan, M.-W., Foster, T.J. and Novick, R.P. (2012) Transforming the untransformable: application of direct transformation to manipulate genetically *Staphylococcus aureus* and *Staphylococcus epidermidis*. *mBio*, **3**.
7. Corrigan, R.M. and Foster, T.J. (2009) An improved tetracycline-inducible expression vector for *Staphylococcus aureus*. *Plasmid*, **61**, 126-129.
8. Helle, L., Kull, M., Mayer, S., Marincola, G., Zelder, M.-E., Goerke, C., Wolz, C. and Bertram, R. (2011) Vectors for improved Tet repressor-dependent gradual gene induction or silencing in *Staphylococcus aureus*. *Microbiology*, **157**, 3314-3323.
9. Lee, C.Y., Buranen, S.L. and Ye, Z.-H. (1991) Construction of single-copy integration vectors for *Staphylococcus aureus*. *Gene*, **103**, 101-105.
10. Liu, H., Shang, W., Hu, Z., Zheng, Y., Yuan, J., Hu, Q., Peng, H., Cai, X., Tan, L., Li, S. *et al.* (2018) A novel SigB(Q225P) mutation in *Staphylococcus aureus* retains virulence but promotes biofilm formation. *Emerg Microbes Infect*, **7**, 72.
